# Supplementary material for: No evidence that omission and confirmation biases affect the perception and recall of vaccine-related information
Source: PLoS One. 2020 Mar 4;15(3):e0228898. doi: 10.1371/journal.pone.0228898 (PMC7055885; doi:10.1371/journal.pone.0228898)
Supplement: S1 File — (DOCX) [file pone.0228898.s001.docx]

SUPPLEMENTARY MATERIALS FOR “NO EVIDENCE THAT OMISSION AND CONGRUITY BIASES AFFECT THE PERCEPTION AND RECALL OF VACCINE-RELATED INFORMATION”

# A: PRETEST 1

## 1.- INTRODUCTION

The purpose of Pre-test 1 was to select a list of symptoms for the experiment based on people’s familiarity with the symptoms and perceptions of their severity. Lack of familiarity with the symptoms might prompt participants to provide random ratings of severity. Therefore, participants’ familiarity with the symptoms should improve the quality of their answers. Hence, the symptoms to be used in the experiment required to be considerably familiar for the participants. Additionally, the perceptions of the severity of the symptoms for the experiment should vary considerably across individuals. Little variation in participants’ perception of severity in a set of symptoms without a given explicit cause might lead to difficulties in eliciting differences in ratings when symptoms are associated to specific causes (i.e. infectious diseases or vaccine side effects).

## 2.- METHODS

### 2.1- Participants

83 complete responses were collected through the crowdsourcing website Crowdflower. After eliminating duplicate responses, self-reported non-native English speakers and suspicious self-reported English native speakers (i.e. people with non-English speaking nationalities), 58 participants (22 males, 36 females) aged 19-76 (*M*=38.10, *SD*=14.10) were included in the analyses. The rationale for the elimination of non-native English speakers was that these participants might not know the meaning of some of the symptoms included in the list and, therefore, their ratings would not be reliable.

### 2.- Materials, Design and Procedure

An online questionnaire was created with Qualtrics to collect participants’ severity and familiarity ratings of a list of 54 symptoms (table 1). Some of these symptoms were extracted from Brown et al. (2010). The rest of the symptoms were found on a Google search with the terms “vaccine side effects” and “symptoms”. A within-subjects design was used. All participants answered sequentially two questions about each of these symptoms. In the first question participants rated each of the symptoms by its severity on a 7-point Likert scale from 1 (very mild) to 7 (very severe). The second question asked participants to rate their degree of familiarity with each of the symptoms on a 7-point Likert scale from 1 (very unfamiliar) to 7 (very familiar). Sea SM.

*Table 1. List of symptoms for Pre-test 1.*

| **LIST OF SYMPTOMS FOR PRE-TEST 1** | |
| --- | --- |
| **Symptoms selected for the experiment** | crying, shivering, chills, swelling, cough, nausea, vomiting, diarrhoea, headache, earache, wheezing, abdominal pain, muscle ache, loss of appetite, soreness, hoarseness, itchy eyes, toothache, light-headedness, dry mouth, weight loss, anxiety, insomnia, bleeding. |
| **Symptoms selected for the practice** | irritability, fever, rash, sneezing, dizziness |
| **Remaining symptoms** | fatigue, heart palpitations, joint pain, blood in urine, stomach inflammation, tenderness, redness, itching, lump, nasal congestion, seizure, paraesthesia, blindness, blurred vision, double vision, weight gain, chest pain, convulsion, depression, hallucination, paranoia, tics, arrhythmia, pneumonia, deafness |

## 3.- RESULTS

For the election of symptoms, two criteria were followed. The first criterion was that the symptoms were highly familiar to the participants, which was operationalized as “symptoms with an average familiarity rating greater than 4.5”. The second criterion tried to ensure large variability in severity ratings and was operationalized as “symptoms with average standard deviation in severity ratings greater than 1.3”. The application of these two criteria left 31 symptoms. From these, 3 (redness, weight gain and itching) were eliminated to prevent confusions with other symptoms included in the list (rash, weight loss and itchy eyes) and one (depression) was eliminated given the fact that it is highly unlikely that a 1 year old, who was part of the experimental manipulation, might have depression. This left a total of 27 symptoms. As we needed to have some symptoms for the participants to practice with the experimental procedure, 3 symptoms were randomly selected from the remaining symptoms (dizziness, irritability and rash) and 2 other symptoms (fever and sneezing) were selected from the previously excluded symptoms (both with *M*_familiarity_>4.5 and *SD*_severity_>1.2). See table 1 above.

## 4.- DISCUSSION

The pre-test was successful in allowing the selection of a large enough list of suitable symptoms for the practice and the experiment.

# B: R SCRIPT FOR PRETEST 1

Angel V. Jimenez

13 April 2018

#Open Dataset
pretest1<-read.csv("C://Pretest1_dataset.csv") #Dataset after eliminating duplicates, self-reported non-native
# English speakers and suspicious self-reported native English speakers
str(pretest1)

## 'data.frame': 58 obs. of 123 variables:
## $ Answer : int 1 2 3 6 7 8 9 10 12 13 ...
## $ Record_date : Factor w/ 57 levels "Jun 26, 2016 6:41 PM ",..: 1 2 3 4 5 6 7 8 9 10 ...
## $ Progress : int 100 100 100 100 100 100 100 100 100 100 ...
## $ Nationality : Factor w/ 22 levels "\"23\"","\"America\"",..: 11 9 7 22 11 11 7 8 7 11 ...
## $ Crowdflower_ID : Factor w/ 58 levels "ID_1","ID_11",..: 1 5 13 26 35 46 57 58 2 3 ...
## $ IP_Address : Factor w/ 58 levels "IP_1","IP_11",..: 1 5 14 26 37 47 57 58 2 3 ...
## $ Duration_S : int 230 209 404 250 263 356 193 228 216 150 ...
## $ Duration : Factor w/ 55 levels "00:02:30","00:02:44",..: 15 9 42 19 21 37 3 14 12 1 ...
## $ S_Irritability : int 1 1 3 4 1 4 1 2 3 2 ...
## $ S_Crying : int 4 1 4 3 1 5 1 5 2 2 ...
## $ S_Fever : int 6 1 6 5 4 7 3 6 5 6 ...
## $ S_Rash : int 6 1 5 4 4 3 2 6 2 5 ...
## $ S_Shivering : int 6 1 4 5 5 5 2 5 4 4 ...
## $ S_Rash.1 : int 6 1 5 4 4 3 2 6 2 5 ...
## $ S_Chills : int 6 1 5 5 4 5 3 5 3 4 ...
## $ S_Swelling : int 6 1 6 6 6 4 3 5 4 5 ...
## $ S_Cough : int 4 1 5 4 4 4 2 4 2 5 ...
## $ S_Nausea : int 5 1 6 6 5 4 2 4 3 5 ...
## $ S_sneezing : int 2 1 3 4 2 1 2 2 1 2 ...
## $ S_Fatigue : int 5 2 4 6 2 3 3 4 3 5 ...
## $ S_Vomiting : int 6 1 6 7 6 6 3 5 6 6 ...
## $ S_Diarrhea : int 6 1 6 7 6 5 2 5 7 6 ...
## $ S_Headache : int 6 1 4 4 5 4 2 5 4 5 ...
## $ S_Dizziness : int 6 1 6 6 6 6 3 5 5 5 ...
## $ S_Palpitations : int 7 1 7 7 6 7 4 7 7 7 ...
## $ S_Earache : int 3 1 4 5 5 4 4 4 2 5 ...
## $ S_JointPain : int 4 1 4 6 4 4 4 4 3 4 ...
## $ S_Wheezing : int 6 1 5 6 5 7 3 5 4 4 ...
## $ S_AbdominalPain : int 6 1 5 6 6 6 4 5 6 5 ...
## $ S_BloodInUrine : int 7 1 7 7 7 7 5 7 7 7 ...
## $ S_StomachInflmation : int 6 1 6 6 6 7 4 6 6 6 ...
## $ S_Pneumonia : int 7 1 7 7 7 7 6 7 7 7 ...
## $ S_Tenderness : int 3 1 4 3 5 3 4 5 2 3 ...
## $ S_Redness : int 4 1 3 4 3 4 2 4 1 3 ...
## $ S_Itching : int 4 2 5 4 3 4 3 4 1 3 ...
## $ S_Lump : int 7 1 6 5 5 4 4 7 6 5 ...
## $ S_MuscleAche : int 7 1 4 2 4 2 3 4 3 5 ...
## $ S_LossofAppetite : int 5 1 6 4 4 5 1 5 4 4 ...
## $ S_Soreness : int 6 1 2 2 4 4 2 5 2 4 ...
## $ S_Hoarseness : int 3 1 4 3 3 6 2 3 2 4 ...
## $ S_ItchyEyes : int 3 1 2 3 2 2 2 4 2 4 ...
## $ S_NasalCongestion : int 3 1 4 2 2 4 2 4 3 4 ...
## $ S_Seizure : int 7 1 7 7 7 7 6 7 7 7 ...
## $ S_Deafness : int 2 1 5 7 5 7 5 4 4 6 ...
## $ S_Toothache : int 2 1 4 6 5 5 5 4 3 5 ...
## $ S_Paresthesia : int 6 1 7 4 7 5 4 5 3 6 ...
## $ S_Light.headed : int 6 1 4 6 4 5 2 5 3 5 ...
## $ S_DryMouth : int 4 1 3 3 3 2 1 4 3 3 ...
## $ S_Blindness : int 7 1 6 7 7 7 6 7 7 7 ...
## $ S_BlurredVision : int 5 1 5 6 4 7 3 7 5 6 ...
## $ S_DoubleVision : int 5 1 5 6 5 7 3 7 5 6 ...
## $ S_Weightloss : int 4 1 6 7 4 3 1 6 6 4 ...
## $ S_WeightGain : int 4 1 4 6 4 4 1 6 3 4 ...
## $ S_ChestPain : int 7 1 7 7 6 7 3 7 7 6 ...
## $ S_Convulsions : int 7 1 7 7 7 7 2 7 7 6 ...
## $ S_Bleeding : int 7 1 5 5 6 6 5 7 6 4 ...
## $ S_Anxiety : int 5 1 6 4 1 6 1 3 4 4 ...
## $ S_Depression : int 5 1 6 4 2 7 1 3 4 5 ...
## $ S_Hallucinations : int 7 1 7 7 6 7 3 6 6 6 ...
## $ S_Insomnia : int 6 1 4 5 2 6 1 4 5 5 ...
## $ S_Paranoia : int 7 1 6 6 5 7 2 5 5 6 ...
## $ S_Tics : int 4 1 3 3 4 5 2 4 5 4 ...
## $ S_Arrythimia : int 7 1 5 7 5 7 4 7 5 6 ...
## $ F_Irritability : int 1 7 5 6 7 7 7 6 7 4 ...
## $ F_Crying : int 1 7 7 7 7 7 7 6 7 4 ...
## $ F_Fever : int 5 7 4 6 7 3 7 5 7 4 ...
## $ F_Rash : int 1 7 4 5 7 4 7 5 7 4 ...
## $ F_Shivering : int 5 7 5 6 7 4 7 6 6 4 ...
## $ F_Rash.1 : int 1 7 4 5 7 4 7 5 7 4 ...
## $ F_Chills : int 1 7 5 5 7 4 7 6 5 4 ...
## $ F_Swelling : int 1 7 3 5 7 4 7 6 7 4 ...
## $ F_Cough : int 5 7 6 7 7 5 7 6 7 4 ...
## $ F_Nausea : int 5 7 5 7 7 5 7 6 7 4 ...
## $ F_Sneezing : int 5 7 7 7 7 5 7 6 7 4 ...
## $ F_Fatigue : int 5 7 6 7 7 6 7 6 7 4 ...
## $ F_Vomiting : int 5 7 4 7 7 4 7 6 7 4 ...
## $ F_Diarrhoea : int 5 7 5 7 7 7 7 6 7 4 ...
## $ F_Headache : int 5 7 4 7 7 5 7 6 7 4 ...
## $ F_Dizziness : int 5 7 4 7 7 5 7 6 7 4 ...
## $ F_HeartPalpitations : int 1 7 2 6 6 7 7 3 4 4 ...
## $ F_Earache : int 1 7 4 5 7 4 7 5 5 4 ...
## $ F_JointPain : int 1 7 3 5 7 4 7 5 7 4 ...
## $ F_Wheezing : int 1 7 3 5 7 6 7 6 3 4 ...
## $ F_AbdominalPain : int 1 7 6 7 7 4 7 4 7 4 ...
## $ F_BloodinUrine : int 1 7 3 4 7 1 7 5 7 4 ...
## $ F_StomachInflamation: int 1 7 5 5 7 6 7 4 5 4 ...
## $ F_Pneumoina : int 1 7 4 5 7 4 6 3 3 4 ...
## $ F_Tenderness : int 1 7 5 6 7 4 7 5 7 4 ...
## $ F_Redness : int 1 7 4 6 7 4 7 5 7 4 ...
## $ F_Itching : int 1 7 5 7 7 4 7 5 7 4 ...
## $ F_Lump : int 1 7 6 5 7 4 6 4 7 4 ...
## $ F_MuscleAche : int 1 7 4 7 7 7 7 5 7 4 ...
## $ F_LossofAppetitte : int 1 7 5 6 7 4 7 5 7 4 ...
## $ F_Soreness : int 1 7 4 7 7 4 7 5 7 4 ...
## $ F_Hoarseness : int 1 7 3 7 7 4 7 5 5 4 ...
## $ F_ItchyEyes : int 1 7 3 5 7 6 7 5 7 4 ...
## $ F_NasalCongestion : int 5 7 5 5 7 7 7 5 6 4 ...
## $ F_Seizure : int 1 7 3 3 7 2 7 3 7 4 ...
## $ F_Deafness : int 1 7 4 4 7 2 7 5 7 4 ...
## [list output truncated]

summary(pretest1)

## Answer Record_date Progress Nationality
## Min. : 1.00 Jun 28, 2016 4:53 PM : 2 Min. :100 "Canadian":13
## 1st Qu.:27.50 Jun 26, 2016 6:41 PM : 1 1st Qu.:100 "British" :10
## Median :50.50 Jun 26, 2016 7:07 PM : 1 Median :100 "American": 7
## Mean :46.24 Jun 26, 2016 7:13 PM : 1 Mean :100 "british" : 5
## 3rd Qu.:67.75 Jun 26, 2016 9:15 PM : 1 3rd Qu.:100 "canadian": 3
## Max. :83.00 Jun 26, 2016 9:21 PM : 1 Max. :100 "USA" : 3
## (Other) :51 (Other) :17
## Crowdflower_ID IP_Address Duration_S Duration
## ID_1 : 1 IP_1 : 1 Min. : 150.0 00:03:36: 2
## ID_11 : 1 IP_11 : 1 1st Qu.: 228.5 00:04:23: 2
## ID_12 : 1 IP_12 : 1 Median : 284.5 00:06:28: 2
## ID_15 : 1 IP_14 : 1 Mean : 363.6 00:02:30: 1
## ID_2 : 1 IP_2 : 1 3rd Qu.: 398.5 00:02:44: 1
## ID_21 : 1 IP_20 : 1 Max. :1515.0 00:03:13: 1
## (Other):52 (Other):52 (Other) :49
## S_Irritability S_Crying S_Fever S_Rash
## Min. :1.000 Min. :1.000 Min. :1.000 Min. :1.00
## 1st Qu.:1.250 1st Qu.:2.000 1st Qu.:4.000 1st Qu.:3.00
## Median :3.000 Median :3.000 Median :5.000 Median :4.00
## Mean :2.828 Mean :2.931 Mean :4.655 Mean :4.19
## 3rd Qu.:4.000 3rd Qu.:4.000 3rd Qu.:6.000 3rd Qu.:5.00
## Max. :7.000 Max. :7.000 Max. :7.000 Max. :7.00
##
## S_Shivering S_Rash.1 S_Chills S_Swelling
## Min. :1.000 Min. :1.000 Min. :1.000 Min. :1.000
## 1st Qu.:3.000 1st Qu.:3.000 1st Qu.:3.000 1st Qu.:4.000
## Median :4.000 Median :4.000 Median :4.000 Median :4.500
## Mean :4.017 Mean :4.172 Mean :4.017 Mean :4.517
## 3rd Qu.:5.000 3rd Qu.:5.000 3rd Qu.:5.000 3rd Qu.:6.000
## Max. :7.000 Max. :7.000 Max. :7.000 Max. :7.000
##
## S_Cough S_Nausea S_sneezing S_Fatigue
## Min. :1.000 Min. :1.000 Min. :1.000 Min. :1.000
## 1st Qu.:3.000 1st Qu.:3.000 1st Qu.:2.000 1st Qu.:3.000
## Median :4.000 Median :4.000 Median :2.000 Median :4.000
## Mean :3.672 Mean :4.103 Mean :2.724 Mean :3.483
## 3rd Qu.:4.750 3rd Qu.:5.000 3rd Qu.:4.000 3rd Qu.:4.000
## Max. :7.000 Max. :7.000 Max. :7.000 Max. :6.000
##
## S_Vomiting S_Diarrhea S_Headache S_Dizziness
## Min. :1.000 Min. :1.000 Min. :1.000 Min. :1.000
## 1st Qu.:4.000 1st Qu.:4.000 1st Qu.:3.000 1st Qu.:4.000
## Median :5.000 Median :5.000 Median :4.000 Median :5.000
## Mean :4.948 Mean :4.914 Mean :4.052 Mean :4.534
## 3rd Qu.:6.000 3rd Qu.:6.000 3rd Qu.:5.000 3rd Qu.:6.000
## Max. :7.000 Max. :7.000 Max. :7.000 Max. :7.000
##
## S_Palpitations S_Earache S_JointPain S_Wheezing
## Min. :1.000 Min. :1.000 Min. :1.000 Min. :1.000
## 1st Qu.:4.000 1st Qu.:3.000 1st Qu.:3.250 1st Qu.:4.000
## Median :6.000 Median :4.000 Median :4.000 Median :4.000
## Mean :5.448 Mean :3.948 Mean :4.034 Mean :4.483
## 3rd Qu.:7.000 3rd Qu.:5.000 3rd Qu.:5.000 3rd Qu.:5.750
## Max. :7.000 Max. :7.000 Max. :7.000 Max. :7.000
##
## S_AbdominalPain S_BloodInUrine S_StomachInflmation S_Pneumonia
## Min. :1.000 Min. :1.000 Min. :1.00 Min. :1.000
## 1st Qu.:4.000 1st Qu.:5.000 1st Qu.:4.00 1st Qu.:6.000
## Median :5.000 Median :7.000 Median :6.00 Median :6.000
## Mean :4.845 Mean :5.897 Mean :5.31 Mean :6.017
## 3rd Qu.:6.000 3rd Qu.:7.000 3rd Qu.:6.00 3rd Qu.:7.000
## Max. :7.000 Max. :7.000 Max. :7.00 Max. :7.000
##
## S_Tenderness S_Redness S_Itching S_Lump
## Min. :1.000 Min. :1.000 Min. :1.000 Min. :1.000
## 1st Qu.:3.000 1st Qu.:2.000 1st Qu.:2.000 1st Qu.:4.000
## Median :4.000 Median :4.000 Median :3.500 Median :5.000
## Mean :3.483 Mean :3.362 Mean :3.328 Mean :4.707
## 3rd Qu.:4.000 3rd Qu.:4.000 3rd Qu.:4.000 3rd Qu.:6.000
## Max. :6.000 Max. :6.000 Max. :6.000 Max. :7.000
##
## S_MuscleAche S_LossofAppetite S_Soreness S_Hoarseness
## Min. :1.000 Min. :1.000 Min. :1.000 Min. :1.000
## 1st Qu.:3.000 1st Qu.:3.000 1st Qu.:3.000 1st Qu.:2.000
## Median :4.000 Median :4.000 Median :4.000 Median :3.000
## Mean :3.638 Mean :3.862 Mean :3.603 Mean :3.328
## 3rd Qu.:4.000 3rd Qu.:5.000 3rd Qu.:4.750 3rd Qu.:4.000
## Max. :7.000 Max. :7.000 Max. :7.000 Max. :6.000
##
## S_ItchyEyes S_NasalCongestion S_Seizure S_Deafness
## Min. :1.000 Min. :1.000 Min. :1.000 Min. :1.000
## 1st Qu.:2.000 1st Qu.:2.000 1st Qu.:5.250 1st Qu.:4.000
## Median :3.000 Median :3.000 Median :6.000 Median :6.000
## Mean :3.241 Mean :3.259 Mean :5.914 Mean :5.276
## 3rd Qu.:4.000 3rd Qu.:4.000 3rd Qu.:7.000 3rd Qu.:7.000
## Max. :7.000 Max. :6.000 Max. :7.000 Max. :7.000
##
## S_Toothache S_Paresthesia S_Light.headed S_DryMouth
## Min. :1.00 Min. :1.000 Min. :1.000 Min. :1.000
## 1st Qu.:3.00 1st Qu.:4.000 1st Qu.:3.000 1st Qu.:3.000
## Median :4.00 Median :4.000 Median :4.000 Median :3.500
## Mean :4.19 Mean :4.534 Mean :4.138 Mean :3.448
## 3rd Qu.:5.00 3rd Qu.:5.750 3rd Qu.:5.000 3rd Qu.:4.000
## Max. :7.00 Max. :7.000 Max. :7.000 Max. :7.000
##
## S_Blindness S_BlurredVision S_DoubleVision S_Weightloss
## Min. :1.00 Min. :1.000 Min. :1.000 Min. :1.000
## 1st Qu.:5.25 1st Qu.:4.250 1st Qu.:4.000 1st Qu.:3.000
## Median :7.00 Median :5.500 Median :5.000 Median :4.000
## Mean :6.00 Mean :5.276 Mean :5.155 Mean :4.224
## 3rd Qu.:7.00 3rd Qu.:6.000 3rd Qu.:6.000 3rd Qu.:5.000
## Max. :7.00 Max. :7.000 Max. :7.000 Max. :7.000
##
## S_WeightGain S_ChestPain S_Convulsions S_Bleeding
## Min. :1.000 Min. :1.000 Min. :1.000 Min. :1.000
## 1st Qu.:3.000 1st Qu.:5.000 1st Qu.:5.000 1st Qu.:5.000
## Median :4.000 Median :6.000 Median :7.000 Median :6.000
## Mean :4.138 Mean :5.776 Mean :5.845 Mean :5.466
## 3rd Qu.:5.000 3rd Qu.:7.000 3rd Qu.:7.000 3rd Qu.:7.000
## Max. :7.000 Max. :7.000 Max. :7.000 Max. :7.000
##
## S_Anxiety S_Depression S_Hallucinations S_Insomnia
## Min. :1.000 Min. :1.000 Min. :1.000 Min. :1.000
## 1st Qu.:3.000 1st Qu.:4.000 1st Qu.:4.000 1st Qu.:4.000
## Median :4.000 Median :4.500 Median :6.000 Median :4.000
## Mean :4.155 Mean :4.517 Mean :5.362 Mean :4.328
## 3rd Qu.:5.000 3rd Qu.:6.000 3rd Qu.:7.000 3rd Qu.:5.000
## Max. :7.000 Max. :7.000 Max. :7.000 Max. :7.000
##
## S_Paranoia S_Tics S_Arrythimia F_Irritability
## Min. :1.000 Min. :1.000 Min. :1.000 Min. :1.000
## 1st Qu.:4.000 1st Qu.:3.250 1st Qu.:4.250 1st Qu.:4.000
## Median :5.000 Median :4.000 Median :5.000 Median :6.000
## Mean :5.086 Mean :4.345 Mean :5.293 Mean :5.328
## 3rd Qu.:7.000 3rd Qu.:5.000 3rd Qu.:6.750 3rd Qu.:7.000
## Max. :7.000 Max. :7.000 Max. :7.000 Max. :7.000
##
## F_Crying F_Fever F_Rash F_Shivering
## Min. :1.000 Min. :1.000 Min. :1.000 Min. :1.000
## 1st Qu.:4.250 1st Qu.:4.250 1st Qu.:4.000 1st Qu.:4.000
## Median :6.000 Median :6.000 Median :5.000 Median :5.000
## Mean :5.517 Mean :5.517 Mean :5.069 Mean :5.138
## 3rd Qu.:7.000 3rd Qu.:7.000 3rd Qu.:7.000 3rd Qu.:7.000
## Max. :7.000 Max. :7.000 Max. :7.000 Max. :7.000
##
## F_Rash.1 F_Chills F_Swelling F_Cough
## Min. :1.000 Min. :1.000 Min. :1.000 Min. :1.00
## 1st Qu.:4.000 1st Qu.:4.000 1st Qu.:4.000 1st Qu.:5.00
## Median :5.000 Median :5.000 Median :6.000 Median :6.50
## Mean :5.052 Mean :5.069 Mean :5.017 Mean :5.81
## 3rd Qu.:7.000 3rd Qu.:7.000 3rd Qu.:7.000 3rd Qu.:7.00
## Max. :7.000 Max. :7.000 Max. :7.000 Max. :7.00
##
## F_Nausea F_Sneezing F_Fatigue F_Vomiting
## Min. :1.000 Min. :1.000 Min. :2.000 Min. :1.000
## 1st Qu.:4.000 1st Qu.:5.000 1st Qu.:4.000 1st Qu.:4.000
## Median :6.000 Median :7.000 Median :6.000 Median :6.000
## Mean :5.534 Mean :5.828 Mean :5.534 Mean :5.362
## 3rd Qu.:7.000 3rd Qu.:7.000 3rd Qu.:7.000 3rd Qu.:7.000
## Max. :7.000 Max. :7.000 Max. :7.000 Max. :7.000
##
## F_Diarrhoea F_Headache F_Dizziness F_HeartPalpitations
## Min. :2.00 Min. :1.000 Min. :1.000 Min. :1.00
## 1st Qu.:4.00 1st Qu.:4.000 1st Qu.:4.000 1st Qu.:3.00
## Median :6.00 Median :6.000 Median :6.000 Median :4.00
## Mean :5.69 Mean :5.569 Mean :5.276 Mean :4.19
## 3rd Qu.:7.00 3rd Qu.:7.000 3rd Qu.:7.000 3rd Qu.:6.00
## Max. :7.00 Max. :7.000 Max. :7.000 Max. :7.00
##
## F_Earache F_JointPain F_Wheezing F_AbdominalPain
## Min. :1.000 Min. :1.00 Min. :1.000 Min. :1.000
## 1st Qu.:4.000 1st Qu.:3.25 1st Qu.:3.000 1st Qu.:4.000
## Median :5.000 Median :5.00 Median :5.000 Median :5.000
## Mean :4.707 Mean :4.81 Mean :4.534 Mean :4.914
## 3rd Qu.:6.000 3rd Qu.:7.00 3rd Qu.:6.750 3rd Qu.:7.000
## Max. :7.000 Max. :7.00 Max. :7.000 Max. :7.000
##
## F_BloodinUrine F_StomachInflamation F_Pneumoina F_Tenderness
## Min. :1.00 Min. :1.000 Min. :1.000 Min. :1.000
## 1st Qu.:1.00 1st Qu.:3.000 1st Qu.:1.500 1st Qu.:4.000
## Median :3.50 Median :4.000 Median :4.000 Median :5.000
## Mean :3.69 Mean :4.345 Mean :4.052 Mean :4.793
## 3rd Qu.:6.00 3rd Qu.:7.000 3rd Qu.:6.000 3rd Qu.:7.000
## Max. :7.00 Max. :7.000 Max. :7.000 Max. :7.000
##
## F_Redness F_Itching F_Lump F_MuscleAche
## Min. :1.000 Min. :1.000 Min. :1.000 Min. :1.000
## 1st Qu.:4.000 1st Qu.:4.000 1st Qu.:2.000 1st Qu.:4.000
## Median :5.000 Median :5.000 Median :4.000 Median :5.500
## Mean :4.724 Mean :5.155 Mean :4.017 Mean :5.276
## 3rd Qu.:6.750 3rd Qu.:7.000 3rd Qu.:6.000 3rd Qu.:7.000
## Max. :7.000 Max. :7.000 Max. :7.000 Max. :7.000
##
## F_LossofAppetitte F_Soreness F_Hoarseness F_ItchyEyes
## Min. :1.000 Min. :1.000 Min. :1.000 Min. :1.000
## 1st Qu.:4.000 1st Qu.:4.000 1st Qu.:3.250 1st Qu.:4.000
## Median :4.500 Median :5.000 Median :4.000 Median :5.000
## Mean :4.724 Mean :5.138 Mean :4.621 Mean :4.983
## 3rd Qu.:7.000 3rd Qu.:7.000 3rd Qu.:7.000 3rd Qu.:7.000
## Max. :7.000 Max. :7.000 Max. :7.000 Max. :7.000
##
## F_NasalCongestion F_Seizure F_Deafness F_Toothache
## Min. :1.000 Min. :1.0 Min. :1.000 Min. :1.000
## 1st Qu.:4.000 1st Qu.:1.0 1st Qu.:1.000 1st Qu.:4.000
## Median :6.000 Median :3.0 Median :4.000 Median :5.000
## Mean :5.466 Mean :3.5 Mean :3.759 Mean :4.828
## 3rd Qu.:7.000 3rd Qu.:5.0 3rd Qu.:6.000 3rd Qu.:7.000
## Max. :7.000 Max. :7.0 Max. :7.000 Max. :7.000
##
## F_Paresthesia F_Lightheaded F_DryMouth F_Blindness
## Min. :1.000 Min. :1.000 Min. :1.000 Min. :1.000
## 1st Qu.:1.000 1st Qu.:3.000 1st Qu.:4.000 1st Qu.:1.000
## Median :1.500 Median :4.000 Median :4.000 Median :3.500
## Mean :2.638 Mean :4.534 Mean :4.759 Mean :3.638
## 3rd Qu.:4.000 3rd Qu.:7.000 3rd Qu.:7.000 3rd Qu.:6.750
## Max. :7.000 Max. :7.000 Max. :7.000 Max. :7.000
##
## F_BlurredVission F_DoubleVision F_WeightLoss F_WeightGain
## Min. :1.000 Min. :1.000 Min. :1.000 Min. :1.000
## 1st Qu.:2.000 1st Qu.:2.000 1st Qu.:4.000 1st Qu.:4.000
## Median :4.000 Median :4.000 Median :4.000 Median :4.500
## Mean :4.138 Mean :3.879 Mean :4.741 Mean :4.879
## 3rd Qu.:7.000 3rd Qu.:6.000 3rd Qu.:7.000 3rd Qu.:7.000
## Max. :7.000 Max. :7.000 Max. :7.000 Max. :7.000
##
## F_ChestPain F_Convulsion F_Anxiety F_Bleeding
## Min. :1.000 Min. :1.000 Min. :1.000 Min. :1.000
## 1st Qu.:2.000 1st Qu.:1.000 1st Qu.:4.000 1st Qu.:3.250
## Median :4.000 Median :3.000 Median :6.000 Median :4.500
## Mean :4.138 Mean :3.345 Mean :5.362 Mean :4.707
## 3rd Qu.:6.750 3rd Qu.:5.000 3rd Qu.:7.000 3rd Qu.:7.000
## Max. :7.000 Max. :7.000 Max. :7.000 Max. :7.000
##
## F_Depression F_Hallucination F_Insomnia F_Paranoia
## Min. :1.000 Min. :1.000 Min. :1.000 Min. :1.000
## 1st Qu.:4.000 1st Qu.:1.000 1st Qu.:4.000 1st Qu.:2.000
## Median :6.000 Median :4.000 Median :5.000 Median :4.000
## Mean :5.207 Mean :3.931 Mean :4.983 Mean :3.897
## 3rd Qu.:7.000 3rd Qu.:7.000 3rd Qu.:7.000 3rd Qu.:6.000
## Max. :7.000 Max. :7.000 Max. :7.000 Max. :7.000
##
## F_Tics F_Arrythmia Age Gender English
## Min. :1.000 Min. :1.000 Min. :19.00 Female :36 Yes :58
## 1st Qu.:1.000 1st Qu.:1.000 1st Qu.:27.25 Male :22
## Median :3.000 Median :4.000 Median :35.50
## Mean :3.552 Mean :3.397 Mean :38.10
## 3rd Qu.:5.750 3rd Qu.:5.000 3rd Qu.:48.75
## Max. :7.000 Max. :7.000 Max. :76.00
##
## X X.1
## Mode:logical Mode:logical
## NA's:58 NA's:58
##
##
##
##
##

# Descriptives for participants
length(pretest1$Answer) # Number of participants = 58

## [1] 58

mean(pretest1$Age) # Average age = 38.10

## [1] 38.10345

sd(pretest1$Age) # SD age = 14.10

## [1] 14.10075

range(pretest1$Age) # range age = 19-76

## [1] 19 76

table(pretest1$Gender) # Number of males (22) and females (36)

##
## Female Male
## 36 22

# Open Dataset with means and sds for severity and familiarity
sevfam<-read.csv("C://sevfammeansd.csv")
str(sevfam)

## 'data.frame': 55 obs. of 5 variables:
## $ X : Factor w/ 54 levels "AbdominalPain",..: 26 12 21 40 43 40 9 47 11 35 ...
## $ Severity_mean : num 2.83 2.93 4.66 4.19 4.02 ...
## $ Severity_sd : num 1.53 1.62 1.28 1.3 1.43 ...
## $ Familiarity_Mean: num 5.33 5.52 5.52 5.07 5.14 ...
## $ Familiarity_SD : num 1.67 1.78 1.55 1.75 1.81 ...

head(sevfam)

## X Severity_mean Severity_sd Familiarity_Mean Familiarity_SD
## 1 Irritability 2.827586 1.534834 5.327586 1.668994
## 2 Crying 2.931034 1.620722 5.517241 1.779428
## 3 Fever 4.655172 1.278043 5.517241 1.547396
## 4 Rash 4.189655 1.303933 5.068966 1.745793
## 5 Shivering 4.017241 1.432595 5.137931 1.810770
## 6 Rash 4.172414 1.352554 5.051724 1.781212

tail(sevfam)

## X Severity_mean Severity_sd Familiarity_Mean
## 50 Depression 4.517241 1.635584 5.206897
## 51 Hallucinations 5.362069 1.629748 3.931034
## 52 Insomnia 4.327586 1.604685 4.982759
## 53 Paranoia 5.086207 1.678392 3.896552
## 54 Tics 4.344828 1.550911 3.551724
## 55 Arrythmia 5.293103 1.486886 3.396552
## Familiarity_SD
## 50 2.049892
## 51 2.426911
## 52 1.914775
## 53 2.337262
## 54 2.264698
## 55 2.068474

summary(sevfam)

## X Severity_mean Severity_sd Familiarity_Mean
## Rash : 2 Min. :2.724 Min. :1.173 Min. :2.638
## AbdominalPain: 1 1st Qu.:3.767 1st Qu.:1.371 1st Qu.:4.138
## Anxiety : 1 Median :4.224 Median :1.436 Median :4.810
## Arrythmia : 1 Mean :4.400 Mean :1.452 Mean :4.696
## Bleeding : 1 3rd Qu.:5.121 3rd Qu.:1.538 3rd Qu.:5.241
## Blindness : 1 Max. :6.017 Max. :1.701 Max. :5.828
## (Other) :48
## Familiarity_SD
## Min. :1.429
## 1st Qu.:1.780
## Median :1.922
## Mean :1.950
## 3rd Qu.:2.122
## Max. :2.490
##

# Selection of Symptoms with Mean of Familiarity > 4.5 and SD of Severity >1.3
selection<-sevfam[ which(sevfam$Familiarity_Mean > 4.5 & sevfam$Severity_sd>1.3), ]
selection

## X Severity_mean Severity_sd Familiarity_Mean
## 1 Irritability 2.827586 1.534834 5.327586
## 2 Crying 2.931034 1.620722 5.517241
## 4 Rash 4.189655 1.303933 5.068966
## 5 Shivering 4.017241 1.432595 5.137931
## 6 Rash 4.172414 1.352554 5.051724
## 7 Chills 4.017241 1.317778 5.068966
## 8 Swelling 4.517241 1.392226 5.017241
## 9 Cough 3.672414 1.381430 5.810345
## 10 Nausea 4.103448 1.372533 5.534483
## 13 Vomiting 4.948276 1.479953 5.362069
## 14 Diarrhea 4.913793 1.478317 5.689655
## 15 Headache 4.051724 1.571929 5.568966
## 16 Dizziness 4.534483 1.391900 5.275862
## 18 Earache 3.948276 1.468051 4.706897
## 20 Wheezing 4.482759 1.477805 4.534483
## 21 AbdominalPain 4.844828 1.448553 4.913793
## 26 Redness 3.362069 1.307177 4.724138
## 27 Itching 3.327586 1.316400 5.155172
## 29 MuscleAche 3.637931 1.435127 5.275862
## 30 Lossofappetite 3.862069 1.503776 4.724138
## 31 Soreness 3.603448 1.413465 5.137931
## 32 Hoarseness 3.327586 1.431328 4.620690
## 33 ItchyEyes 3.241379 1.418058 4.982759
## 37 Toothache 4.189655 1.700948 4.827586
## 39 Light_Headed 4.137931 1.317204 4.534483
## 40 DryMouth 3.448276 1.378690 4.758621
## 44 Weightloss 4.224138 1.600911 4.741379
## 45 WeightGain 4.137931 1.515398 4.879310
## 48 Bleeding 5.465517 1.477498 4.706897
## 49 Anxiety 4.155172 1.694177 5.362069
## 50 Depression 4.517241 1.635584 5.206897
## 52 Insomnia 4.327586 1.604685 4.982759
## Familiarity_SD
## 1 1.668994
## 2 1.779428
## 4 1.745793
## 5 1.810770
## 6 1.781212
## 7 1.853040
## 8 1.896362
## 9 1.515498
## 10 1.708755
## 13 1.661729
## 14 1.429107
## 15 1.579608
## 16 1.745099
## 18 1.825825
## 20 2.096074
## 21 1.922028
## 26 1.861834
## 27 1.784944
## 29 1.775003
## 30 1.962751
## 31 1.820433
## 32 1.804746
## 33 1.933013
## 37 1.965830
## 39 2.053799
## 40 1.894686
## 44 2.039611
## 45 1.956344
## 48 2.111029
## 49 1.916670
## 50 2.049892
## 52 1.914775

length(selection$Familiarity_Mean) # 31 symtpoms met the requirements (32 - 1 because Rash was repeted)

## [1] 32

sort(selection$X)

## [1] AbdominalPain Anxiety Bleeding Chills
## [5] Cough Crying Depression Diarrhea
## [9] Dizziness DryMouth Earache Headache
## [13] Hoarseness Insomnia Irritability Itching
## [17] ItchyEyes Light_Headed Lossofappetite MuscleAche
## [21] Nausea Rash Rash Redness
## [25] Shivering Soreness Swelling Toothache
## [29] Vomiting WeightGain Weightloss Wheezing
## 54 Levels: AbdominalPain Anxiety Arrythmia Bleeding ... Wheezing

# Selection of Symptoms with Mean of Familiarity > 4.5 and SD of Severity > 1.2
selection2<-sevfam[ which(sevfam$Familiarity_Mean >4.5 & sevfam$Severity_sd>1.2 & sevfam$Severity_sd<1.3), ]
selection2

## X Severity_mean Severity_sd Familiarity_Mean
## 3 Fever 4.655172 1.278043 5.517241
## 11 Sneezing 2.724138 1.294971 5.827586
## 19 JointPain 4.034483 1.297305 4.810345
## 34 NasalCongestion 3.258621 1.291814 5.465517
## Familiarity_SD
## 3 1.547396
## 11 1.601950
## 19 1.959743
## 34 1.569619

length(selection2$Familiarity_Mean) # 31 symptoms met the requirements (32 - 1 because Rash was repeted)

## [1] 4

sort(selection2$X)

## [1] Fever JointPain NasalCongestion Sneezing
## 54 Levels: AbdominalPain Anxiety Arrythmia Bleeding ... Wheezing

# C: PRETEST 2

## 1.- INTRODUCTION

The goal of Pre-test 2 was to check the effects of the experimental manipulations. In section 1.1. of the article, it was suggested that a possible explanation for the omission bias was that bad outcomes resulting from commissions might entail a higher level of regret than the similar (or worse) outcomes resulting from omissions (Asch et al., 1994; Spranca, Minsk, & Baron, 1991). Another possibility is that the level of regret due to bad outcomes resulting from vaccine decisions (either vaccinate or not to vaccinate) does not depend on whether the bad outcomes derived from either an omission or a commission but on people’s attitudes towards vaccination. In this sense, people with positive views of vaccination might consider less regrettable vaccine side effects (commissions) than the symptoms of a vaccine-preventable disease (omissions). In contrast, people with negative views of vaccination might consider that the symptoms of a vaccine-preventable disease (omissions) are less regrettable than the vaccine side effects (commissions). Support for this hypothesis was found in an experiment conducted by Connolly and Reb (2003), which showed that participants’ level of regret resulting from vaccine decisions was congruent with their attitudes towards vaccines. In order to check whether the experimental condition (omission/commission) or its interaction with participants attitudes towards vaccination differentially elicit feelings of regret, participants in this pre-test were asked to rate the level of regret that the assigned experimental condition elicited and to self-report their attitudes towards vaccination.

In addition, the elicitation of disgust due to contact with potential contaminants through vaccination (Miton & Mercier, 2015) as well as a greater disgust sensibility (Clifford & Wendell, 2016) have been recently linked to anti-vaccination attitudes. However, the evidence for this is not very convincing as fears towards non-disgusting perceived fears (e.g. children drowning in swimming pools) have stronger associations with disgust sensibility than negative beliefs on vaccination (Kahan & Hilgard, 2016). Nevertheless, it might have been an important factor in the spread of anti-vaccination attitudes in the early history of vaccination (see Section 1). More importantly, there is substantial evidence that the elicitation of disgust (Eriksson & Coultas, 2014; Heath, Bell, & Sternberg, 2001) and other emotions (Stubbersfield, Tehrani, & Flynn, 2017) increases the transmissibility and memorability of stories. As a consequence, it was necessary to check whether the two processing experimental conditions differ in the elicitation of disgust and other emotions and whether people with greater anti-vaccination attitudes feel more disgusted by a scenario in which they had to imagine that their 1 year old child suffered from the side effects of one vaccine than when they had to imagine that the same child suffered from the symptoms of a vaccine-preventable infectious disease.

Similarly, in order to rule out certain alternative explanations for the results of the experiment, it was required to check beforehand if the instructions for the different processing conditions were equally easy to read and imagine as well as to be considered equally plausible by the participants.

## 2.- METHOD

### 2.1.- Participants

65 participants (32 male, 32 female, 1 other) aged 19-58 (*M*=35.43, *SD*=11.59) completed the study. All the participants were English native speakers with British (*N*=47) or American (*N*=18) nationality. The participants were recruited online through Prolific (Peer, Samat, Brandimarte, & Acquisti, 2016). Using pre-screening filters, participants who had not previously participated in any of our vaccine-related experiments, had an approval rate of 90% or above, were aged from 18 to 60 years old, spoke English as a first language and had British or American nationality were selected to participate in the study. These characteristics were assumed to increase the chances of collecting responses provided by participants who understood the content of the information and paid attention to the materials. In addition, participants were pre-screened by their gender and attitudes towards vaccination in order to ensure a balanced distribution of genders (50% males, 50% females) and attitudes towards vaccination (50% pro-vaccination, 50% anti-vaccination). For pre-screening vaccination attitudes, participants indicated their agreement with the following item on a scale from 1 (totally disagree) to 7 (totally agree): “*If I had a baby to care for, I would want him/her to get all the recommended immunizations*” (Browne, Thomson, Rockloff, & Pennycook, 2015). Participants who agreed (ratings from 5 to 7) were considered to have pro-vaccination attitudes and participants who disagreed (ratings from 1 to 3) were considered to have anti-vaccination attitudes.

To ensure that participants included in the analyses were reading carefully the experimental materials and paying attention to the questions, two procedures were employed. The first procedure was the inclusion of an attentional check item (“If you are carefully reading the questions, select ‘2’” within a 7-point Likert scale). One participant failed to answer correctly this attentional check and, therefore, the participant was removed from the final dataset. The second procedure involved measuring the time spent by the participants reading the text that contained the experimental manipulations. Following a procedure previously used by Boyer and Parren (2015), 12 participants who read the text at a greater pace than 400 words per minute were removed from the dataset. The final dataset was composed by the remaining 53 participants (28 males, 24 females and 1 other; 39 British and 14 Americans), which ages ranged between 20 and 58 (*M*=36.26, *SD*=11.11).

## 2.2.- Materials

An online questionnaire was created and applied with Qualtrics. Two different texts were created as processing conditions. The texts were adapted from Connolly and Reb (2003), who themselves adapted the scenarios from Ritov and Baron (1990) and Asch et al. (1994). The greatest part of the information was similar across the two texts. The texts varied in only one aspect: one text prompted the participants to imagine that they had a one-year-old child who suffered from a set of symptoms as a consequence of a vaccine-preventable infectious disease (*Omission Condition*), while the other text prompted the participants to imagine that the symptoms were the consequence of the vaccine side effects (*Commission Condition*). See Figure 1.

Figure 1. Text for the Processing Conditions.

*Imagine that, in the country you live in, there had been several outbreaks of a certain new infectious disease, which can cause severe illness to children under three. Only a small number of children exposed actually catch this disease, but for those who do it is often quite severe.*

*A vaccine for this disease has been developed and tested.* The *vaccine eliminates any possibility of the child getting the disease. The vaccine, however, can sometimes cause side-effects that are very similar to the symptoms of the disease. Fortunately, these unpleasant effects are rare. In fact, the risk of a vaccinated child getting the unpleasant side-effects is about as low as the chance of a non-vaccinated child getting the severe symptoms of the disease.*

*Imagine that you have one child, a one-year old.* ***Suppose you did decide to vaccinate. Unfortunately, your child is one of those who suffer from the vaccine side-effects*** [Commission Condition] // **Suppose you decided not to vaccinate. Unfortunately, your child is one of those who suffer from the disease** [Omission Condition].

NOTE: Sentences in bold face indicates sentences that were different across the conditions.

3 items were included in the questionnaire to explore the quality of the texts. These items prompted participants to rate on a 7-point Likert scale how easy to read and to understand the texts were as well as how easy to imagine and how plausible the described situations were. In addition, participants were asked to rate the elicitation of the following emotions by the texts on a 7 point Likert scale (1=very little, 7=very much): fear, anger, sadness, joy, compassion, disgust, surprise, confusion and regret. Regret was the emotion that was considered relevant for testing the hypotheses stated in the introduction. Disgust was also considered of interest due to both their disputed role in the formation of anti-vaccination beliefs (Clifford & Wendell, 2016; Kahan & Hilgard, 2016; Miton & Mercier, 2015) and the greater transmissibility of disgust-eliciting content in American samples (Eriksson & Coultas, 2014; Eriksson, Coultas, & de Barra, 2016; Heath et al., 2001). The other emotions were included to make the researcher’s hypothesis less salient and to control their possible effects.

Two items were included to measure attitudes towards vaccination (“*If I had a baby to care for, I would want him/her to get all the recommended immunizations*” and “*I believe that scheduled immunizations are safe for children*”). These items were previously used by Browne et al. (2015), who adapted the items themselves from Gust et al. (2004). A 7-point Likert scale was used to collect the participants’ responses (1=totally disagree, 7=totally agree) on these three items. The scores of the two items about vaccination attitudes were summed together, divided by two and converted into a scale from -3 to +3.

## 2.3.- Design and Procedure

A between-subjects design was used for this pre-test. When participants clicked on the link to the questionnaire, they were randomly assigned to one of the 2 conditions (Commission Condition or Omission Condition). With the exception of the text participants read, the questionnaire was identical for all the subjects.

## 3- RESULTS

### 3.1.- Attitudes towards Vaccination

Although participants were pre-screened to ensure a balanced distribution of pro-vaccination and anti-vaccination attitudes, the sample was considerably skewed towards pro-vaccination attitudes in both conditions (Commission: *M*=1.3, *SD*=2; Omission: *M*=1.7, SD=1.55). Nevertheless, an ordinal logistic regression model with Condition as a predictor of vaccination attitudes (AIC=238.22) did not improve the fit of the null model (AIC=236.34). In contrast, a model with gender as unique predictor (AIC=233.95) improve the model fit of the null model (236.44). Women had a less positive view about vaccines than men (Women: *M*=0.88, *SD*=2.08; Men: *M*=2.05, *SD*=1.34; *β*=-1.07, *SE*=0.52). The differences between men and women in vaccination attitudes might be explained by the fact that women who provided an anti-vaccination response to the pre-screening question were more likely to maintain an anti-vaccination attitude in the experiment (*M*=-0.875, *SD*=1.43), while the responses of men who reported an anti-vaccination attitude in the pre-screening tended to provide a pro-vaccination response during the experiment (*M*=1.5, *SD*=1.66).

### 3.3.2- Quality of the experimental materials

The materials were considered very easy to read and understand (*M*=6.06, *SD*=1.35), very easy to imagine (*M*=5.94, *SD*=1.31) and the situations were regarded as plausible (*M*=5.26, *SD*=1.53) by the participants. Model comparisons show that there were no differences between conditions or attitudes towards vaccines in rating the quality of the materials.

### 3.3.3.- Emotions

The ratings did not support the hypothesis that the bad outcomes due to commissions

(*M*=2.72, *SD*=2.07) implied a greater level of regret than bad outcomes due to omissions (*M*=2.71, *SD*=1.64, AIC_null_=194.41, AIC_Condition_=195.30). Moreover, attitudes towards vaccines did not affect the level of regret elicited by the experimental conditions (AIC_Condition * Attitudes_ = 198.5815).

Similarly, the ratings of disgust did not support the hypothesis that the bad outcomes due to commissions (*M*=4.76, *SD*=1.42) elicit a greater level of disgust that the bad outcomes due to omissions (*M*=4.25, *SD*=1.67, AIC_null_=199.30, AIC_Condition_=199.61). Moreover, attitudes towards vaccines did not affect the level of disgust elicited by the experimental conditions (AIC_Condition * Attitudes_ = 202.75).

The ratings of the other emotions included in the study (fear, anger, sadness, joy, compassion, surprise and confusion) did not differ across conditions and attitudes towards vaccines did not affect the level of any of these emotions were elicited by the experimental conditions.

## 4.- DISCUSSION

The scenarios obtained high ratings in “easy to read and understand”, “easy to imagine” and “plausibility”. Therefore, they seem to be adequate for the experiment. More importantly, the high ratings in these variables did not differ across conditions and attitudes towards vaccination do not affect the ratings, which were requirements for using this imagined scenarios for the experiment.

In addition, the experimental conditions did not differ in the level of regret, disgust or other emotions they elicited. Therefore, if the expected differences in recall across conditions take place in the experiment, they could not possibly be explained by differences in the level of emotionality of the experimental conditions. Similarly, vaccination attitudes were not associated with a higher level of disgust or regret in any of the imagined scenarios. As a consequence, if vaccination attitudes affect the recall and the ratings of severity of the symptoms/side effects in the experiment, these differences could not be explained by differences in the level of regret and disgust than the scenarios elicit in people with different attitudes towards vaccines.

A limitation of this pre-test is the lack of congruency between the reported attitudes towards vaccination in the pre-screening and in the experiment, above all, for male participants. A possible explanation for this lack of congruency is that some participants had inferred that reporting anti-vaccination attitudes would ensure their participation in a greater number of studies and cause them to earn more money. In a set of studies, Chandler and Paolacci (2017) have shown that participants can lie by self-reporting rare conditions (e.g. having a child with autism) in order to have access to more studies. As in the present pretest, men showed a greater fraudulent tendency than women (Chandler & Paolacci, 2017, Study 3). This could be very problematic as it entails threats to the validity of the studies. In order to overcome this difficulty, it was decided to include only women in the experiment, as their pre-screened anti-vaccination attitudes seem to be more reliable than the ones reported by men. This comes with the obvious cost that we can only make inferences about women’s ratings and recall, and not men’s. Similarly to the pre-test, it was decided to recruit half of participants with self-reported pro-vaccination beliefs and half of the participants with self-reported anti-vaccination beliefs in the pre-screening. In the analyses, however, self-reported vaccination beliefs were decided to be used as predictor variables of severity ratings and recall. This decision was taken for two reasons. First, the exact ratings for the pre-screening question to measure vaccination beliefs is not available with the currently implemented procedure on Prolific. Second, we assume that participants’ self-reported attitudes towards vaccination would be more honest in the experiment than in the pre-screening. The rationale for this assumption is that, in contrast to misreporting anti-vaccination attitudes in the pre-screening, doing so in the experiment does not have any monetary benefit.

# D: R SCRIPT FOR PRETEST 2

**Pretest2**

Angel V. Jimenez

13 April 2018

*#Open data file*
d<-**read.csv**("C://files/Angel/omission/pretest2/pretest2.csv")
**str**(d)

## 'data.frame': 65 obs. of 32 variables:
## $ StartDate : Factor w/ 49 levels "10/04/2017 14:31",..: 1 1 3 2 4 5 5 6 6 6 ...
## $ EndDate : Factor w/ 54 levels "10/04/2017 14:32",..: 1 2 3 4 4 5 5 6 6 7 ...
## $ Pre_Vac_Attitudes : int 1 1 1 1 1 1 1 1 1 1 ...
## $ CONDITION : int 2 1 2 2 1 1 2 2 2 1 ...
## $ Duration__in_seconds_: int 109 234 107 190 105 111 155 110 157 182 ...
## $ RecordedDate : Factor w/ 54 levels "10/04/2017 14:32",..: 1 2 3 4 4 5 5 6 6 7 ...
## $ Time_Reading : num 28.9 98.3 22.1 65.6 35.6 ...
## $ EasyRead : int 7 7 7 7 6 1 7 5 3 6 ...
## $ EasyImagine : int 7 7 7 6 6 1 6 5 3 6 ...
## $ Plausibility : int 6 7 5 4 2 7 6 2 6 6 ...
## $ Fear : int 6 7 5 4 2 7 6 2 6 6 ...
## $ Anger : int 2 2 2 1 5 7 3 7 1 1 ...
## $ Sadness : int 1 1 6 1 2 7 1 5 1 1 ...
## $ Joy : int 2 4 5 1 2 7 3 7 1 1 ...
## $ Compassion : int 1 1 3 1 1 7 3 1 1 1 ...
## $ Disgust : int 5 4 5 3 4 7 2 5 4 1 ...
## $ Surprise : int 1 1 4 1 1 7 1 5 1 1 ...
## $ Regret : int 1 1 3 2 1 7 1 6 1 1 ...
## $ Confussion : int 1 1 3 1 1 7 1 7 1 1 ...
## $ Time_answering : num 27.2 52.7 34.8 43.8 31.2 ...
## $ CAM : int 1 1 2 1 1 1 2 2 2 2 ...
## $ Provac1 : int 7 6 7 7 7 7 7 7 7 7 ...
## $ Attention_Check : int 2 2 2 2 2 2 2 2 2 2 ...
## $ Provac2 : int 7 5 7 7 7 7 7 6 5 6 ...
## $ Time_answeringVAC : num 12 43 24.7 34.6 17 ...
## $ Age : int 4 4 3 15 13 24 31 5 31 36 ...
## $ Gender : int 1 1 1 1 1 1 1 1 1 1 ...
## $ Nationality : int 1 1 1 2 1 1 1 2 1 1 ...
## $ English : int 1 1 1 1 1 1 1 1 1 1 ...
## $ Time_Demographics : num 7.4 7.22 8.83 11.08 7.16 ...
## $ AGE_REAL : int 22 22 21 33 31 42 49 23 49 54 ...
## $ Vac_scale : int 14 11 14 14 14 14 14 13 12 13 ...

**summary**(d)

## StartDate EndDate Pre_Vac_Attitudes
## 10/04/2017 14:41: 5 10/04/2017 14:44: 4 Min. :0.0000
## 10/04/2017 15:23: 4 10/04/2017 15:26: 3 1st Qu.:0.0000
## 10/04/2017 15:27: 4 10/04/2017 15:29: 3 Median :1.0000
## 10/04/2017 14:31: 2 10/04/2017 14:38: 2 Mean :0.5077
## 10/04/2017 14:35: 2 10/04/2017 14:40: 2 3rd Qu.:1.0000
## 10/04/2017 14:38: 2 10/04/2017 14:43: 2 Max. :1.0000
## (Other) :46 (Other) :49
## CONDITION Duration__in_seconds_ RecordedDate
## Min. :1.000 Min. : 83.0 10/04/2017 14:44: 4
## 1st Qu.:1.000 1st Qu.: 112.0 10/04/2017 15:26: 3
## Median :2.000 Median : 155.0 10/04/2017 15:29: 3
## Mean :1.523 Mean : 213.9 10/04/2017 14:38: 2
## 3rd Qu.:2.000 3rd Qu.: 192.0 10/04/2017 14:40: 2
## Max. :2.000 Max. :1335.0 10/04/2017 14:43: 2
## (Other) :49
## Time_Reading EasyRead EasyImagine
## 1 Min. : 4.62 Min. :1.000 Min. :1.000
## 1 1st Qu.: 26.67 1st Qu.:6.000 1st Qu.:6.000
## 1 Median : 36.20 Median :7.000 Median :6.000
##: 1 Mean : 71.69 Mean :6.077 Mean :5.969
## 3rd Qu.: 65.70 3rd Qu.:7.000 3rd Qu.:7.000
## Max. :587.74 Max. :7.000 Max. :7.000
## (Other) :59
## Plausibility Fear Anger Sadness
## Min. :1.000 Min. :1.000 Min. :1.000 Min. :1.000
## 1st Qu.:4.000 1st Qu.:4.000 1st Qu.:2.000 1st Qu.:1.000
## Median :5.000 Median :5.000 Median :4.000 Median :3.000
## Mean :5.092 Mean :5.092 Mean :3.923 Mean :3.215
## 3rd Qu.:6.000 3rd Qu.:6.000 3rd Qu.:5.000 3rd Qu.:5.000
## Max. :7.000 Max. :7.000 Max. :7.000 Max. :7.000
##
## Joy Compassion Disgust Surprise
## Min. :1.000 Min. :1.000 Min. :1.000 Min. :1.000
## 1st Qu.:3.000 1st Qu.:1.000 1st Qu.:4.000 1st Qu.:1.000
## Median :5.000 Median :1.000 Median :5.000 Median :2.000
## Mean :4.246 Mean :1.646 Mean :4.415 Mean :2.477
## 3rd Qu.:6.000 3rd Qu.:2.000 3rd Qu.:5.000 3rd Qu.:4.000
## Max. :7.000 Max. :7.000 Max. :7.000 Max. :7.000
##
## Regret Confussion Time_answering CAM
## Min. :1.000 Min. :1.000 Min. : 16.38 Min. :1.000
## 1st Qu.:1.000 1st Qu.:1.000 1st Qu.: 30.85 1st Qu.:1.000
## Median :3.000 Median :3.000 Median : 37.07 Median :2.000
## Mean :2.985 Mean :3.369 Mean : 42.49 Mean :3.077
## 3rd Qu.:4.000 3rd Qu.:5.000 3rd Qu.: 49.53 3rd Qu.:5.000
## Max. :7.000 Max. :7.000 Max. :132.58 Max. :7.000
##
## Provac1 Attention_Check Provac2 Time_answeringVAC
## Min. :1.000 Min. :2.000 Min. :1.000 Min. : 5.55
## 1st Qu.:5.000 1st Qu.:2.000 1st Qu.:4.000 1st Qu.: 15.91
## Median :7.000 Median :2.000 Median :6.000 Median : 22.08
## Mean :5.723 Mean :2.031 Mean :5.277 Mean : 25.17
## 3rd Qu.:7.000 3rd Qu.:2.000 3rd Qu.:7.000 3rd Qu.: 28.58
## Max. :7.000 Max. :4.000 Max. :7.000 Max. :113.98
##
## Age Gender Nationality English
## Min. : 1.00 Min. :1.000 Min. :1.000 Min. :1.000
## 1st Qu.: 8.00 1st Qu.:1.000 1st Qu.:1.000 1st Qu.:1.000
## Median :15.00 Median :2.000 Median :1.000 Median :1.000
## Mean :17.43 Mean :1.523 Mean :1.277 Mean :1.015
## 3rd Qu.:28.00 3rd Qu.:2.000 3rd Qu.:2.000 3rd Qu.:1.000
## Max. :40.00 Max. :3.000 Max. :2.000 Max. :2.000
##
## Time_Demographics AGE_REAL Vac_scale
## Min. : 4.848 Min. :19.00 Min. : 2
## 1st Qu.: 8.839 1st Qu.:26.00 1st Qu.: 9
## Median :11.078 Median :33.00 Median :13
## Mean :12.973 Mean :35.43 Mean :11
## 3rd Qu.:15.689 3rd Qu.:46.00 3rd Qu.:14
## Max. :47.498 Max. :58.00 Max. :14
##

**LIBRARIES**

*# Open libraries*
**library**(plyr)

## Warning: package 'plyr' was built under R version 3.4.4

**library**(lattice)

**WHOLE SAMPLE OF PARTICIPANTS (DESCRIPTIVES)**

*# Gender (Frequencies)*
**summary**(**as.factor**(d$Gender))*# 1 = men, 2 = female, 3 = other. Results = 32 men, 32 female, 1 other.*

## 1 2 3
## 32 32 1

*# Nationalities (Frequencies)*
**summary**(**as.factor**(d$Nationality)) *# 1 = British, 2 = American. Results = 47 British and 18 American.*

## 1 2
## 47 18

*# Age (Range)*
**range**(d$AGE_REAL)*# Only 18 years old and older were allowed to participate in the study.*

## [1] 19 58

*# A problem with the software made necessary to sum 18 to the ages provided by the software. Results = 19-58*
*# Age (Mean)*
**mean**(d$AGE_REAL) *# M = 35.43*

## [1] 35.43077

*# Age (standard Deviation)*
**sd**(d$AGE_REAL) *# 11.59*

## [1] 11.58659

*# Histogram of Age*
breaks<-**c**(15, 20, 25, 30, 35, 40, 45, 50, 55, 60)
**hist**(d$AGE_REAL,
 main="Histogram for Age",
 xlab="Age",
 border="blue",
 col="green",
 breaks = breaks,
 xlim=**c**(15,60),
 ylim=**c**(0,15),
 prob = FALSE,
 xaxt ="n")
**axis**(side=1, at=**seq**(15,60,5), labels=**seq**(15,60,5))


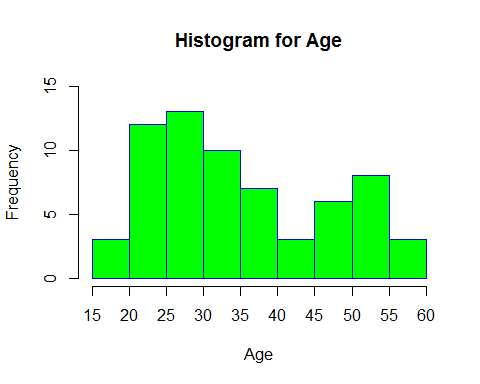


**QUALITY OF RESPONSES**

*# Attentional Check (Frequencies) --> 2 = correct; 1, 3, 4, 5, 6, 7 incorrect. It was identified one participant*
*# who failed the attentional check*
**summary**(**as.factor**(d$Attention_Check)) *# 64 correct responses, 1 incorrect response.*

## 2 4
## 64 1

*# Creation of new dataset excluding the participant who failed the attentional check*
da <- d[ **which**(d$Attention_Check=='2'),]
*# Checking whether one observation has been deleted from the dataset in the new dataset.*
**library**(plyr)
**nrow**(d) *# N = 65 (initial dataset)*

## [1] 65

**nrow**(da) *# N = 64 (new dataset)*

## [1] 64

*# Time Reading the processing materials*
*# Duration (seconds) both conditions together*
**range**(da$Time_Reading) *# 5 - 588 secons*

## [1] 4.62 587.74

**mean**(da$Time_Reading) *# M = 73*

## [1] 72.64312

**sd**(da$Time_Reading) *# SD = 113*

## [1] 112.8702

*#Duration (Seconds) Condition1*
C1<-d[**which**(d$CONDITION =="1" & d$Attention_Check=="2"),] *# only participants who did not fail manipulation check*
**nrow**(C1) *# N = 31*

## [1] 31

**range**(C1$Time_Reading) *# range: 5 - 588*

## [1] 4.62 587.74

**mean**(C1$Time_Reading) *# M = 97.33*

## [1] 97.33065

**sd**(C1$Time_Reading) *# SD = 149*

## [1] 148.6047

*#Duration (seconds) Condition 2*
C2<-d[**which**(d$CONDITION =="2"& d$Attention_Check=='2'),] *# only participants who did not fail manipulation check*
**nrow**(C2) *# N = 33*

## [1] 33

**range**(C2$Time_Reading) *# Range = 5 - 331 seconds*

## [1] 5.46 331.10

**mean**(C2$Time_Reading) *# M = 49*

## [1] 49.45182

**sd**(C2$Time_Reading) *# SD = 57*

## [1] 56.85969

*# Number of Words: Condition 1 = 152 words, Condition 2 = 151 words.*
*# Estimated Time to Read = 400 words per minute or slower => 23 seconds to read the whole text or slower*
151.5*60/400

## [1] 22.725

*#Creation of New Data Eliminating Participants who "read" the processing conditions too fast (<23 seconds)*
das<-d[ **which**(d$Attention_Check=='2'& d$Time_Reading>=23),]
**nrow**(das) *# 53 Participants remained*

## [1] 53

**nrow**(d)-**nrow**(das) *# 12 Participants were eliminated due to their fast reading*

## [1] 12

**nrow**(da)-**nrow**(das) *# The individual who did not answer correctly the attentional check did not read at a pace*

## [1] 11

*# greater than 400 words per minute*

*# Creation of Separate Datasets for each condition*
C1s<-d[**which**(d$CONDITION =="1" & d$Attention_Check=="2"& d$Time_Reading>=23),]
**nrow**(C1s)*# 28 Participants in Condition 1*

## [1] 28

*# Creation of Separate Datasets for each condition*
C2s<-d[**which**(d$CONDITION =="2" & d$Attention_Check=="2"& d$Time_Reading>=23),]
**nrow**(C2s) *# 25 participants in Condition 2*

## [1] 25

*#New descriptive statistics for participants in new dataset (participants who did not fail the attentional check*
*# and who did not read the instructions too fast)*
*# Gender (Frequencies)*
**summary**(**as.factor**(das$Gender))

## 1 2 3
## 28 24 1

*# 1 = men, 2 = female, 3 = other. Results = 28 men, 24 female, 1 other.*
 *# 28 men, 24 women, 1 other*
*# Nationalities (Frequencies)*
**summary**(**as.factor**(das$Nationality)) *# 1 = British, 2 = American. Results = 39 British and 14 American.*

## 1 2
## 39 14

*# Age (Range) # Only 18 years old and older were allowed to participate in the study.*
*# A problem with the software made need to sum 18 to the ages provided by the software. Results = 19-58*
**range**(das$AGE_REAL)*# 20-58 years old*

## [1] 20 58

*# Age (Mean)*
**mean**(das$AGE_REAL)*# 36.26*

## [1] 36.26415

*# Age (standard Deviation)*
**sd**(das$AGE_REAL)*# 11.11*

## [1] 11.10639

**ATTITUDES TOWARDS VACCINATION**

*# Vaccination Attitudes*
*# Rescalate from +1 to +7 to -3 to +3 and divided by the 2 items*
das$Vac<-((das$Vac_scale)/2)-4

*# Histogram of Vaccination Attitudes (Both Conditions Together)*
**hist**(das$Vac,
 main="Attitudes Towards Vaccines",
 xlab="Vaccine Attitudes",
 ylab="Frequency",
 border="blue",
 col="green",
 prob = FALSE)


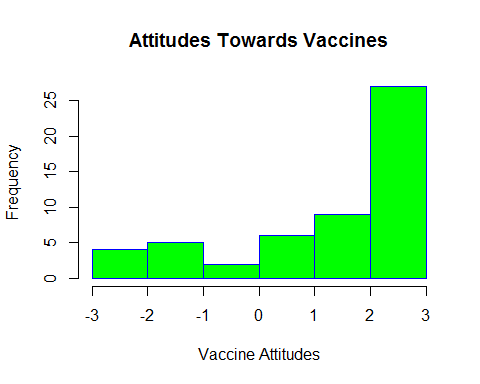


*# Summaries of attitudes towards vaccines for the entire sample*
**library**(plyr)
**library**(psych)
**describe**(das$Vac)

## vars n mean sd median trimmed mad min max range skew kurtosis se
## X1 1 53 1.49 1.79 2.5 1.76 0.74 -3 3 6 -1.02 -0.24 0.25

*# Histogram of attitudes towards vaccines splited by condition*
**library**(lattice)
**histogram**(~ Vac | CONDITION, main= "Attitudes Towards Vaccines", xlab="Score in the Scale", ylab="Frequency", col="green", border="blue", data=das)


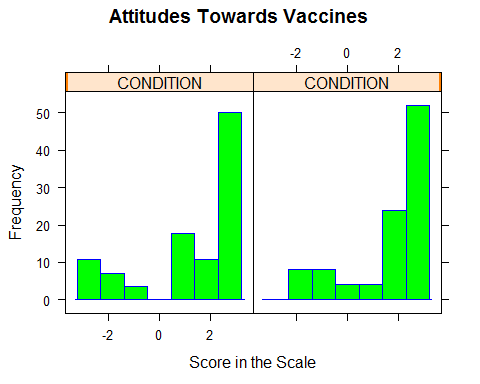


*# Summaries of attitudes towards vaccines per condition*
**library**(psych)
**describeBy**(das$Vac, das$CONDITION)

##
## Descriptive statistics by group
## group: 1
## vars n mean sd median trimmed mad min max range skew kurtosis se
## X1 1 28 1.3 2 2 1.5 1.48 -3 3 6 -0.88 -0.64 0.38
## --------------------------------------------------------
## group: 2
## vars n mean sd median trimmed mad min max range skew kurtosis
## X1 1 25 1.7 1.55 2.5 1.88 0.74 -1.5 3 4.5 -0.97 -0.53
## se
## X1 0.31

Interpretation: Although we used a pre-screening question to ensure a balanced distribution of attitudes towards vaccines, most of participants in the study showed positive attitudes towards vaccines. (Did some of them lie in the pre-screening questions?)

*# Removing "other" as gender*
dass<-das[ **which**(das$Gender<3),]
*# Intercept only ordinal logistic model*
**library**(MASS)
interceptonly<-**polr**(**as.ordered**(Vac)~1, data=dass, Hess=TRUE)
*# Condition ordinal logistic model*
conditionmodel<-**polr**(**as.ordered**(Vac)~CONDITION,data=dass, Hess=TRUE)
**summary**(conditionmodel)

## Call:
## polr(formula = as.ordered(Vac) ~ CONDITION, data = dass, Hess = TRUE)
##
## Coefficients:
## Value Std. Error t value
## CONDITION 0.1714 0.4977 0.3443
##
## Intercepts:
## Value Std. Error t value
## -3|-2.5 -3.6801 1.2444 -2.9573
## -2.5|-2 -2.5381 0.9474 -2.6790
## -2|-1.5 -2.2279 0.9070 -2.4563
## -1.5|-1 -1.6011 0.8539 -1.8751
## -1|-0.5 -1.3047 0.8351 -1.5623
## -0.5|0 -1.1761 0.8281 -1.4202
## 0|0.5 -1.0569 0.8224 -1.2851
## 0.5|1 -0.7402 0.8108 -0.9129
## 1|1.5 -0.5519 0.8083 -0.6828
## 1.5|2 0.0304 0.8102 0.0376
## 2|2.5 0.1854 0.8103 0.2289
## 2.5|3 0.7307 0.8091 0.9032
##
## Residual Deviance: 212.2222
## AIC: 238.2222

*# Gender ordinal logistic model*
gendermodel<-**polr**(**as.ordered**(Vac)~Gender,data=dass, Hess=TRUE)
*# Gender + Condition ordinal logistic model*
genderconditionmodel<-**polr**(**as.ordered**(Vac)~Gender+CONDITION,data=dass, Hess=TRUE)
*# Gender * Condition ordinal logistic model*
genderxconditionmodel<-**polr**(**as.ordered**(Vac)~Gender*CONDITION,data=dass, Hess=TRUE)
**AIC**(interceptonly, conditionmodel, gendermodel, genderconditionmodel, genderxconditionmodel)

## df AIC
## interceptonly 12 236.3409
## conditionmodel 13 238.2222
## gendermodel 13 233.9510
## genderconditionmodel 14 235.7206
## genderxconditionmodel 15 237.7163

**summary**(gendermodel)

## Call:
## polr(formula = as.ordered(Vac) ~ Gender, data = dass, Hess = TRUE)
##
## Coefficients:
## Value Std. Error t value
## Gender -1.069 0.5168 -2.069
##
## Intercepts:
## Value Std. Error t value
## -3|-2.5 -5.6234 1.3164 -4.2719
## -2.5|-2 -4.4759 1.0284 -4.3525
## -2|-1.5 -4.1630 0.9848 -4.2274
## -1.5|-1 -3.5098 0.9149 -3.8362
## -1|-0.5 -3.1867 0.8844 -3.6033
## -0.5|0 -3.0459 0.8721 -3.4926
## 0|0.5 -2.9187 0.8629 -3.3824
## 0.5|1 -2.5812 0.8410 -3.0692
## 1|1.5 -2.3792 0.8283 -2.8725
## 1.5|2 -1.7572 0.7950 -2.2104
## 2|2.5 -1.5946 0.7895 -2.0197
## 2.5|3 -1.0285 0.7794 -1.3196
##
## Residual Deviance: 207.951
## AIC: 233.951

**describeBy**(dass$Vac,dass$Gender)

##
## Descriptive statistics by group
## group: 1
## vars n mean sd median trimmed mad min max range skew kurtosis
## X1 1 28 2.05 1.34 2.5 2.27 0.74 -2.5 3 5.5 -1.68 2.59
## se
## X1 0.25
## --------------------------------------------------------
## group: 2
## vars n mean sd median trimmed mad min max range skew kurtosis se
## X1 1 24 0.88 2.08 1.5 1.02 2.22 -3 3 6 -0.43 -1.43 0.42

women_anti<-dass[ **which**(dass$Gender=="2" & dass$Pre_Vac_Attitudes=="0"),]
**mean**(women_anti$Vac)

## [1] -0.875

**sd**(women_anti$Vac)

## [1] 1.432179

women_pro<-dass[ **which**(dass$Gender=="2" & dass$Pre_Vac_Attitudes=="1"),]
**mean**(women_pro$Vac)

## [1] 2.625

**sd**(women_pro$Vac)

## [1] 0.5690902

men_anti<-dass[ **which**(dass$Gender=="1" & dass$Pre_Vac_Attitudes=="0"),]
**mean**(men_anti$Vac)

## [1] 1.5

**sd**(men_anti$Vac)

## [1] 1.664101

men_pro<-dass[ **which**(dass$Gender=="1" & dass$Pre_Vac_Attitudes=="1"),]
**mean**(men_pro$Vac)

## [1] 2.607143

**sd**(men_pro$Vac)

## [1] 0.5608569

**QUALITY OF THE EXPERIMENTAL MATERIALS**

**library**(psych)
**describe**(das$EasyRead)

## vars n mean sd median trimmed mad min max range skew kurtosis se
## X1 1 53 6.06 1.35 6 6.35 1.48 1 7 6 -1.89 3.5 0.19

**describeBy**(das$EasyRead, das$CONDITION)

##
## Descriptive statistics by group
## group: 1
## vars n mean sd median trimmed mad min max range skew kurtosis se
## X1 1 28 5.93 1.51 6 6.21 1.48 1 7 6 -1.8 2.87 0.29
## --------------------------------------------------------
## group: 2
## vars n mean sd median trimmed mad min max range skew kurtosis se
## X1 1 25 6.2 1.15 7 6.43 0 3 7 4 -1.62 1.93 0.23

null.EasyRead<-**polr**(**as.ordered**(EasyRead)~1,data=das, Hess=TRUE)
Condition.EasyRead<-**polr**(**as.ordered**(EasyRead)~**as.factor**(CONDITION),data=das, Hess=TRUE)
Vac.EasyRead<-**polr**(**as.ordered**(EasyRead)~Vac,data=das, Hess=TRUE)
VacCondition.EasyRead<-**polr**(**as.ordered**(EasyRead)~Vac+**as.factor**(CONDITION),data=das, Hess=TRUE)
**AIC**(null.EasyRead, Condition.EasyRead, Vac.EasyRead,VacCondition.EasyRead)

## df AIC
## null.EasyRead 6 150.4334
## Condition.EasyRead 7 152.0568
## Vac.EasyRead 7 151.2873
## VacCondition.EasyRead 8 152.7940

**library**(psych)
**describe**(das$EasyImagine)

## vars n mean sd median trimmed mad min max range skew kurtosis se
## X1 1 53 5.94 1.31 6 6.21 1.48 1 7 6 -1.88 3.68 0.18

**describeBy**(das$EasyImagine, das$CONDITION)

##
## Descriptive statistics by group
## group: 1
## vars n mean sd median trimmed mad min max range skew kurtosis se
## X1 1 28 5.79 1.45 6 6.04 1.48 1 7 6 -1.82 3.16 0.27
## --------------------------------------------------------
## group: 2
## vars n mean sd median trimmed mad min max range skew kurtosis se
## X1 1 25 6.12 1.13 6 6.33 1.48 3 7 4 -1.55 1.89 0.23

null.EasyImagine<-**polr**(**as.ordered**(EasyImagine)~1,data=das, Hess=TRUE)
Condition.EasyImagine<-**polr**(**as.ordered**(EasyImagine)~**as.factor**(CONDITION),data=das, Hess=TRUE)
Vac.EasyImagine<-**polr**(**as.ordered**(EasyImagine)~Vac,data=das, Hess=TRUE)
VacCondition.EasyImagine<-**polr**(**as.ordered**(EasyImagine)~Vac+**as.factor**(CONDITION),data=das, Hess=TRUE)
**AIC**(null.EasyImagine, Condition.EasyImagine, Vac.EasyImagine,VacCondition.EasyImagine)

## df AIC
## null.EasyImagine 6 152.7421
## Condition.EasyImagine 7 153.7148
## Vac.EasyImagine 7 154.4386
## VacCondition.EasyImagine 8 155.5275

**library**(psych)
**describe**(das$Plausibility)

## vars n mean sd median trimmed mad min max range skew kurtosis se
## X1 1 53 5.26 1.53 6 5.44 1.48 2 7 5 -0.78 -0.36 0.21

**describeBy**(das$Plausibility, das$CONDITION)

##
## Descriptive statistics by group
## group: 1
## vars n mean sd median trimmed mad min max range skew kurtosis se
## X1 1 28 5.29 1.49 6 5.42 1.48 2 7 5 -0.74 -0.44 0.28
## --------------------------------------------------------
## group: 2
## vars n mean sd median trimmed mad min max range skew kurtosis se
## X1 1 25 5.24 1.61 6 5.38 1.48 2 7 5 -0.77 -0.52 0.32

null.Plausibility<-**polr**(**as.ordered**(Plausibility)~1,data=das, Hess=TRUE)
Condition.Plausibility<-**polr**(**as.ordered**(Plausibility)~**as.factor**(CONDITION),data=das, Hess=TRUE)
Vac.Plausibility<-**polr**(**as.ordered**(Plausibility)~Vac,data=das, Hess=TRUE)
VacCondition.Plausibility<-**polr**(**as.ordered**(Plausibility)~Vac+**as.factor**(CONDITION),data=das, Hess=TRUE)
**AIC**(null.Plausibility, Condition.Plausibility, Vac.Plausibility,VacCondition.Plausibility)

## df AIC
## null.Plausibility 5 184.0722
## Condition.Plausibility 6 186.0721
## Vac.Plausibility 6 185.7686
## VacCondition.Plausibility 7 187.7645

**REGRET**

**library**(psych)
**describe**(das$Regret)

## vars n mean sd median trimmed mad min max range skew kurtosis se
## X1 1 53 2.89 1.93 3 2.7 2.97 1 7 6 0.54 -1.06 0.26

**describeBy**(das$Regret, das$CONDITION)

##
## Descriptive statistics by group
## group: 1
## vars n mean sd median trimmed mad min max range skew kurtosis se
## X1 1 28 3.04 1.82 3 2.92 2.97 1 7 6 0.38 -1 0.34
## --------------------------------------------------------
## group: 2
## vars n mean sd median trimmed mad min max range skew kurtosis se
## X1 1 25 2.72 2.07 2 2.52 1.48 1 7 6 0.68 -1.15 0.41

null.Regret<-**polr**(**as.ordered**(Regret)~1,data=das, Hess=TRUE)
Condition.Regret<-**polr**(**as.ordered**(Regret)~**as.factor**(CONDITION),data=das, Hess=TRUE)
Vac.Regret<-**polr**(**as.ordered**(Regret)~Vac,data=das, Hess=TRUE)
VacCondition.Regret<-**polr**(**as.ordered**(Regret)~Vac+**as.factor**(CONDITION),data=das, Hess=TRUE)
VacXCondition.Regret<-**polr**(**as.ordered**(Regret)~Vac***as.factor**(CONDITION),data=das, Hess=TRUE)
**AIC**(null.Regret, Condition.Regret, Vac.Regret,VacCondition.Regret, VacXCondition.Regret)

## df AIC
## null.Regret 6 193.4115
## Condition.Regret 7 194.8114
## Vac.Regret 7 195.3049
## VacCondition.Regret 8 196.7585
## VacXCondition.Regret 9 198.5815

**DISGUST**

**library**(psych)
**describe**(das$Disgust)

## vars n mean sd median trimmed mad min max range skew kurtosis se
## X1 1 53 4.49 1.56 5 4.56 1.48 1 7 6 -0.47 -0.29 0.21

**describeBy**(das$Disgust, das$CONDITION)

##
## Descriptive statistics by group
## group: 1
## vars n mean sd median trimmed mad min max range skew kurtosis se
## X1 1 28 4.25 1.67 4 4.29 1.48 1 7 6 -0.34 -0.42 0.32
## --------------------------------------------------------
## group: 2
## vars n mean sd median trimmed mad min max range skew kurtosis se
## X1 1 25 4.76 1.42 5 4.81 1.48 2 7 5 -0.51 -0.56 0.28

null.Disgust<-**polr**(**as.ordered**(Disgust)~1,data=das, Hess=TRUE)
Condition.Disgust<-**polr**(**as.ordered**(Disgust)~**as.factor**(CONDITION),data=das, Hess=TRUE)
Vac.Disgust<-**polr**(**as.ordered**(Disgust)~Vac,data=das, Hess=TRUE)
VacCondition.Disgust<-**polr**(**as.ordered**(Disgust)~Vac+**as.factor**(CONDITION),data=das, Hess=TRUE)
VacXCondition.Disgust<-**polr**(**as.ordered**(Disgust)~Vac***as.factor**(CONDITION),data=das, Hess=TRUE)
**AIC**(null.Disgust, Condition.Disgust, Vac.Disgust,VacCondition.Disgust, VacXCondition.Disgust)

## df AIC
## null.Disgust 6 199.3016
## Condition.Disgust 7 199.6163
## Vac.Disgust 7 200.8131
## VacCondition.Disgust 8 200.8486
## VacXCondition.Disgust 9 202.7529

**FEAR**

**library**(psych)
**describe**(das$Fear)

## vars n mean sd median trimmed mad min max range skew kurtosis se
## X1 1 53 5.26 1.53 6 5.44 1.48 2 7 5 -0.78 -0.36 0.21

**describeBy**(das$Fear, das$CONDITION)

##
## Descriptive statistics by group
## group: 1
## vars n mean sd median trimmed mad min max range skew kurtosis se
## X1 1 28 5.29 1.49 6 5.42 1.48 2 7 5 -0.74 -0.44 0.28
## --------------------------------------------------------
## group: 2
## vars n mean sd median trimmed mad min max range skew kurtosis se
## X1 1 25 5.24 1.61 6 5.38 1.48 2 7 5 -0.77 -0.52 0.32

null.Fear<-**polr**(**as.ordered**(Fear)~1,data=das, Hess=TRUE)
Condition.Fear<-**polr**(**as.ordered**(Fear)~**as.factor**(CONDITION),data=das, Hess=TRUE)
Vac.Fear<-**polr**(**as.ordered**(Fear)~Vac,data=das, Hess=TRUE)
VacCondition.Fear<-**polr**(**as.ordered**(Fear)~Vac+**as.factor**(CONDITION),data=das, Hess=TRUE)
VacXCondition.Fear<-**polr**(**as.ordered**(Fear)~Vac***as.factor**(CONDITION),data=das, Hess=TRUE)
**AIC**(null.Fear, Condition.Fear, Vac.Fear,VacCondition.Fear, VacXCondition.Fear)

## df AIC
## null.Fear 5 184.0722
## Condition.Fear 6 186.0721
## Vac.Fear 6 185.7686
## VacCondition.Fear 7 187.7645
## VacXCondition.Fear 8 188.4387

**ANGER**

**library**(psych)
**describe**(das$Anger)

## vars n mean sd median trimmed mad min max range skew kurtosis se
## X1 1 53 4.02 1.99 4 4.02 2.97 1 7 6 -0.24 -1.24 0.27

**describeBy**(das$Anger, das$CONDITION)

##
## Descriptive statistics by group
## group: 1
## vars n mean sd median trimmed mad min max range skew kurtosis se
## X1 1 28 3.75 1.97 4 3.75 2.97 1 7 6 -0.14 -1.47 0.37
## --------------------------------------------------------
## group: 2
## vars n mean sd median trimmed mad min max range skew kurtosis se
## X1 1 25 4.32 1.99 5 4.38 1.48 1 7 6 -0.37 -1.09 0.4

null.Anger<-**polr**(**as.ordered**(Anger)~1,data=das, Hess=TRUE)
Condition.Anger<-**polr**(**as.ordered**(Anger)~**as.factor**(CONDITION),data=das, Hess=TRUE)
Vac.Anger<-**polr**(**as.ordered**(Anger)~Vac,data=das, Hess=TRUE)
VacCondition.Anger<-**polr**(**as.ordered**(Anger)~Vac+**as.factor**(CONDITION),data=das, Hess=TRUE)
VacXCondition.Anger<-**polr**(**as.ordered**(Anger)~Vac***as.factor**(CONDITION),data=das, Hess=TRUE)
**AIC**(null.Anger, Condition.Anger, Vac.Anger,VacCondition.Anger, VacXCondition.Anger)

## df AIC
## null.Anger 6 211.6828
## Condition.Anger 7 212.6882
## Vac.Anger 7 213.6827
## VacCondition.Anger 8 214.6830
## VacXCondition.Anger 9 216.2346

**SADNESS**

**library**(psych)
**describe**(das$Sadness)

## vars n mean sd median trimmed mad min max range skew kurtosis se
## X1 1 53 2.98 1.87 3 2.81 2.97 1 7 6 0.48 -1.05 0.26

**describeBy**(das$Sadness, das$CONDITION)

##
## Descriptive statistics by group
## group: 1
## vars n mean sd median trimmed mad min max range skew kurtosis se
## X1 1 28 3 1.91 2.5 2.83 2.22 1 7 6 0.65 -0.75 0.36
## --------------------------------------------------------
## group: 2
## vars n mean sd median trimmed mad min max range skew kurtosis se
## X1 1 25 2.96 1.86 3 2.86 2.97 1 6 5 0.24 -1.62 0.37

null.Sadness<-**polr**(**as.ordered**(Sadness)~1,data=das, Hess=TRUE)
Condition.Sadness<-**polr**(**as.ordered**(Sadness)~**as.factor**(CONDITION),data=das, Hess=TRUE)
Vac.Sadness<-**polr**(**as.ordered**(Sadness)~Vac,data=das, Hess=TRUE)
VacCondition.Sadness<-**polr**(**as.ordered**(Sadness)~Vac+**as.factor**(CONDITION),data=das, Hess=TRUE)
VacXCondition.Sadness<-**polr**(**as.ordered**(Sadness)~Vac***as.factor**(CONDITION),data=das, Hess=TRUE)
**AIC**(null.Sadness, Condition.Sadness, Vac.Sadness,VacCondition.Sadness, VacXCondition.Sadness)

## df AIC
## null.Sadness 6 201.0938
## Condition.Sadness 7 203.0896
## Vac.Sadness 7 202.3122
## VacCondition.Sadness 8 204.3110
## VacXCondition.Sadness 9 204.9817

**JOY**

**library**(psych)
**describe**(das$Joy)

## vars n mean sd median trimmed mad min max range skew kurtosis se
## X1 1 53 4.25 2.05 5 4.3 2.97 1 7 6 -0.23 -1.25 0.28

**describeBy**(das$Joy, das$CONDITION)

##
## Descriptive statistics by group
## group: 1
## vars n mean sd median trimmed mad min max range skew kurtosis se
## X1 1 28 4.21 2.06 4 4.25 2.97 1 7 6 -0.16 -1.32 0.39
## --------------------------------------------------------
## group: 2
## vars n mean sd median trimmed mad min max range skew kurtosis se
## X1 1 25 4.28 2.07 5 4.33 2.97 1 7 6 -0.31 -1.31 0.41

null.Joy<-**polr**(**as.ordered**(Joy)~1,data=das, Hess=TRUE)
Condition.Joy<-**polr**(**as.ordered**(Joy)~**as.factor**(CONDITION),data=das, Hess=TRUE)
Vac.Joy<-**polr**(**as.ordered**(Joy)~Vac,data=das, Hess=TRUE)
VacCondition.Joy<-**polr**(**as.ordered**(Joy)~Vac+**as.factor**(CONDITION),data=das, Hess=TRUE)
VacXCondition.Joy<-**polr**(**as.ordered**(Joy)~Vac***as.factor**(CONDITION),data=das, Hess=TRUE)
**AIC**(null.Joy, Condition.Joy, Vac.Joy,VacCondition.Joy, VacXCondition.Joy)

## df AIC
## null.Joy 6 215.8688
## Condition.Joy 7 217.8502
## Vac.Joy 7 217.6429
## VacCondition.Joy 8 219.6059
## VacXCondition.Joy 9 221.2102

**COMPASSION**

**library**(psych)
**describe**(das$Compassion)

## vars n mean sd median trimmed mad min max range skew kurtosis se
## X1 1 53 1.45 1.14 1 1.16 0 1 7 6 2.93 9.37 0.16

**describeBy**(das$Compassion, das$CONDITION)

##
## Descriptive statistics by group
## group: 1
## vars n mean sd median trimmed mad min max range skew kurtosis se
## X1 1 28 1.61 1.37 1 1.33 0 1 7 6 2.53 6.31 0.26
## --------------------------------------------------------
## group: 2
## vars n mean sd median trimmed mad min max range skew kurtosis se
## X1 1 25 1.28 0.79 1 1.1 0 1 4 3 2.4 4.37 0.16

null.Compassion<-**polr**(**as.ordered**(Compassion)~1,data=das, Hess=TRUE)
Condition.Compassion<-**polr**(**as.ordered**(Compassion)~**as.factor**(CONDITION),data=das, Hess=TRUE)
Vac.Compassion<-**polr**(**as.ordered**(Compassion)~Vac,data=das, Hess=TRUE)
VacCondition.Compassion<-**polr**(**as.ordered**(Compassion)~Vac+**as.factor**(CONDITION),data=das, Hess=TRUE)
VacXCondition.Compassion<-**polr**(**as.ordered**(Compassion)~Vac***as.factor**(CONDITION),data=das, Hess=TRUE)
**AIC**(null.Compassion, Condition.Compassion, Vac.Compassion,VacCondition.Compassion, VacXCondition.Compassion)

## df AIC
## null.Compassion 4 85.61271
## Condition.Compassion 5 86.22526
## Vac.Compassion 5 87.47276
## VacCondition.Compassion 6 88.15469
## VacXCondition.Compassion 7 90.14849

**SURPRISE**

**library**(psych)
**describe**(das$Surprise)

## vars n mean sd median trimmed mad min max range skew kurtosis se
## X1 1 53 2.34 1.7 2 2.12 1.48 1 7 6 0.98 -0.37 0.23

**describeBy**(das$Surprise, das$CONDITION)

##
## Descriptive statistics by group
## group: 1
## vars n mean sd median trimmed mad min max range skew kurtosis se
## X1 1 28 2.36 1.57 2 2.17 1.48 1 7 6 1.14 0.67 0.3
## --------------------------------------------------------
## group: 2
## vars n mean sd median trimmed mad min max range skew kurtosis se
## X1 1 25 2.32 1.86 1 2.14 0 1 6 5 0.81 -1.22 0.37

null.Surprise<-**polr**(**as.ordered**(Surprise)~1,data=das, Hess=TRUE)
Condition.Surprise<-**polr**(**as.ordered**(Surprise)~**as.factor**(CONDITION),data=das, Hess=TRUE)
Vac.Surprise<-**polr**(**as.ordered**(Surprise)~Vac,data=das, Hess=TRUE)
VacCondition.Surprise<-**polr**(**as.ordered**(Surprise)~Vac+**as.factor**(CONDITION),data=das, Hess=TRUE)
VacXCondition.Surprise<-**polr**(**as.ordered**(Surprise)~Vac***as.factor**(CONDITION),data=das, Hess=TRUE)
**AIC**(null.Surprise, Condition.Surprise, Vac.Surprise,VacCondition.Surprise, VacXCondition.Surprise)

## df AIC
## null.Surprise 6 167.9228
## Condition.Surprise 7 169.4341
## Vac.Surprise 7 168.5347
## VacCondition.Surprise 8 170.2588
## VacXCondition.Surprise 9 172.2346

**CONFUSSION**

**library**(psych)
**describe**(das$Confussion)

## vars n mean sd median trimmed mad min max range skew kurtosis se
## X1 1 53 3.36 2.21 3 3.21 2.97 1 7 6 0.33 -1.36 0.3

**describeBy**(das$Confussion, das$CONDITION)

##
## Descriptive statistics by group
## group: 1
## vars n mean sd median trimmed mad min max range skew kurtosis se
## X1 1 28 3.57 2.2 3 3.5 2.97 1 7 6 0.23 -1.43 0.42
## --------------------------------------------------------
## group: 2
## vars n mean sd median trimmed mad min max range skew kurtosis se
## X1 1 25 3.12 2.24 3 2.95 2.97 1 7 6 0.43 -1.38 0.45

null.Confussion<-**polr**(**as.ordered**(Confussion)~1,data=das, Hess=TRUE)
Condition.Confussion<-**polr**(**as.ordered**(Confussion)~**as.factor**(CONDITION),data=das, Hess=TRUE)
Vac.Confussion<-**polr**(**as.ordered**(Confussion)~Vac,data=das, Hess=TRUE)
VacCondition.Confussion<-**polr**(**as.ordered**(Confussion)~Vac+**as.factor**(CONDITION),data=das, Hess=TRUE)
VacXCondition.Confussion<-**polr**(**as.ordered**(Confussion)~Vac***as.factor**(CONDITION),data=das, Hess=TRUE)
**AIC**(null.Confussion, Condition.Confussion, Vac.Confussion,VacCondition.Confussion, VacXCondition.Confussion)

## df AIC
## null.Confussion 6 200.0509
## Condition.Confussion 7 201.3821
## Vac.Confussion 7 200.2532
## VacCondition.Confussion 8 201.7406
## VacXCondition.Confussion 9 203.0501

# E: R SCRIPT FOR EXPERIMENT

**LACK OF EVIDENCE FOR THE OMISSION AND THE CONGRUITY BIASES AS APPLIED TO VACCINATION**

Angel V. Jimenez

10 April 2018

The following analyses are based on the preregistred script for this experiment (<https://osf.io/2dgvm/>) included within the preregistered documents (“Beyond Vaccine Decissions: The omission bias in severity ratings and free recall”,<https://osf.io/gebc7/>).

**DATA FILE**

*# Open data file*
d<-**read.csv**("C://files/Angel/omission/experiment/omission_bias_experiment.csv")
*# Explore the different values of each variable*
**str**(d)

## 'data.frame': 261 obs. of 24 variables:
## $ Collection : Factor w/ 4 levels "first","fourth",..: 1 1 1 1 1 1 1 1 1 1 ...
## $ Participant : int 277308 277358 277375 277386 277408 277483 277497 277533 277610 277619 ...
## $ Condition : Factor w/ 2 levels "Commission","Omission": 1 1 1 1 1 1 1 1 1 1 ...
## $ Recall : int 16 8 12 6 14 11 15 11 10 11 ...
## $ Recall_Practice : int 2 1 4 3 1 3 1 1 1 1 ...
## $ Total : int 18 9 16 9 15 14 16 12 11 12 ...
## $ False_Memories : int 0 0 0 0 0 0 0 0 0 0 ...
## $ Notes : Factor w/ 104 levels "","ache = soreness",..: 34 1 78 71 1 1 1 1 20 1 ...
## $ Qualitative_Recall: Factor w/ 261 levels "a headache, anxiety, dizzines, a cough, swilling, an earache, nausea, fever, temperature, lightheaded",..: 165 197 92 233 44 136 254 105 10 175 ...
## $ Prescreening : Factor w/ 2 levels "Antivaxx","Provaxx": 1 1 1 1 1 1 1 1 1 1 ...
## $ Age : int 32 48 18 39 30 38 25 25 30 40 ...
## $ English : int 1 1 1 1 1 1 1 1 1 1 ...
## $ Gender : int 2 2 2 2 2 2 2 2 2 2 ...
## $ Education : int 3 2 2 2 5 3 3 3 2 3 ...
## $ Nationality : int 1 1 1 1 2 1 1 1 1 1 ...
## $ Number_Children : int 0 5 0 1 0 3 0 1 0 4 ...
## $ Avg_RT : num 1526 1432 1302 1268 1192 ...
## $ Avg_Severity : num 4.04 4.17 3.83 4.04 4.25 ...
## $ Failure : int 8 16 12 18 10 13 9 13 14 13 ...
## $ Weight : int 24 24 24 24 24 24 24 24 24 24 ...
## $ Time1 : num 80998 35786 32479 4651 33844 ...
## $ Time2 : num 29176 17566 13142 1627 7204 ...
## $ Vac1 : int 5 2 6 7 7 6 2 5 5 1 ...
## $ Vac2 : int 4 1 6 6 6 6 2 4 5 1 ...

*# Summary statistics for each variable*
**summary**(d)

## Collection Participant Condition Recall
## first :198 Min. :277273 Commission:134 Min. : 2.00
## fourth: 4 1st Qu.:277497 Omission :127 1st Qu.: 9.00
## second: 50 Median :278249 Median :11.00
## third : 9 Mean :281167 Mean :11.11
## 3rd Qu.:279632 3rd Qu.:13.00
## Max. :304913 Max. :20.00
## NA's :8
## Recall_Practice Total False_Memories
## Min. :0.000 Min. : 3.00 Min. :0.0000
## 1st Qu.:1.000 1st Qu.:11.00 1st Qu.:0.0000
## Median :2.000 Median :13.00 Median :0.0000
## Mean :2.095 Mean :13.22 Mean :0.2332
## 3rd Qu.:3.000 3rd Qu.:16.00 3rd Qu.:0.0000
## Max. :5.000 Max. :23.00 Max. :2.0000
## NA's :8 NA's :8 NA's :8
## Notes
## :137
## sore throat = hoarseness : 6
## aching = soreness : 3
## sickness = nausea : 3
## stomach cramps = abdominal pain: 3
## TECHNICAL PROBLEM : 3
## (Other) :106
## Qualitative_Recall
## a headache, anxiety, dizzines, a cough, swilling, an earache, nausea, fever, temperature, lightheaded : 1
## A headache, dizziness, toothache, crying, nausea, bleeding, vomiting, rash, numbness, fever, sweating, chills, loss of appetite, : 1
## abdominal pain, vomiting, insomnia, crying, fever, bleeding, tootache, wheezing, cough, earache, chills, soreness, swelling : 1
## Aches, Diareah, Sickness, Headaches, Dizziness, Tootache, Bleeding, Swelling, Lightheadedness, Muscle ache, Insomnia, Bloating, Crying : 1
## Alô i answered das that It das notas tudo top It das notas Wellington between 4 André 6 : 1
## anxiety, abdominal pain, nausea, itchy eyes, chills, fever, vomiting, diarrhea, loss of appetite, weight loss, shivering, crying, toothache, muscle ache, headache, soreness, : 1
## (Other) :255
## Prescreening Age English Gender
## Antivaxx:134 Min. :18.00 Min. :1.000 Min. :1.000
## Provaxx :127 1st Qu.:30.00 1st Qu.:1.000 1st Qu.:2.000
## Median :37.00 Median :1.000 Median :2.000
## Mean :38.15 Mean :1.004 Mean :1.996
## 3rd Qu.:45.00 3rd Qu.:1.000 3rd Qu.:2.000
## Max. :60.00 Max. :2.000 Max. :2.000
##
## Education Nationality Number_Children Avg_RT
## Min. :1.00 Min. :1.00 Min. :0.000 Min. : 561.8
## 1st Qu.:3.00 1st Qu.:1.00 1st Qu.:0.000 1st Qu.:1106.2
## Median :3.00 Median :1.00 Median :1.000 Median :1262.2
## Mean :3.33 Mean :1.18 Mean :1.383 Mean :1292.5
## 3rd Qu.:4.00 3rd Qu.:1.00 3rd Qu.:2.000 3rd Qu.:1432.0
## Max. :6.00 Max. :3.00 Max. :6.000 Max. :2851.5
##
## Avg_Severity Failure Weight Time1
## Min. :1.000 Min. : 4.00 Min. :24 Min. : 1295
## 1st Qu.:3.583 1st Qu.:11.00 1st Qu.:24 1st Qu.: 22192
## Median :4.087 Median :13.00 Median :24 Median : 34989
## Mean :4.143 Mean :12.89 Mean :24 Mean : 43334
## 3rd Qu.:4.739 3rd Qu.:15.00 3rd Qu.:24 3rd Qu.: 51681
## Max. :6.870 Max. :22.00 Max. :24 Max. :396979
## NA's :8
## Time2 Vac1 Vac2
## Min. : 57 Min. :1.000 Min. :1.000
## 1st Qu.: 5972 1st Qu.:4.000 1st Qu.:3.000
## Median : 11694 Median :6.000 Median :6.000
## Mean : 16890 Mean :5.291 Mean :4.943
## 3rd Qu.: 21479 3rd Qu.:7.000 3rd Qu.:7.000
## Max. :172697 Max. :7.000 Max. :7.000
##

**DESCRIPTIVES (WHOLE SAMPLE OF PARTICIPANTS)**

*# Number of participants*
**length**(d$Participant)

## [1] 261

*# Frequencies by gender*
**library**(plyr)

## Warning: package 'plyr' was built under R version 3.4.4

**count**(d$Gender) *# 1 = male, 2 = female*

## x freq
## 1 1 1
## 2 2 260

*# Frequencies by Nationality*
**count**(d$Nationality) *# 1 = British, 2 = American, 3 =Other*

## x freq
## 1 1 216
## 2 2 43
## 3 3 2

*# Frequencies by Languague*
**count**(d$English) *# English native Speaker = 1, Non-English native Speaker = 2*

## x freq
## 1 1 260
## 2 2 1

*# Descriptives for Age*
**library**(psych)
**describe**(d$Age)

## vars n mean sd median trimmed mad min max range skew kurtosis
## X1 1 261 38.15 10.37 37 37.76 11.86 18 60 42 0.3 -0.84
## se
## X1 0.64

*# Histogram of Age*
breaks<-**c**(15, 20, 25, 30, 35, 40, 45, 50, 55, 60, 65, 70)
**hist**(d$Age,
 main="Histogram for Age",
 xlab="Age",
 border="blue",
 col="green",
 breaks = breaks,
 xlim=**c**(15,70),
 ylim=**c**(0,50),
 prob = FALSE,
 xaxt ="n")
**axis**(side=1, at=**seq**(15,70,5), labels=**seq**(15,70,5))


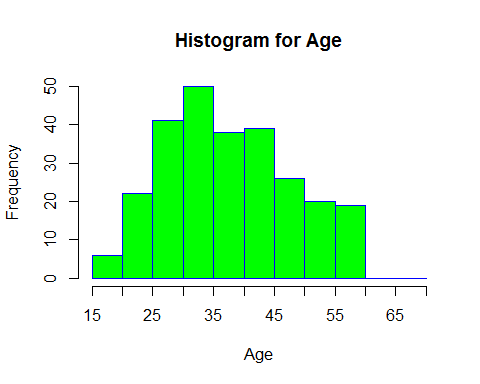
 N=261 participants (1 male, 260 females) aged 18-60 (M=38.15, SD = 10.37) with American (N=43), British (N=216) or other nationality (N=2). One participants reported not being an English native speaker.

**DATA EXCLUSION**

*# Selection of participants in first time of data collection*
dfirst<-d[ **which**(d$Collection == "first"),]
*# Number of participants after applying the exclusion criteria*
**length**(dfirst$Participant) *# Technical problem led to collect 198 responses instead of 200 responses*

## [1] 198

*# Exclusion of participants according to the criteria informed in the pre-registration for first time of data collection*
dfirst2<-dfirst[**which**((dfirst$Time1>=23000 | dfirst$Time2>=23000) & dfirst$Gender == "2" & dfirst$Age <= 60 & dfirst$English == "1" & dfirst$Nationality != "3"),]
**length**(dfirst2$Participant)

## [1] 149

*# Table of included responses per condition after applying the exclusion criteria (first time data collection)*
**table**(dfirst2$Condition, dfirst2$Prescreening)

##
## Antivaxx Provaxx
## Commission 37 41
## Omission 36 35

*# Table of responses per condition that were neccessary to collect in the second time of data collection*
50-**table**(dfirst2$Condition, dfirst2$Prescreening)

##
## Antivaxx Provaxx
## Commission 13 9
## Omission 14 15

*# Selection of participants in first and second time of data collection*
dsecond<-d[ **which**(d$Collection == "first" | d$Collection == "second"),]

*# Exclusion of participants according to the criteria informed in the pre-registration for second time of data collection*
dsecond2<-dsecond[**which**((dsecond$Time1>=23000 | dsecond$Time2>=23000) & dsecond$Gender == "2" & dsecond$Age <= 60 & dsecond$English == "1" & dsecond$Nationality != "3"),]

*# Table of included responses per condition after applying the exclusion criteria (second time data collection)*
**table**(dsecond2$Condition, dsecond2$Prescreening)

##
## Antivaxx Provaxx
## Commission 47 52
## Omission 46 49

*# Table of responses per condition that were neccessary to collect in the thrid time of data collection*
50-**table**(dsecond2$Condition, dsecond2$Prescreening)

##
## Antivaxx Provaxx
## Commission 3 -2
## Omission 4 1

*# Selection of participants in first, second and third time of data collection*
dthird<-d[ **which**(d$Collection == "first" | d$Collection == "second" | d$Collection == "third"),]
*# Exclusion of participants according to the criter informed in the pre-registration for third time of data collection*
dthird2<-dthird[**which**((dthird$Time1>=23000 | dthird$Time2>=23000) & dthird$Gender == "2" & dthird$Age <= 60 & dthird$English == "1" & dthird$Nationality != "3"),]

*# Table of included responses per condition in third time of data collection*
**table**(dthird2$Condition, dthird2$Prescreening)

##
## Antivaxx Provaxx
## Commission 49 52
## Omission 48 50

*# Table of responses per condition that were neccessary to collect for fourth time of data collection*
50-**table**(dthird2$Condition, dthird2$Prescreening)

##
## Antivaxx Provaxx
## Commission 1 -2
## Omission 2 0

*# Selection of participants in first, second, third, and four time of data collection*
dfourth2<-d[**which**((d$Time1>=23000 | d$Time2>=23000) & d$Gender == "2" & d$Age <= 60 & d$English == "1" & d$Nationality != "3"),]

*# Table of included responses per condition in fourth time of data collection*
**table**(dfourth2$Condition, dfourth2$Prescreening)

##
## Antivaxx Provaxx
## Commission 50 52
## Omission 50 50

*# Table of responses per condition that were neccessary to collect*
50-**table**(dfourth2$Condition, dfourth2$Prescreening) *# 0*

##
## Antivaxx Provaxx
## Commission 0 -2
## Omission 0 0

A technical problem made that we had 52 participants, instead of 50, in the Comission Condition for people who reported pro-vaccination attitudes in the pre-screening.

**DESCRIPTIVES (REDUCED SAMPLE OF PARTICIPANTS)**

*# Change name of dataset*
da<-dfourth2
*# Descriptives for reduced dataset*
*# Number of participants*
**length**(da$Participant)

## [1] 202

*# Frequencies by gender*
**count**(da$Gender) *# 1 = male, 2 = female*

## x freq
## 1 2 202

*# Frequencies by Nationality*
**count**(da$Nationality) *# 1 = British, 2 = American*

## x freq
## 1 1 164
## 2 2 38

*# Summaries for Age*
**library**(psych)
**describe**(da$Age)

## vars n mean sd median trimmed mad min max range skew kurtosis
## X1 1 202 38.61 10.39 37.5 38.28 11.12 18 60 42 0.25 -0.92
## se
## X1 0.73

*# Histogram of Age*
breaks<-**c**(15, 20, 25, 30, 35, 40, 45, 50, 55, 60, 65, 70)
**hist**(da$Age,
 main="Histogram for Age",
 xlab="Age",
 border="blue",
 col="green",
 breaks = breaks,
 xlim=**c**(15,70),
 ylim=**c**(0,35),
 prob = FALSE,
 xaxt ="n")
**axis**(side=1, at=**seq**(15,70,5), labels=**seq**(15,70,5))


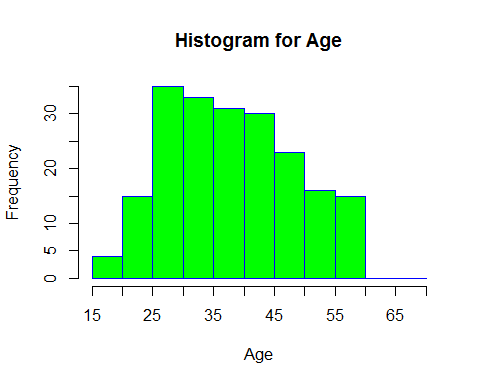
 202 female participants aged 18-60 (M=38.61, SD=10.39) with American (N=38) and British (N=164) nationalities.

**TRANSFORMATIONS AND STANDARIZATION OF VARIABLES**

*# In order to be able to compare the strength of binary (e.g. Condition) and "continuous" predictors, we standarize by substracting the mean and dividing by 2 SDs. See Gelman (2008).*

*# Vaccination Attitudes*
*# Rescalate from +1 to +7 to -3 to +3 and divided by the 2 items*
da$Vac<-((da$Vac1+da$Vac2)/2-4)
*# Standarized Vaccination Attitudes*
da$Z.Vac<-(da$Vac-**mean**(da$Vac))/(2***sd**(da$Vac))

*# Average Severity Ratings*
*# Standarized Average Severity Ratings*
da$Z.Avg_Severity<-(da$Avg_Severity-**mean**(da$Avg_Severity))/(2***sd**(da$Avg_Severity))

*# Average Reaction Times*
*# Standarized Average Reaction Times*
da$Z.Avg_RTs<-(da$Avg_RT-**mean**(da$Avg_RT))/(2***sd**(da$Avg_RT))

*# Check that everything is fine*
**str**(da)

## 'data.frame': 202 obs. of 28 variables:
## $ Collection : Factor w/ 4 levels "first","fourth",..: 1 1 1 1 1 1 1 1 1 1 ...
## $ Participant : int 277308 277358 277375 277408 277483 277497 277533 277610 277619 277955 ...
## $ Condition : Factor w/ 2 levels "Commission","Omission": 1 1 1 1 1 1 1 1 1 1 ...
## $ Recall : int 16 8 12 14 11 15 11 10 11 11 ...
## $ Recall_Practice : int 2 1 4 1 3 1 1 1 1 4 ...
## $ Total : int 18 9 16 15 14 16 12 11 12 15 ...
## $ False_Memories : int 0 0 0 0 0 0 0 0 0 0 ...
## $ Notes : Factor w/ 104 levels "","ache = soreness",..: 34 1 78 1 1 1 1 20 1 1 ...
## $ Qualitative_Recall: Factor w/ 261 levels "a headache, anxiety, dizzines, a cough, swilling, an earache, nausea, fever, temperature, lightheaded",..: 165 197 92 44 136 254 105 10 175 192 ...
## $ Prescreening : Factor w/ 2 levels "Antivaxx","Provaxx": 1 1 1 1 1 1 1 1 1 1 ...
## $ Age : int 32 48 18 30 38 25 25 30 40 40 ...
## $ English : int 1 1 1 1 1 1 1 1 1 1 ...
## $ Gender : int 2 2 2 2 2 2 2 2 2 2 ...
## $ Education : int 3 2 2 5 3 3 3 2 3 2 ...
## $ Nationality : int 1 1 1 2 1 1 1 1 1 1 ...
## $ Number_Children : int 0 5 0 0 3 0 1 0 4 2 ...
## $ Avg_RT : num 1526 1432 1302 1192 1029 ...
## $ Avg_Severity : num 4.04 4.17 3.83 4.25 4.33 ...
## $ Failure : int 8 16 12 10 13 9 13 14 13 13 ...
## $ Weight : int 24 24 24 24 24 24 24 24 24 24 ...
## $ Time1 : num 80998 35786 32479 33844 28658 ...
## $ Time2 : num 29176 17566 13142 7204 18022 ...
## $ Vac1 : int 5 2 6 7 6 2 5 5 1 6 ...
## $ Vac2 : int 4 1 6 6 6 2 4 5 1 6 ...
## $ Vac : num 0.5 -2.5 2 2.5 2 -2 0.5 1 -3 2 ...
## $ Z.Vac : num -0.164 -0.941 0.225 0.355 0.225 ...
## $ Z.Avg_Severity : num -0.0706 0.0107 -0.1987 0.0574 0.1087 ...
## $ Z.Avg_RTs : num 0.4133 0.2348 -0.0114 -0.2197 -0.5289 ...

**summary**(da)

## Collection Participant Condition Recall
## first :149 Min. :277273 Commission:102 Min. : 3.00
## fourth: 3 1st Qu.:277502 Omission :100 1st Qu.:10.00
## second: 45 Median :278254 Median :11.00
## third : 5 Mean :281179 Mean :11.51
## 3rd Qu.:287503 3rd Qu.:14.00
## Max. :304913 Max. :20.00
## NA's :5
## Recall_Practice Total False_Memories
## Min. :0.000 Min. : 4.00 Min. :0.0000
## 1st Qu.:1.000 1st Qu.:12.00 1st Qu.:0.0000
## Median :2.000 Median :14.00 Median :0.0000
## Mean :2.198 Mean :13.72 Mean :0.1929
## 3rd Qu.:3.000 3rd Qu.:16.00 3rd Qu.:0.0000
## Max. :5.000 Max. :23.00 Max. :2.0000
## NA's :5 NA's :5 NA's :5
## Notes
## :112
## sore throat = hoarseness : 6
## aching = soreness : 3
## SAYS "I CAN'T REMBEMBER" : 2
## sickness = nausea : 2
## stomach ache = abdominal pain: 2
## (Other) : 75
## Qualitative_Recall
## a headache, anxiety, dizzines, a cough, swilling, an earache, nausea, fever, temperature, lightheaded : 1
## A headache, dizziness, toothache, crying, nausea, bleeding, vomiting, rash, numbness, fever, sweating, chills, loss of appetite, : 1
## Aches, Diareah, Sickness, Headaches, Dizziness, Tootache, Bleeding, Swelling, Lightheadedness, Muscle ache, Insomnia, Bloating, Crying : 1
## anxiety, abdominal pain, nausea, itchy eyes, chills, fever, vomiting, diarrhea, loss of appetite, weight loss, shivering, crying, toothache, muscle ache, headache, soreness, : 1
## Anxiety, bleeding, light-headedness, swelling, earache, headache, crying, hoarseness, dry mouth, chills, rash, : 1
## anxiety, nausea, chills, headache, vomiting, crying, cough, dizziness, shivers, dry mouth, rash, itch eyes, : 1
## (Other) :196
## Prescreening Age English Gender Education
## Antivaxx:100 Min. :18.00 Min. :1 Min. :2 Min. :1.000
## Provaxx :102 1st Qu.:30.00 1st Qu.:1 1st Qu.:2 1st Qu.:3.000
## Median :37.50 Median :1 Median :2 Median :3.000
## Mean :38.61 Mean :1 Mean :2 Mean :3.322
## 3rd Qu.:47.00 3rd Qu.:1 3rd Qu.:2 3rd Qu.:4.000
## Max. :60.00 Max. :1 Max. :2 Max. :6.000
##
## Nationality Number_Children Avg_RT Avg_Severity
## Min. :1.000 Min. :0.000 Min. : 561.8 Min. :2.000
## 1st Qu.:1.000 1st Qu.:0.000 1st Qu.:1130.2 1st Qu.:3.625
## Median :1.000 Median :1.000 Median :1277.1 Median :4.125
## Mean :1.188 Mean :1.272 Mean :1308.2 Mean :4.157
## 3rd Qu.:1.000 3rd Qu.:2.000 3rd Qu.:1431.8 3rd Qu.:4.713
## Max. :2.000 Max. :5.000 Max. :2851.5 Max. :6.870
##
## Failure Weight Time1 Time2
## Min. : 4.00 Min. :24 Min. : 1335 Min. : 57
## 1st Qu.:10.00 1st Qu.:24 1st Qu.: 31193 1st Qu.: 7258
## Median :13.00 Median :24 Median : 40637 Median : 13028
## Mean :12.49 Mean :24 Mean : 52142 Mean : 18762
## 3rd Qu.:14.00 3rd Qu.:24 3rd Qu.: 58730 3rd Qu.: 24241
## Max. :21.00 Max. :24 Max. :396979 Max. :172697
## NA's :5
## Vac1 Vac2 Vac Z.Vac
## Min. :1.000 Min. :1.000 Min. :-3.000 Min. :-1.0702
## 1st Qu.:4.000 1st Qu.:3.000 1st Qu.: 0.000 1st Qu.:-0.2930
## Median :6.000 Median :6.000 Median : 2.000 Median : 0.2251
## Mean :5.317 Mean :4.946 Mean : 1.131 Mean : 0.0000
## 3rd Qu.:7.000 3rd Qu.:7.000 3rd Qu.: 3.000 3rd Qu.: 0.4841
## Max. :7.000 Max. :7.000 Max. : 3.000 Max. : 0.4841
##
## Z.Avg_Severity Z.Avg_RTs
## Min. :-1.32556 Min. :-1.4150
## 1st Qu.:-0.32673 1st Qu.:-0.3374
## Median :-0.01939 Median :-0.0590
## Mean : 0.00000 Mean : 0.0000
## 3rd Qu.: 0.34191 3rd Qu.: 0.2344
## Max. : 1.66761 Max. : 2.9257
##

Now the standarized mean for these variables is 0, which means that the standarization worked.

**ATTITUDES TOWARDS VACCINATION (REDUCED SAMPLE)**

*# Histogram of Vaccination Attitudes (Both Conditions Together)*
**hist**(da$Vac,
 main="Attitudes Towards Vaccines",
 xlab="Vaccine Attitudes",
 ylab="Frequency",
 border="blue",
 col="green",
 prob = FALSE)


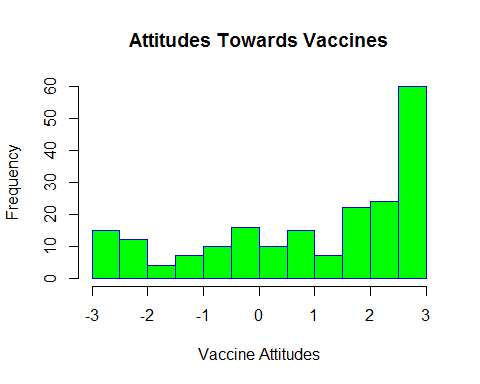


*# Summaries of attitudes towards vaccines for the entire sample*
**library**(plyr)
**describe**(da$Vac)

## vars n mean sd median trimmed mad min max range skew kurtosis
## X1 1 202 1.13 1.93 2 1.37 1.48 -3 3 6 -0.77 -0.66
## se
## X1 0.14

*# Histogram of attitudes towards vaccines splited by condition*
**library**(lattice)
**histogram**(~ Vac | Condition, main= "Attitudes Towards Vaccines", xlab="Score in the Scale", ylab="Frequency", col="grey", border="black",data=da)


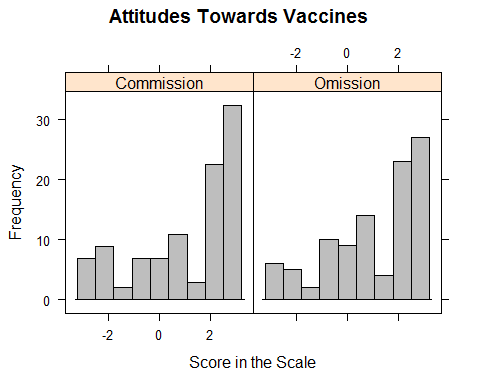


*# Summaries of attitudes towards vaccines per condition*
**library**(psych)
**describeBy**(da$Vac, da$Condition)

##
## Descriptive statistics by group
## group: Commission
## vars n mean sd median trimmed mad min max range skew kurtosis se
## X1 1 102 1.14 1.99 2 1.38 1.48 -3 3 6 -0.79 -0.73 0.2
## --------------------------------------------------------
## group: Omission
## vars n mean sd median trimmed mad min max range skew kurtosis
## X1 1 100 1.12 1.88 1.75 1.36 1.85 -3 3 6 -0.73 -0.64
## se
## X1 0.19

*# ANALYSES NOT INCLUDED IN THE SCRIPT FOR PRE-REGISTRATION:*

*# Histogram of attitudes towards vaccines splited by condition*
**library**(lattice)
**histogram**(~ Vac | Prescreening, main= "Attitudes Towards Vaccines", xlab="Score in the Scale", ylab="Frequency", col="green", border="blue", data=da)


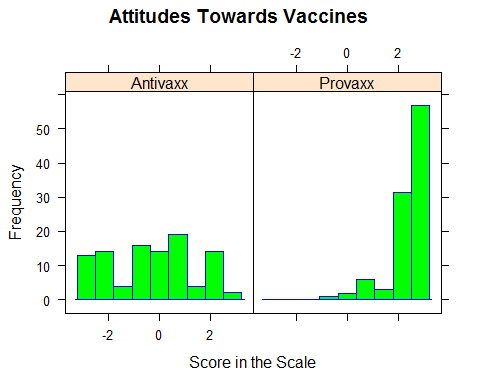


*# Summaries of attitudes towards vaccines per condition*
**library**(psych)
**describeBy**(da$Vac, da$Prescreening)

##
## Descriptive statistics by group
## group: Antivaxx
## vars n mean sd median trimmed mad min max range skew kurtosis
## X1 1 100 -0.29 1.72 0 -0.29 2.22 -3 3 6 -0.03 -1.04
## se
## X1 0.17
## --------------------------------------------------------
## group: Provaxx
## vars n mean sd median trimmed mad min max range skew kurtosis
## X1 1 102 2.52 0.74 3 2.69 0 -0.5 3 3.5 -1.91 3.6
## se
## X1 0.07

antivaxx<-da[ **which**(da$Prescreening == "Antivaxx"),]
provaxx<-da[ **which**(da$Prescreening == "Provaxx"),]

**histogram**(~ Vac | Condition, main= "Attitudes Towards Vaccines", xlab="Score in the Scale", ylab="Frequency", col="green", border="blue", data=antivaxx)


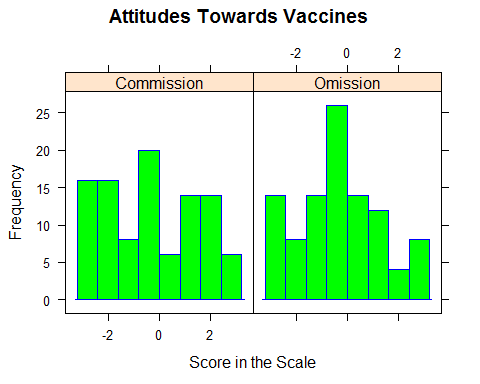


*# Summaries of attitudes towards vaccines per condition*
**library**(psych)
**describeBy**(antivaxx$Vac, antivaxx$Condition)

##
## Descriptive statistics by group
## group: Commission
## vars n mean sd median trimmed mad min max range skew kurtosis se
## X1 1 50 -0.29 1.85 0 -0.3 2.97 -3 3 6 0.02 -1.28 0.26
## --------------------------------------------------------
## group: Omission
## vars n mean sd median trimmed mad min max range skew kurtosis se
## X1 1 50 -0.29 1.6 0 -0.29 1.48 -3 2.5 5.5 -0.1 -0.83 0.23

**histogram**(~ Vac | Condition, main= "Attitudes Towards Vaccines", xlab="Score in the Scale", ylab="Frequency", col="green", border="blue", data=provaxx)


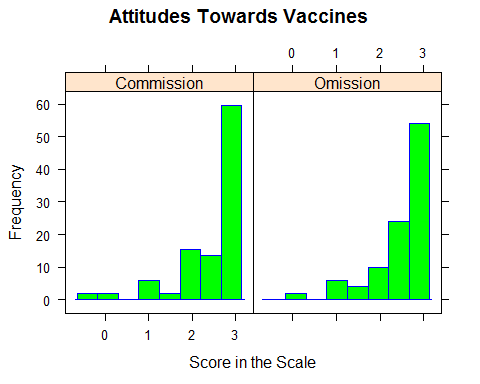


*# Summaries of attitudes towards vaccines per condition*
**library**(psych)
**describeBy**(provaxx$Vac, provaxx$Condition)

##
## Descriptive statistics by group
## group: Commission
## vars n mean sd median trimmed mad min max range skew kurtosis se
## X1 1 52 2.51 0.8 3 2.69 0 -0.5 3 3.5 -1.94 3.56 0.11
## --------------------------------------------------------
## group: Omission
## vars n mean sd median trimmed mad min max range skew kurtosis se
## X1 1 50 2.54 0.68 3 2.69 0 0 3 3 -1.74 2.71 0.1

control_model<-**lm**(Vac~1, data=antivaxx)
antivaxx_model<-**lm**(Vac~Condition, data=antivaxx)
**AIC**(control_model, antivaxx_model)

## df AIC
## control_model 2 395.3187
## antivaxx_model 3 397.3187

**library**(arm)

## Loading required package: MASS

## Loading required package: Matrix

## Loading required package: lme4

## Warning: package 'lme4' was built under R version 3.4.2

##
## arm (Version 1.9-3, built: 2016-11-21)

## Working directory is C:/Users/aj419/OneDrive - University of Exeter/Publications/In preparation/The Omission Bias

##
## Attaching package: 'arm'

## The following objects are masked from 'package:psych':
##
## logit, rescale, sim

**display**(antivaxx_model)

## lm(formula = Vac ~ Condition, data = antivaxx)
## coef.est coef.se
## (Intercept) -0.29 0.24
## ConditionOmission 0.00 0.35
## ---
## n = 100, k = 2
## residual sd = 1.73, R-Squared = 0.00

**sort**(da$Vac)

## [1] -3.0 -3.0 -3.0 -3.0 -3.0 -3.0 -3.0 -3.0 -3.0 -3.0 -3.0 -3.0 -3.0 -2.5
## [15] -2.5 -2.0 -2.0 -2.0 -2.0 -2.0 -2.0 -2.0 -2.0 -2.0 -2.0 -2.0 -2.0 -1.5
## [29] -1.5 -1.5 -1.5 -1.0 -1.0 -1.0 -1.0 -1.0 -1.0 -1.0 -0.5 -0.5 -0.5 -0.5
## [43] -0.5 -0.5 -0.5 -0.5 -0.5 -0.5 0.0 0.0 0.0 0.0 0.0 0.0 0.0 0.0
## [57] 0.0 0.0 0.0 0.0 0.0 0.0 0.0 0.0 0.5 0.5 0.5 0.5 0.5 0.5
## [71] 0.5 0.5 0.5 0.5 1.0 1.0 1.0 1.0 1.0 1.0 1.0 1.0 1.0 1.0
## [85] 1.0 1.0 1.0 1.0 1.0 1.5 1.5 1.5 1.5 1.5 1.5 1.5 2.0 2.0
## [99] 2.0 2.0 2.0 2.0 2.0 2.0 2.0 2.0 2.0 2.0 2.0 2.0 2.0 2.0
## [113] 2.0 2.0 2.0 2.0 2.0 2.0 2.5 2.5 2.5 2.5 2.5 2.5 2.5 2.5
## [127] 2.5 2.5 2.5 2.5 2.5 2.5 2.5 2.5 2.5 2.5 2.5 2.5 2.5 2.5
## [141] 2.5 2.5 3.0 3.0 3.0 3.0 3.0 3.0 3.0 3.0 3.0 3.0 3.0 3.0
## [155] 3.0 3.0 3.0 3.0 3.0 3.0 3.0 3.0 3.0 3.0 3.0 3.0 3.0 3.0
## [169] 3.0 3.0 3.0 3.0 3.0 3.0 3.0 3.0 3.0 3.0 3.0 3.0 3.0 3.0
## [183] 3.0 3.0 3.0 3.0 3.0 3.0 3.0 3.0 3.0 3.0 3.0 3.0 3.0 3.0
## [197] 3.0 3.0 3.0 3.0 3.0 3.0

**sum**(da$Vac > 0)/202

## [1] 0.6831683

**sum**(da$Vac > 2)/202

## [1] 0.4158416

**sum**(da$Vac<0)/202

## [1] 0.2376238

**sum**(da$Vac==0)/202

## [1] 0.07920792

Participants in the sample have in general a positive attitude towards vaccines (M=1.13, SD = 1.93) but their attitudes towards vaccine cover the entire scale from -3 to +3. The distribution of participants’ attitudes towards vaccines is very similar in both conditions (Comission: M=1.14, SD=1.99; Omission: M=1.12, SD=1.88). It is important to notice that although we selected equal number of participants with pro-vaccination and anti-vaccination attitudes in the pre-screening, a considerable proportion of these participants reported pro-vaccination attitudes in the experiment. Participants who reported pro-vaccination attitudes in the pre-screening showed very positive attitudes towards vaccines in the experiment (M=2.52, SD=0.74). In contrast, participants with anti-vaccination attitudes in the pre-screening reported very variable attitudes towards vaccines ranging from -3 to +3 (M=-0.29, SD=1.85). The dissagreement between the anti-vaccination attitudes reported in the pre-screening and the variable attitudes towards vaccines reported after the experiment does not seem to be driven by the experimental manipulations.

**MODELS PREDICTING SEVERITY RATINGS**

*# Exploring the distribution of the outcome variable*
**hist**(da$Avg_Severity,
 main="Histogram for Average Severity",
 xlab="Average Severity",
 border="blue",
 col="green")


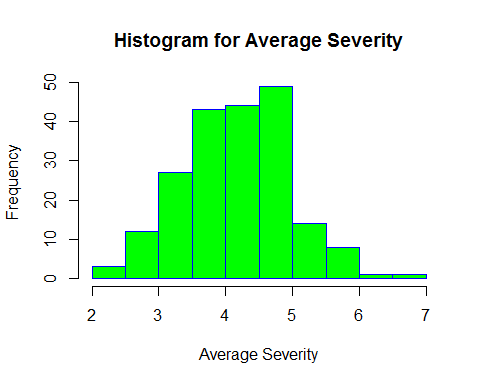


*# Summary for Average Severity ratings*
**library**(psych)
**describe**(da$Avg_Severity)

## vars n mean sd median trimmed mad min max range skew kurtosis
## X1 1 202 4.16 0.81 4.12 4.15 0.83 2 6.87 4.87 0.14 0.07
## se
## X1 0.06

The distribution of average severity ratings follows a normal distribution. The average severity ratings range from 2 to 6.87 (M=4.16, SD=0.81), which indicates that most of the average severity ratings were slighly above the middle of the scale.

*# Fixed intercept model*
model0.sev<-**lm**(Avg_Severity~1, data=da)
**library**(arm)
**display**(model0.sev)

## lm(formula = Avg_Severity ~ 1, data = da)
## coef.est coef.se
## (Intercept) 4.16 0.06
## ---
## n = 202, k = 1
## residual sd = 0.81, R-Squared = 0.00

**AIC**(model0.sev)

## [1] 492.8318

Using the mean as the only predictor has a poor fit to the data. The model explains less than 1% of the variance.

*# Selection of the Omission Condition as the reference category*
da <- **within**(da, Condition <- **relevel**(Condition, ref = 'Omission'))
*# Model to test H1a (omission bias hypothesis in severity ratings)*
*# Test for the predicted worse assessment of the symptoms/side effects in the comission condition than in the omission condition*
model1.sev<-**lm**(Avg_Severity~Condition,data=da)
*# Summary of model*
**library**(arm)
**display**(model1.sev)

## lm(formula = Avg_Severity ~ Condition, data = da)
## coef.est coef.se
## (Intercept) 4.15 0.08
## ConditionCommission 0.01 0.11
## ---
## n = 202, k = 2
## residual sd = 0.82, R-Squared = 0.00

**detach**("package:arm", unload=TRUE)
*# Coefficient Plot*
**library**(coefplot)

## Loading required package: ggplot2

## Warning: package 'ggplot2' was built under R version 3.4.4

##
## Attaching package: 'ggplot2'

## The following objects are masked from 'package:psych':
##
## %+%, alpha

**coefplot**(model1.sev)

## Warning: Ignoring unknown aesthetics: xmin, xmax


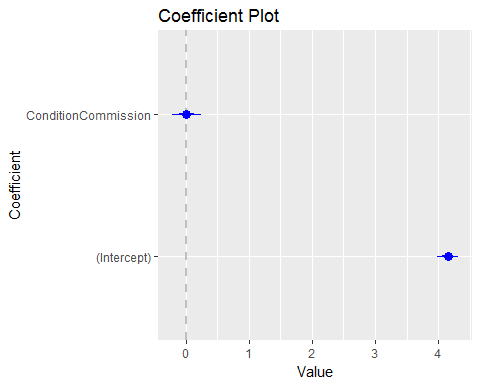


*# Graphical Representations of Model*
**library**(ggplot2)
 **ggplot**(da, **aes**(Condition, Avg_Severity)) +
 **geom_boxplot**()


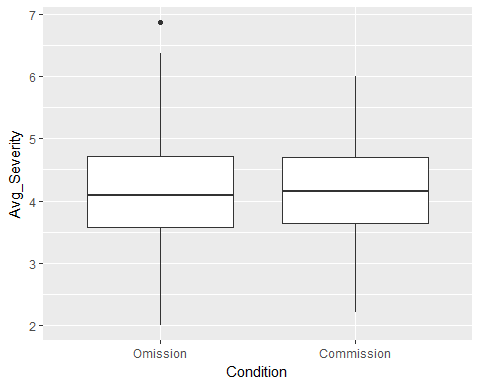


**ggplot**(da, **aes**(Condition, Avg_Severity)) +
 **geom_point**()


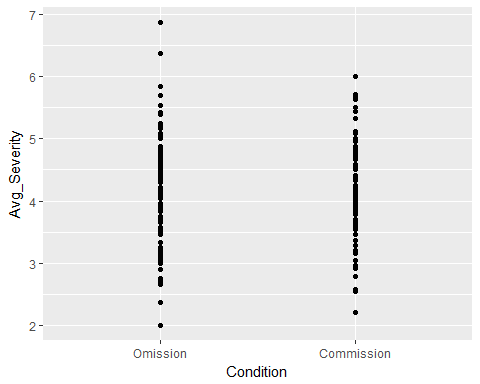


**library**(plyr)

*# means and sds by Condition*
**ddply**(da, .(Condition), summarize, Mean=**mean**(Avg_Severity), SD=**sd**(Avg_Severity))

## Condition Mean SD
## 1 Omission 4.151939 0.8585081
## 2 Commission 4.161064 0.7709408

*# Model comparisons*
**AIC**(model0.sev, model1.sev)

## df AIC
## model0.sev 2 492.8318
## model1.sev 3 494.8254

*# ANALYSES NOT REPORTED IN PREREGISTRATION*

**histogram**(~ Avg_Severity | Condition, main= "Attitudes Towards Vaccines", xlab="Score in the Scale", ylab="Frequency", col="grey", border="black", data=da)


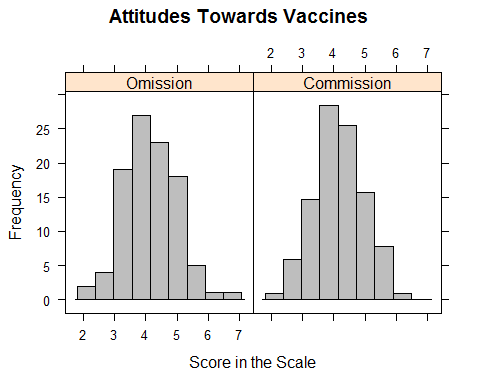


*# Summaries of attitudes towards vaccines per condition*
**library**(psych)
**describeBy**(da$Avg_Severity, da$Condition)

##
## Descriptive statistics by group
## group: Omission
## vars n mean sd median trimmed mad min max range skew kurtosis se
## X1 1 100 4.15 0.86 4.08 4.14 0.9 2 6.87 4.87 0.23 0.21 0.09
## --------------------------------------------------------
## group: Commission
## vars n mean sd median trimmed mad min max range skew kurtosis
## X1 1 102 4.16 0.77 4.15 4.15 0.78 2.21 6 3.79 0.03 -0.3
## se
## X1 0.08

Similarly to the null model, the first model explains less than 1% of the variance. This model (AIC=494.82) has a similar fit as the null model (AIC=492.83). The means of average severity ratings are very similar for both conditions (Comission: M=4.16, SD=0.77; Omission: M=4.15, SD=0.85).

*# Model to test H1b (congruity bias hypothesis)*
*# Test the predicted positive relationship between assessments of severity and provaccination attitudes in the omission condition and the negative relationship between these two variables in the comission condition*
model2.sev<-**lm**(Avg_Severity~Condition:Z.Vac,data=da)
*# Summaries*
**library**(arm)

##
## arm (Version 1.9-3, built: 2016-11-21)

## Working directory is C:/Users/aj419/OneDrive - University of Exeter/Publications/In preparation/The Omission Bias

##
## Attaching package: 'arm'

## The following objects are masked from 'package:coefplot':
##
## coefplot, coefplot.default

## The following objects are masked from 'package:psych':
##
## logit, rescale, sim

**display**(model2.sev)

## lm(formula = Avg_Severity ~ Condition:Z.Vac, data = da)
## coef.est coef.se
## (Intercept) 4.16 0.06
## ConditionOmission:Z.Vac -0.13 0.17
## ConditionCommission:Z.Vac -0.18 0.16
## ---
## n = 202, k = 3
## residual sd = 0.81, R-Squared = 0.01

**detach**("package:arm", unload=TRUE)
*# Coefficient Plot*
**coefplot**(model2.sev)

## Warning: Ignoring unknown aesthetics: xmin, xmax


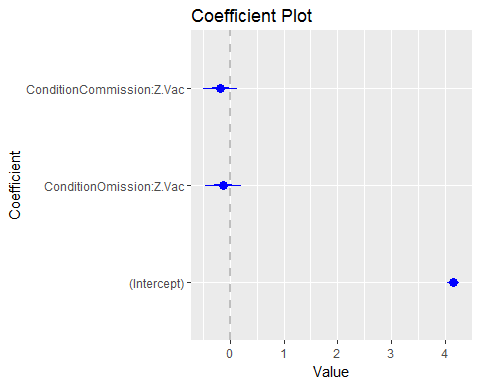


*# Model Comparisons*
**AIC**(model0.sev, model2.sev)

## df AIC
## model0.sev 2 492.8318
## model2.sev 4 494.9080

*# ANALYSES NOT REPORTED IN PREREGISTRATION*
Commission<-da[ **which**(da$Condition == "Commission"),]
Omission<-da[ **which**(da$Condition == "Omission"),]
**plot**(Avg_Severity~Vac, data=Commission, main="Comission Condition")


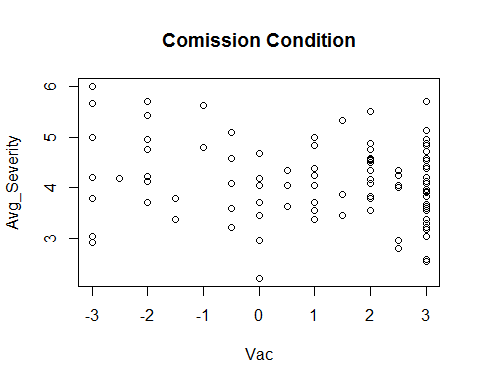


**plot**(Avg_Severity~Vac, data=Omission, main="Omission Condition")


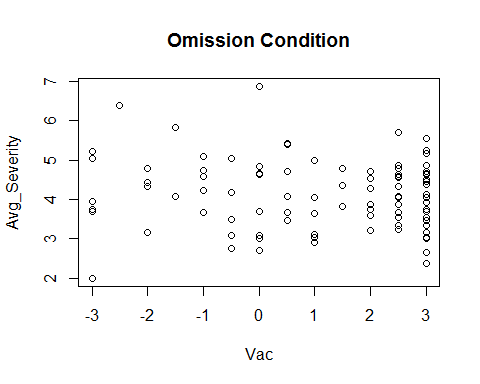
 The second model explains 1% of the variance. Its fit (AIC=494.90) is very similar to the fit of the null model (AIC=492.83). The scatterplots clearly show a lack of relationship between vaccination attitudes and average severity ratings in each condition.

*# Model to tests both hypotheses simulateneously*
model3.sev<-**lm**(Avg_Severity~Condition+Condition:Z.Vac,data=da)
*# Summaries*
**library**(arm)

##
## arm (Version 1.9-3, built: 2016-11-21)

## Working directory is C:/Users/aj419/OneDrive - University of Exeter/Publications/In preparation/The Omission Bias

##
## Attaching package: 'arm'

## The following objects are masked from 'package:coefplot':
##
## coefplot, coefplot.default

## The following objects are masked from 'package:psych':
##
## logit, rescale, sim

**display**(model3.sev)

## lm(formula = Avg_Severity ~ Condition + Condition:Z.Vac, data = da)
## coef.est coef.se
## (Intercept) 4.15 0.08
## ConditionCommission 0.01 0.11
## ConditionOmission:Z.Vac -0.13 0.17
## ConditionCommission:Z.Vac -0.18 0.16
## ---
## n = 202, k = 4
## residual sd = 0.82, R-Squared = 0.01

**detach**("package:arm", unload=TRUE)
*# Coefficient Plot*
**coefplot**(model3.sev)

## Warning: Ignoring unknown aesthetics: xmin, xmax


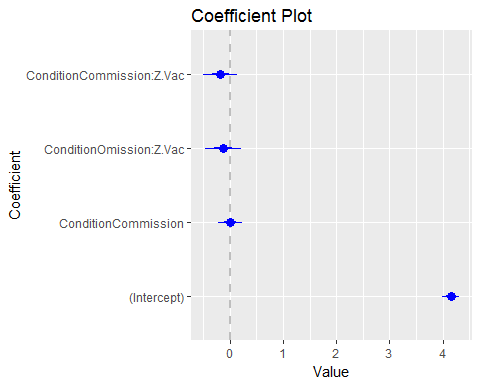


*# Graphical repressentation of the model*
**library**(ggplot2)
**ggplot**(da, **aes**(x=Z.Vac, y=Avg_Severity, color=Condition))+
 **geom_point**()+
 **geom_smooth**(method="lm", formula=y~x)+
 **facet_wrap**(~Condition)


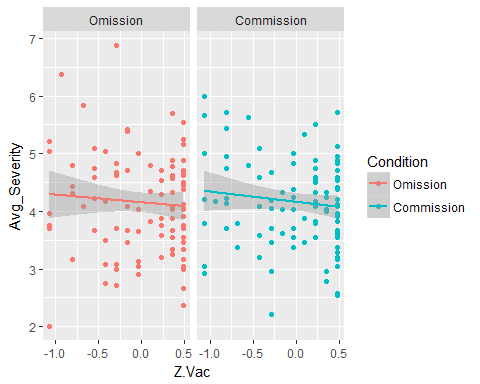


*# Model Comparisons*
**AIC**(model0.sev, model3.sev)

## df AIC
## model0.sev 2 492.8318
## model3.sev 5 496.9008

The third model explains 1% of the variance. Its fit (AIC=496.90) is worse than the fit of the null model (AIC=492.83)

*# MODELS NOT REPORTED IN PREREGISTRATION*
*# As the introduction of interaction terms without main effects in regressions is controversial, a model with all the main effects and the interaction was conducted.*
model4.sev<-**lm**(Avg_Severity~Condition*Z.Vac,data=da)
**library**(arm)

##
## arm (Version 1.9-3, built: 2016-11-21)

## Working directory is C:/Users/aj419/OneDrive - University of Exeter/Publications/In preparation/The Omission Bias

##
## Attaching package: 'arm'

## The following objects are masked from 'package:coefplot':
##
## coefplot, coefplot.default

## The following objects are masked from 'package:psych':
##
## logit, rescale, sim

**display**(model4.sev)

## lm(formula = Avg_Severity ~ Condition * Z.Vac, data = da)
## coef.est coef.se
## (Intercept) 4.15 0.08
## ConditionCommission 0.01 0.11
## Z.Vac -0.13 0.17
## ConditionCommission:Z.Vac -0.04 0.23
## ---
## n = 202, k = 4
## residual sd = 0.82, R-Squared = 0.01

**detach**("package:arm", unload=TRUE)
*# Coefficient Plot*
**coefplot**(model4.sev)

## Warning: Ignoring unknown aesthetics: xmin, xmax


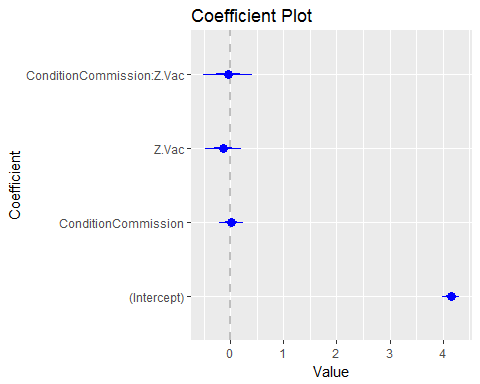
 The results were qualitatively similar to model 3.

*# Model comparisons*
**AIC**(model0.sev,model1.sev, model2.sev, model3.sev, model4.sev)

## df AIC
## model0.sev 2 492.8318
## model1.sev 3 494.8254
## model2.sev 4 494.9080
## model3.sev 5 496.9008
## model4.sev 5 496.9008

These results do not support the omission bias nor the congruity bias hypotheses in severity ratings.

**EXPLORATORY ANALYSES TO PREDICT AVERAGE SEVERITY RATINGS**

*# Standarization of Variables*
*# Standarization of level of education*
da$Z.Education<-(da$Education-**mean**(da$Education))/(2***sd**(da$Education))
*# Standarization of Age*
da$Z.Age<-(da$Age-**mean**(da$Age))/(2***sd**(da$Age))
*# Standarization of Number of Children*
da$Z.Number_Children<-(da$Number_Children-**mean**(da$Number_Children))/(2***sd**(da$Number_Children))

*# ANALYSES NOT INCLUDED IN PREREGISTRATION*
*# Recoding number of children into a dichotomous variable*
da$children<-**ifelse**(da$Number_Children==0, "nochildren", "withchildren")

*# ANALYSES NOT INCLUDED IN PREREGISTRATION*
*# Nationality Model*
da$nationality.factor<-**as.factor**(da$Nationality)
nationalitymodel<-**lm**(Avg_Severity~nationality.factor,data=da)
*# Education Model*
educationmodel<-**lm**(Avg_Severity~Z.Education, data=da)
*# Age Model*
agemodel<-**lm**(Avg_Severity~Z.Age, data=da)
*# Children models*
numberchildren<-**lm**(Avg_Severity~Z.Number_Children, data=da)
haschildren<-**lm**(Avg_Severity~children, data=da)
**AIC**(model0.sev, nationalitymodel, educationmodel, agemodel, numberchildren, haschildren)

## df AIC
## model0.sev 2 492.8318
## nationalitymodel 3 494.6734
## educationmodel 3 493.4037
## agemodel 3 494.2369
## numberchildren 3 494.8308
## haschildren 3 493.5429

None of these exploratory models improves the model fit in comparison to the null model.

*# ANALYSES NOT INCLUDED IN PREREGISTRATION*
*# Nationality Model*
nationalityxcondition<-**lm**(Avg_Severity~nationality.factor*Condition,data=da)
*# Education Model*
educationxcondition<-**lm**(Avg_Severity~Z.Education*Condition, data=da)
*# Age Model*
agexcondition<-**lm**(Avg_Severity~Z.Age*Condition, data=da)
*# Children models*
numberchildrenxcondition<-**lm**(Avg_Severity~Z.Number_Children*Condition, data=da)
haschildrenxcondition<-**lm**(Avg_Severity~children*Condition, data=da)
**AIC**(model0.sev, nationalityxcondition, educationxcondition, agexcondition,numberchildrenxcondition,haschildrenxcondition)

## df AIC
## model0.sev 2 492.8318
## nationalityxcondition 5 495.1156
## educationxcondition 5 497.1373
## agexcondition 5 498.1625
## numberchildrenxcondition 5 497.7745
## haschildrenxcondition 5 496.4157

None of these exploratory models improves the model fit in comparison to the null model.

*# ANALYSES NOT INCLUDED IN PREREGISTRATION*
*# Nationality Model*
nationalityxvac<-**lm**(Avg_Severity~nationality.factor*Z.Vac,data=da)
*# Education Model*
educationxvac<-**lm**(Avg_Severity~Z.Education*Z.Vac, data=da)
*# Age Model*
agexvac<-**lm**(Avg_Severity~Z.Age*Z.Vac, data=da)
*# Children models*
numberchildrenxvac<-**lm**(Avg_Severity~Z.Number_Children*Z.Vac, data=da)
haschildrenxvac<-**lm**(Avg_Severity~children*Z.Vac, data=da)
**AIC**(model0.sev, nationalityxvac,educationxvac,agexvac,numberchildrenxvac,haschildrenxvac)

## df AIC
## model0.sev 2 492.8318
## nationalityxvac 5 493.5827
## educationxvac 5 496.0055
## agexvac 5 495.0323
## numberchildrenxvac 5 496.3041
## haschildrenxvac 5 495.6764

Any of these exploratory models improves the model fit in comparison to the null model.

*# Nationality Model*
nationalitymodel<-**lm**(Avg_Severity~nationality.factor*Condition:Z.Vac,data=da)
*# Education Model*
educationmodel<-**lm**(Avg_Severity~Z.Education*Condition:Z.Vac, data=da)
*# Age Model*
agemodel<-**lm**(Avg_Severity~Z.Age*Condition:Z.Vac, data=da)
*# Children models*
numberchildren<-**lm**(Avg_Severity~Z.Number_Children*Condition:Z.Vac, data=da)
haschildren<-**lm**(Avg_Severity~children*Condition:Z.Vac, data=da)
**AIC**(model0.sev, nationalitymodel, educationmodel, agemodel, numberchildren, haschildren)

## df AIC
## model0.sev 2 492.8318
## nationalitymodel 7 492.2267
## educationmodel 7 499.6859
## agemodel 7 497.9736
## numberchildren 7 493.1269
## haschildren 7 494.6803

None of these exploratory models improves the model fit of the null model.

**MODELS TO PREDICT RECALL**

*# Standardizing Average Reaction Times*
da$Z.Avg_RTs<-(da$Avg_RT-**mean**(da$Avg_RT))/(2***sd**(da$Avg_RT))

*# Fixed logistic intercept model*
model0.Rec<-**glm**(**cbind**(Recall,Failure)~1, family="binomial",data=da)
*# Summary*
**library**(arm)

##
## arm (Version 1.9-3, built: 2016-11-21)

## Working directory is C:/Users/aj419/OneDrive - University of Exeter/Publications/In preparation/The Omission Bias

##
## Attaching package: 'arm'

## The following objects are masked from 'package:coefplot':
##
## coefplot, coefplot.default

## The following objects are masked from 'package:psych':
##
## logit, rescale, sim

**display**(model0.Rec)

## glm(formula = cbind(Recall, Failure) ~ 1, family = "binomial",
## data = da)
## coef.est coef.se
## (Intercept) -0.08 0.03
## ---
## n = 197, k = 1
## residual deviance = 321.6, null deviance = 321.6 (difference = 0.0)

*# Control models*
model1.Rec<-**glm**(**cbind**(Recall,Failure)~Z.Avg_RTs+Z.Avg_Severity, family = "binomial", data=da)
model2.Rec<-**glm**(**cbind**(Recall,Failure)~Z.Avg_RTs, family = "binomial", data=da)
model3.Rec<-**glm**(**cbind**(Recall,Failure)~Z.Avg_Severity, family = "binomial", data=da)
*# Model comparisons*
**AIC**(model0.Rec, model1.Rec, model2.Rec, model3.Rec)

## df AIC
## model0.Rec 1 1028.242
## model1.Rec 3 1026.910
## model2.Rec 2 1025.251
## model3.Rec 2 1029.908

*# Summary of the best fitting control model*
**library**(arm)
**display**(model2.Rec)

## glm(formula = cbind(Recall, Failure) ~ Z.Avg_RTs, family = "binomial",
## data = da)
## coef.est coef.se
## (Intercept) -0.08 0.03
## Z.Avg_RTs 0.13 0.06
## ---
## n = 197, k = 2
## residual deviance = 316.6, null deviance = 321.6 (difference = 5.0)

*# Coefficient Plot*
**detach**("package:arm",unload=TRUE)
**coefplot**(model2.Rec)

## Warning: Ignoring unknown aesthetics: xmin, xmax


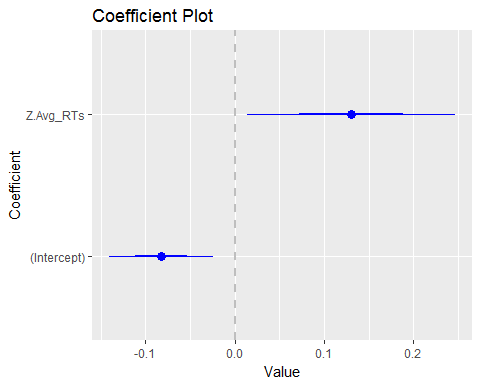
 The model with only average reaction time as a predictor (AIC=1025.25) is the only control model that has a better fit to the data than the null model (AIC = 1028.242). This model shows a positive relationship between average reaction time and proportion of symptoms/side effects correctly recalled (B=0.13, SE=0.06), which replicates previous recall experiments in which the greater the time to process words the better the recall of those words.

*# Models to test the omission bias hypothesis in recall (H2a)*
model4.Rec<-**glm**(**cbind**(Recall,Failure)~Condition, family="binomial",data=da)
model5.Rec<-**glm**(**cbind**(Recall,Failure)~Condition+Z.Avg_RTs, family="binomial",data=da)
*# Summary*
**library**(arm)

##
## arm (Version 1.9-3, built: 2016-11-21)

## Working directory is C:/Users/aj419/OneDrive - University of Exeter/Publications/In preparation/The Omission Bias

##
## Attaching package: 'arm'

## The following objects are masked from 'package:coefplot':
##
## coefplot, coefplot.default

## The following objects are masked from 'package:psych':
##
## logit, rescale, sim

**display**(model4.Rec)

## glm(formula = cbind(Recall, Failure) ~ Condition, family = "binomial",
## data = da)
## coef.est coef.se
## (Intercept) -0.05 0.04
## ConditionCommission -0.05 0.06
## ---
## n = 197, k = 2
## residual deviance = 320.8, null deviance = 321.6 (difference = 0.8)

**detach**("package:arm",unload=TRUE)
*# Coefficient Plot*
**coefplot**(model4.Rec)

## Warning: Ignoring unknown aesthetics: xmin, xmax


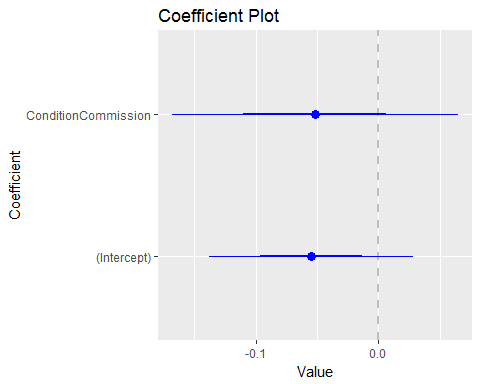


*# Summary*
**library**(arm)

##
## arm (Version 1.9-3, built: 2016-11-21)

## Working directory is C:/Users/aj419/OneDrive - University of Exeter/Publications/In preparation/The Omission Bias

##
## Attaching package: 'arm'

## The following objects are masked from 'package:coefplot':
##
## coefplot, coefplot.default

## The following objects are masked from 'package:psych':
##
## logit, rescale, sim

**display**(model5.Rec)

## glm(formula = cbind(Recall, Failure) ~ Condition + Z.Avg_RTs,
## family = "binomial", data = da)
## coef.est coef.se
## (Intercept) -0.05 0.04
## ConditionCommission -0.07 0.06
## Z.Avg_RTs 0.14 0.06
## ---
## n = 197, k = 3
## residual deviance = 315.3, null deviance = 321.6 (difference = 6.3)

*# Coefficient Plot*
**detach**("package:arm",unload=TRUE)
**coefplot**(model5.Rec)

## Warning: Ignoring unknown aesthetics: xmin, xmax


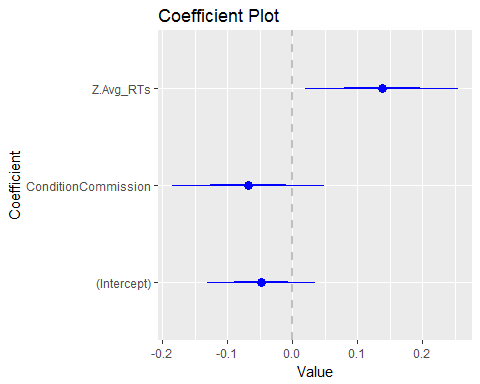


*# Model Comparisons*
**AIC**(model2.Rec, model4.Rec, model5.Rec)

## df AIC
## model2.Rec 2 1025.251
## model4.Rec 2 1029.452
## model5.Rec 3 1025.919

The condition model (AIC=1029.452) has a worse fit than the reaction time model (AIC=1025.251). The inclusion of condition in the reaction time model does not improve its fit (AIC=1025.919). These result do not support the omission bias in recall.

*# Models to Test the Congruity Bias Hypothesis in recall (H2b)*
model6.Rec<-**glm**(**cbind**(Recall, Failure)~Condition:Z.Vac, family="binomial", data=da)
model7.Rec<-**glm**(**cbind**(Recall, Failure)~Condition:Z.Vac+Z.Avg_RTs, family="binomial", data=da)
*# Summary*
**library**(arm)

##
## arm (Version 1.9-3, built: 2016-11-21)

## Working directory is C:/Users/aj419/OneDrive - University of Exeter/Publications/In preparation/The Omission Bias

##
## Attaching package: 'arm'

## The following objects are masked from 'package:coefplot':
##
## coefplot, coefplot.default

## The following objects are masked from 'package:psych':
##
## logit, rescale, sim

**display**(model6.Rec)

## glm(formula = cbind(Recall, Failure) ~ Condition:Z.Vac, family = "binomial",
## data = da)
## coef.est coef.se
## (Intercept) -0.08 0.03
## ConditionOmission:Z.Vac 0.17 0.09
## ConditionCommission:Z.Vac 0.12 0.08
## ---
## n = 197, k = 3
## residual deviance = 315.6, null deviance = 321.6 (difference = 6.0)

*# Coefficient Plot*
**detach**("package:arm", unload=TRUE)
**coefplot**(model6.Rec)

## Warning: Ignoring unknown aesthetics: xmin, xmax


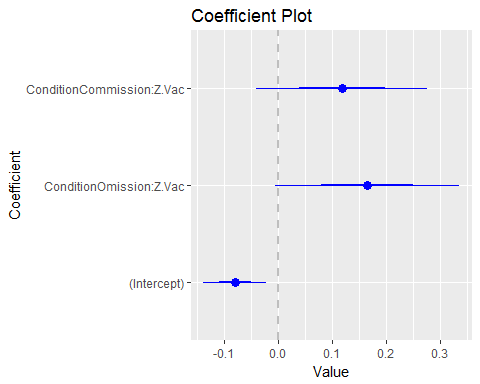


*# Summary*
**library**(arm)

##
## arm (Version 1.9-3, built: 2016-11-21)

## Working directory is C:/Users/aj419/OneDrive - University of Exeter/Publications/In preparation/The Omission Bias

##
## Attaching package: 'arm'

## The following objects are masked from 'package:coefplot':
##
## coefplot, coefplot.default

## The following objects are masked from 'package:psych':
##
## logit, rescale, sim

**display**(model7.Rec)

## glm(formula = cbind(Recall, Failure) ~ Condition:Z.Vac + Z.Avg_RTs,
## family = "binomial", data = da)
## coef.est coef.se
## (Intercept) -0.08 0.03
## Z.Avg_RTs 0.12 0.06
## ConditionOmission:Z.Vac 0.16 0.09
## ConditionCommission:Z.Vac 0.10 0.08
## ---
## n = 197, k = 4
## residual deviance = 311.4, null deviance = 321.6 (difference = 10.2)

*# Coefficient Plot*
**detach**("package:arm", unload=TRUE)
**coefplot**(model7.Rec)

## Warning: Ignoring unknown aesthetics: xmin, xmax


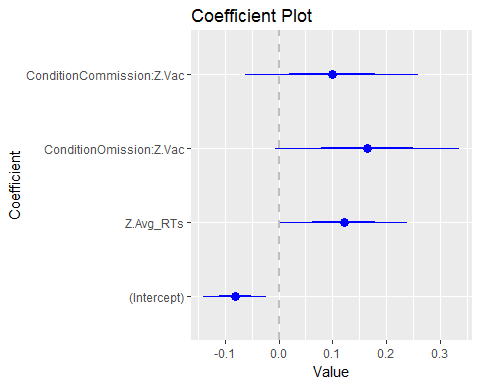


*#Model comparisons*
**AIC**(model2.Rec, model6.Rec, model7.Rec)

## df AIC
## model2.Rec 2 1025.251
## model6.Rec 3 1026.274
## model7.Rec 4 1024.026

The model with the interaction between condition and attitudes towards vaccination (AIC=1026.25) has a similar fit to the reaction time model (AIC=1025.251). The addition of the interaction between condition and attitudes towards vaccination to the reaction model does not improve its fit (AIC=1024.026). These results do not support the congruity bias hypothesis in recall.

*# Models to tests both hypotheses simultaneouusly*
model8.Rec<-**glm**(**cbind**(Recall,Failure)~Condition+Condition:Z.Vac, family=binomial, data=da)
model9.Rec<-**glm**(**cbind**(Recall,Failure)~Condition+Condition:Z.Vac+Z.Avg_RTs, family=binomial, data=da)

*# Summary*
**library**(arm)

##
## arm (Version 1.9-3, built: 2016-11-21)

## Working directory is C:/Users/aj419/OneDrive - University of Exeter/Publications/In preparation/The Omission Bias

##
## Attaching package: 'arm'

## The following objects are masked from 'package:coefplot':
##
## coefplot, coefplot.default

## The following objects are masked from 'package:psych':
##
## logit, rescale, sim

**display**(model8.Rec)

## glm(formula = cbind(Recall, Failure) ~ Condition + Condition:Z.Vac,
## family = binomial, data = da)
## coef.est coef.se
## (Intercept) -0.05 0.04
## ConditionCommission -0.05 0.06
## ConditionOmission:Z.Vac 0.17 0.09
## ConditionCommission:Z.Vac 0.12 0.08
## ---
## n = 197, k = 4
## residual deviance = 314.8, null deviance = 321.6 (difference = 6.8)

*# Coefficient Plot*
**detach**("package:arm", unload=TRUE)
**coefplot**(model8.Rec)

## Warning: Ignoring unknown aesthetics: xmin, xmax


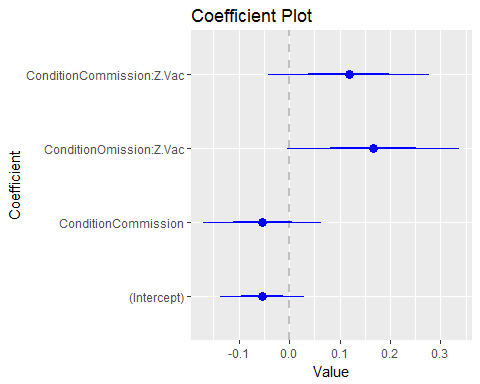


*# Summary*
**library**(arm)

##
## arm (Version 1.9-3, built: 2016-11-21)

## Working directory is C:/Users/aj419/OneDrive - University of Exeter/Publications/In preparation/The Omission Bias

##
## Attaching package: 'arm'

## The following objects are masked from 'package:coefplot':
##
## coefplot, coefplot.default

## The following objects are masked from 'package:psych':
##
## logit, rescale, sim

**display**(model9.Rec)

## glm(formula = cbind(Recall, Failure) ~ Condition + Condition:Z.Vac +
## Z.Avg_RTs, family = binomial, data = da)
## coef.est coef.se
## (Intercept) -0.05 0.04
## ConditionCommission -0.07 0.06
## Z.Avg_RTs 0.13 0.06
## ConditionOmission:Z.Vac 0.17 0.09
## ConditionCommission:Z.Vac 0.10 0.08
## ---
## n = 197, k = 5
## residual deviance = 310.1, null deviance = 321.6 (difference = 11.6)

*# Coefficient Plot*
**detach**("package:arm", unload=TRUE)
**coefplot**(model9.Rec)

## Warning: Ignoring unknown aesthetics: xmin, xmax


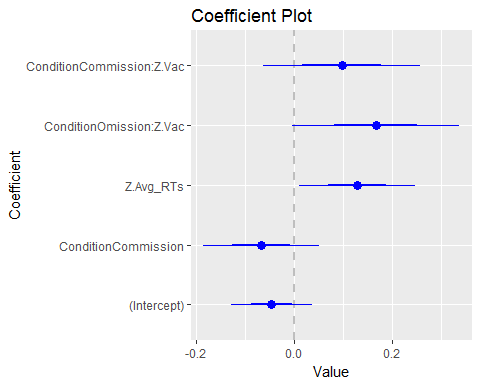


*# Model Comparisons*
**AIC**(model2.Rec, model7.Rec, model8.Rec, model9.Rec)

## df AIC
## model2.Rec 2 1025.251
## model7.Rec 4 1024.026
## model8.Rec 4 1027.447
## model9.Rec 5 1024.685

The reaction time model (AIC=1024), the model with reaction time and the interaction between condition and attitudes towards vaccination (AIC=1024.026) and the model with also the main effect of condition (AIC=1024.685) have a similar fit. The removal of reaction times from this last model worsens its model fit (AIC=1027.447). These results do not support either the omission bias or the congruity bias hypotheses in recall.

*#MODELS NOT INCLUDED IN PREREGISTRATION*
*#As the introduction of interaction terms without main effects is controversial, two models with all the main effects and the interaction term of interst were conducted*
model10.Rec<-**glm**(**cbind**(Recall,Failure)~Condition*Z.Vac, family=binomial, data=da)
model11.Rec<-**glm**(**cbind**(Recall,Failure)~Condition*Z.Vac+Z.Avg_RTs, family=binomial, data=da)

*# Summary*
**library**(arm)

##
## arm (Version 1.9-3, built: 2016-11-21)

## Working directory is C:/Users/aj419/OneDrive - University of Exeter/Publications/In preparation/The Omission Bias

##
## Attaching package: 'arm'

## The following objects are masked from 'package:coefplot':
##
## coefplot, coefplot.default

## The following objects are masked from 'package:psych':
##
## logit, rescale, sim

**display**(model10.Rec)

## glm(formula = cbind(Recall, Failure) ~ Condition * Z.Vac, family = binomial,
## data = da)
## coef.est coef.se
## (Intercept) -0.05 0.04
## ConditionCommission -0.05 0.06
## Z.Vac 0.17 0.09
## ConditionCommission:Z.Vac -0.05 0.12
## ---
## n = 197, k = 4
## residual deviance = 314.8, null deviance = 321.6 (difference = 6.8)

*# Coefficient Plot*
**detach**("package:arm", unload=TRUE)
**coefplot**(model10.Rec)

## Warning: Ignoring unknown aesthetics: xmin, xmax


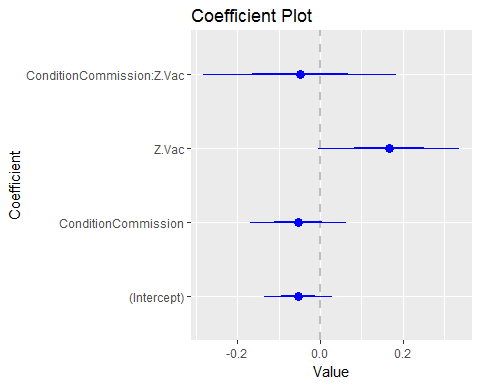


*# Summary*
**library**(arm)

##
## arm (Version 1.9-3, built: 2016-11-21)

## Working directory is C:/Users/aj419/OneDrive - University of Exeter/Publications/In preparation/The Omission Bias

##
## Attaching package: 'arm'

## The following objects are masked from 'package:coefplot':
##
## coefplot, coefplot.default

## The following objects are masked from 'package:psych':
##
## logit, rescale, sim

**display**(model11.Rec)

## glm(formula = cbind(Recall, Failure) ~ Condition * Z.Vac + Z.Avg_RTs,
## family = binomial, data = da)
## coef.est coef.se
## (Intercept) -0.05 0.04
## ConditionCommission -0.07 0.06
## Z.Vac 0.17 0.09
## Z.Avg_RTs 0.13 0.06
## ConditionCommission:Z.Vac -0.07 0.12
## ---
## n = 197, k = 5
## residual deviance = 310.1, null deviance = 321.6 (difference = 11.6)

*# Coefficient Plot*
**detach**("package:arm", unload=TRUE)
**coefplot**(model11.Rec)

## Warning: Ignoring unknown aesthetics: xmin, xmax


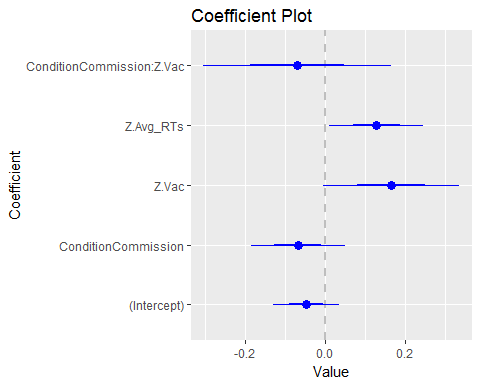


*# Model Comparisons*
**AIC**(model2.Rec, model4.Rec, model5.Rec, model10.Rec, model11.Rec)

## df AIC
## model2.Rec 2 1025.251
## model4.Rec 2 1029.452
## model5.Rec 3 1025.919
## model10.Rec 4 1027.447
## model11.Rec 5 1024.685

**EXPLORATORY ANALYSES**

*# ANALYSES NOT INCLUDED IN PREREGISTRATION*
*# Nationality Model*
da$nationality.factor<-**as.factor**(da$Nationality)
nationalitymodel<-**glm**(**cbind**(Recall,Failure)~nationality.factor,family="binomial",data=da)
*# Education Model*
educationmodel<-**glm**(**cbind**(Recall,Failure)~Z.Education, family="binomial",data=da)
*# Age Model*
agemodel<-**glm**(**cbind**(Recall,Failure)~Z.Age, family="binomial",data=da)
*# Children models*
numberchildren<-**glm**(**cbind**(Recall,Failure)~Z.Number_Children, family="binomial",data=da)
haschildren<-**glm**(**cbind**(Recall,Failure)~children, family="binomial", data=da)
**AIC**(model2.Rec, nationalitymodel, educationmodel, agemodel, numberchildren, haschildren)

## df AIC
## model2.Rec 2 1025.251
## nationalitymodel 2 1027.307
## educationmodel 2 1011.992
## agemodel 2 1028.312
## numberchildren 2 1030.224
## haschildren 2 1030.164

*# Nationality Model*
da$nationality.factor<-**as.factor**(da$Nationality)
nationalitymodel<-**glm**(**cbind**(Recall,Failure)~nationality.factor,family="binomial",data=da)
*# Education Model*
educationmodel<-**glm**(**cbind**(Recall,Failure)~Z.Education, family="binomial",data=da)
*# Age Model*
agemodel<-**glm**(**cbind**(Recall,Failure)~Z.Age, family="binomial",data=da)
*# Children models*
numberchildren<-**glm**(**cbind**(Recall,Failure)~Z.Number_Children, family="binomial",data=da)
haschildren<-**glm**(**cbind**(Recall,Failure)~children, family="binomial", data=da)
**AIC**(model2.Rec, nationalitymodel, educationmodel, agemodel, numberchildren, haschildren)

## df AIC
## model2.Rec 2 1025.251
## nationalitymodel 2 1027.307
## educationmodel 2 1011.992
## agemodel 2 1028.312
## numberchildren 2 1030.224
## haschildren 2 1030.164

The education model (AIC=1027.307) is the only model that has a better fit than the null model (AIC=1028.242)

**library**(arm)

##
## arm (Version 1.9-3, built: 2016-11-21)

## Working directory is C:/Users/aj419/OneDrive - University of Exeter/Publications/In preparation/The Omission Bias

##
## Attaching package: 'arm'

## The following objects are masked from 'package:coefplot':
##
## coefplot, coefplot.default

## The following objects are masked from 'package:psych':
##
## logit, rescale, sim

**display**(educationmodel)

## glm(formula = cbind(Recall, Failure) ~ Z.Education, family = "binomial",
## data = da)
## coef.est coef.se
## (Intercept) -0.08 0.03
## Z.Education 0.25 0.06
## ---
## n = 197, k = 2
## residual deviance = 303.4, null deviance = 321.6 (difference = 18.3)

**detach**("package:arm", unload=TRUE)
**library**(coefplot)
**coefplot**(educationmodel)

## Warning: Ignoring unknown aesthetics: xmin, xmax


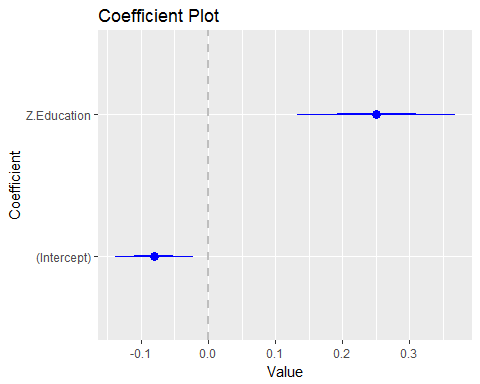
 The greater the education, the greater the recall (B=0.25, SE=0.06).

*# Nationality Model*
nationalitymodel.RT<-**glm**(**cbind**(Recall,Failure)~nationality.factor+Z.Avg_RTs,family="binomial",data=da)
*# Education Model*
educationmodel.RT<-**glm**(**cbind**(Recall,Failure)~Z.Education+Z.Avg_RTs, family="binomial",data=da)
*# Age Model*
agemodel.RT<-**glm**(**cbind**(Recall,Failure)~Z.Age+Z.Avg_RTs, family="binomial",data=da)
*# Children models*
numberchildren.RT<-**glm**(**cbind**(Recall,Failure)~Z.Number_Children+Z.Avg_RTs, family="binomial",data=da)
haschildren.RT<-**glm**(**cbind**(Recall,Failure)~children+Z.Avg_RTs, family="binomial", data=da)
**AIC**(model2.Rec, nationalitymodel.RT, educationmodel.RT, agemodel.RT, numberchildren.RT, haschildren.RT)

## df AIC
## model2.Rec 2 1025.251
## nationalitymodel.RT 3 1024.654
## educationmodel.RT 3 1011.805
## agemodel.RT 3 1023.492
## numberchildren.RT 3 1027.134
## haschildren.RT 3 1027.040

educationXRT<-**glm**(**cbind**(Recall,Failure)~Z.Education*Z.Avg_RTs, family="binomial",data=da)
**AIC**(educationmodel, educationmodel.RT, educationXRT)

## df AIC
## educationmodel 2 1011.992
## educationmodel.RT 3 1011.805
## educationXRT 4 1011.085

**coefplot**(educationmodel.RT)

## Warning: Ignoring unknown aesthetics: xmin, xmax


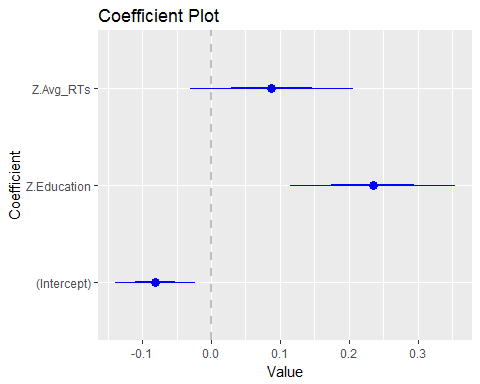


**coefplot**(educationXRT)

## Warning: Ignoring unknown aesthetics: xmin, xmax


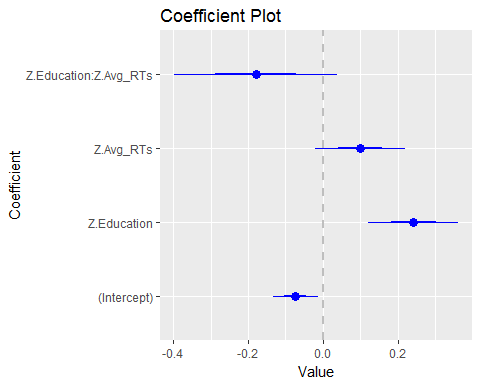


educationxcondition<-**glm**(**cbind**(Recall,Failure)~Z.Education*Condition, family="binomial",data=da)
**AIC**(educationxcondition, educationmodel.RT, educationmodel, model2.Rec)

## df AIC
## educationxcondition 4 1012.535
## educationmodel.RT 3 1011.805
## educationmodel 2 1011.992
## model2.Rec 2 1025.251

**coefplot**(educationxcondition)

## Warning: Ignoring unknown aesthetics: xmin, xmax


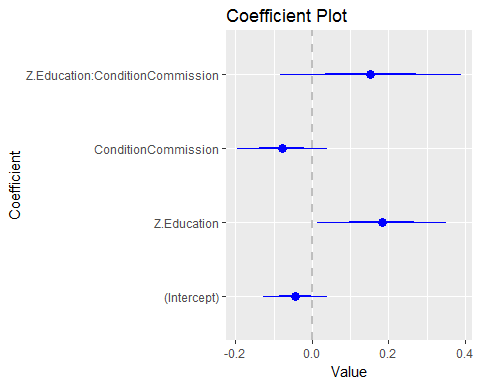


**library**(arm)

##
## arm (Version 1.9-3, built: 2016-11-21)

## Working directory is C:/Users/aj419/OneDrive - University of Exeter/Publications/In preparation/The Omission Bias

##
## Attaching package: 'arm'

## The following objects are masked from 'package:coefplot':
##
## coefplot, coefplot.default

## The following objects are masked from 'package:psych':
##
## logit, rescale, sim

**display**(educationmodel.RT)

## glm(formula = cbind(Recall, Failure) ~ Z.Education + Z.Avg_RTs,
## family = "binomial", data = da)
## coef.est coef.se
## (Intercept) -0.08 0.03
## Z.Education 0.23 0.06
## Z.Avg_RTs 0.09 0.06
## ---
## n = 197, k = 3
## residual deviance = 301.2, null deviance = 321.6 (difference = 20.4)

*# ANALYSES NOT INCLUDED IN PREREGISTRATION*
*# Nationality Model*
nationalityxcondition<-**lm**(Avg_Severity~nationality.factor*Condition,data=da)
*# Education Model*
educationxcondition<-**lm**(Avg_Severity~Z.Education*Condition, data=da)
*# Age Model*
agexcondition<-**lm**(Avg_Severity~Z.Age*Condition, data=da)
*# Children models*
numberchildrenxcondition<-**lm**(Avg_Severity~Z.Number_Children*Condition, data=da)
haschildrenxcondition<-**lm**(Avg_Severity~children*Condition, data=da)
**AIC**(model0.sev, nationalityxcondition, educationxcondition, agexcondition,numberchildrenxcondition,haschildrenxcondition)

## df AIC
## model0.sev 2 492.8318
## nationalityxcondition 5 495.1156
## educationxcondition 5 497.1373
## agexcondition 5 498.1625
## numberchildrenxcondition 5 497.7745
## haschildrenxcondition 5 496.4157

None of these exploratory models improves the model fit in comparison to the null model.

*# ANALYSES NOT INCLUDED IN PREREGISTRATION*
*# Nationality Model*
nationalityxvac<-**lm**(Avg_Severity~nationality.factor*Z.Vac,data=da)
*# Education Model*
educationxvac<-**lm**(Avg_Severity~Z.Education*Z.Vac, data=da)
*# Age Model*
agexvac<-**lm**(Avg_Severity~Z.Age*Z.Vac, data=da)
*# Children models*
numberchildrenxvac<-**lm**(Avg_Severity~Z.Number_Children*Z.Vac, data=da)
haschildrenxvac<-**lm**(Avg_Severity~children*Z.Vac, data=da)
**AIC**(model0.sev, nationalityxvac,educationxvac,agexvac,numberchildrenxvac,haschildrenxvac)

## df AIC
## model0.sev 2 492.8318
## nationalityxvac 5 493.5827
## educationxvac 5 496.0055
## agexvac 5 495.0323
## numberchildrenxvac 5 496.3041
## haschildrenxvac 5 495.6764

None of these exploratory models improves the model fit in comparison to the null model.

*# Nationality Model*
nationalitymodel<-**lm**(Avg_Severity~nationality.factor*Condition:Z.Vac,data=da)
*# Education Model*
educationmodel<-**lm**(Avg_Severity~Z.Education*Condition:Z.Vac, data=da)
*# Age Model*
agemodel<-**lm**(Avg_Severity~Z.Age*Condition:Z.Vac, data=da)
*# Children models*
numberchildren<-**lm**(Avg_Severity~Z.Number_Children*Condition:Z.Vac, data=da)
haschildren<-**lm**(Avg_Severity~children*Condition:Z.Vac, data=da)
**AIC**(model0.sev, nationalitymodel, educationmodel, agemodel, numberchildren, haschildren)

## df AIC
## model0.sev 2 492.8318
## nationalitymodel 7 492.2267
## educationmodel 7 499.6859
## agemodel 7 497.9736
## numberchildren 7 493.1269
## haschildren 7 494.6803

Any of these exploratory models improves the model fit of the null model.

*# Diagnostic plots of the model with the best fit*
**plot**(model7.Rec)


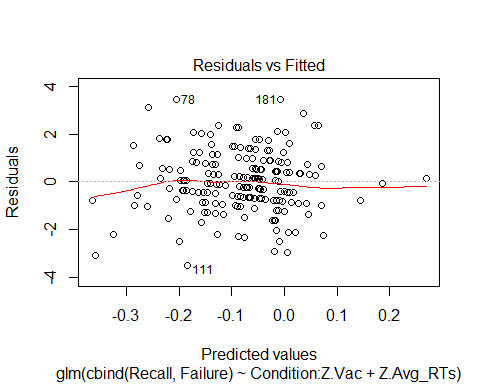

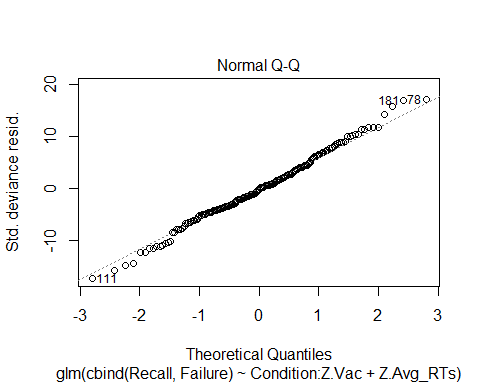

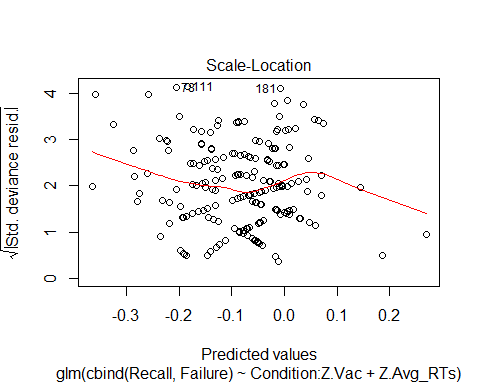

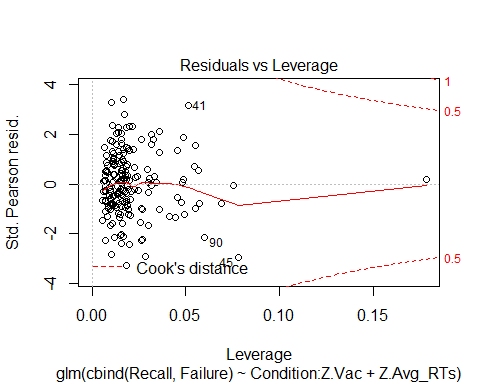


*# Assessing overdispersion*
residual_deviance<-311.4
degrees_of_freedom<-196
(dispersion_parameter<-residual_deviance/degrees_of_freedom)

## [1] 1.588776

The diagnostic plots look fine for a glm. The dispersion parameter is 1.58, which is greater than 1.5. Therefore, we have a slight overdispersion.

*# Refitting the model dealing with overdispersion (measuring the dispersion parameter, instead of assuming is 1)*
model7.Rec.quasi<-**glm**(**cbind**(Recall,Failure)~Condition:Z.Vac+Z.Avg_RTs, family=quasibinomial, data=da)
**library**(arm)
**display**(model7.Rec.quasi)

## glm(formula = cbind(Recall, Failure) ~ Condition:Z.Vac + Z.Avg_RTs,
## family = quasibinomial, data = da)
## coef.est coef.se
## (Intercept) -0.08 0.04
## Z.Avg_RTs 0.12 0.07
## ConditionOmission:Z.Vac 0.16 0.11
## ConditionCommission:Z.Vac 0.10 0.10
## ---
## n = 197, k = 4
## residual deviance = 311.4, null deviance = 321.6 (difference = 10.2)
## overdispersion parameter = 1.6

**detach**("package:arm", unload=TRUE)
**coefplot**(model7.Rec.quasi)

## Warning: Ignoring unknown aesthetics: xmin, xmax


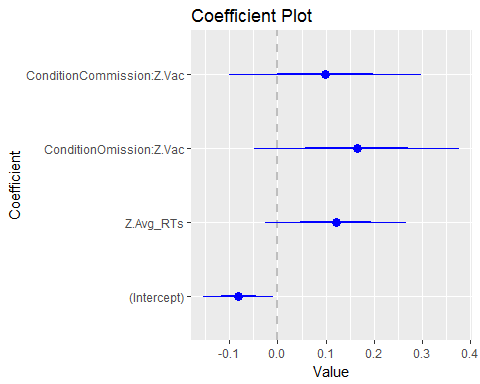
 The coefficients are the same but the SE are larger. The dispersio parameter is 1.6.

*# Effect Plots of the model*
**library**(effects)
**allEffects**(model7.Rec.quasi)

## model: cbind(Recall, Failure) ~ Condition:Z.Vac + Z.Avg_RTs
##
## Z.Avg_RTs effect
## Z.Avg_RTs
## -1 0 1 2
## 0.4493332 0.4793743 0.5095652 0.5396865
##
## Condition*Z.Vac effect
## Z.Vac
## Condition -1 -0.8 -0.6 -0.4 -0.2 0
## Omission 0.4390719 0.4471987 0.4553538 0.4635329 0.4717316 0.4799456
## Commission 0.4553934 0.4602904 0.4651950 0.4701064 0.4750236 0.4799456
## Z.Vac
## Condition 0.2 0.4
## Omission 0.4881705 0.4964018
## Commission 0.4848716 0.4898004

**plot**(**allEffects**(model7.Rec.quasi))


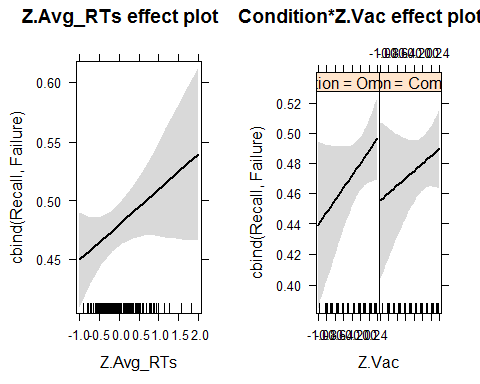


*# Probabilities*
e.out <- **allEffects**(model7.Rec.quasi)
e.out$Condition$model.matrix

## (Intercept) Z.Avg_RTs ConditionOmission:Z.Vac
## 1 1 0.007578583 -1.0
## 2 1 0.007578583 0.0
## 3 1 0.007578583 -0.8
## 4 1 0.007578583 0.0
## 5 1 0.007578583 -0.6
## 6 1 0.007578583 0.0
## 7 1 0.007578583 -0.4
## 8 1 0.007578583 0.0
## 9 1 0.007578583 -0.2
## 10 1 0.007578583 0.0
## 11 1 0.007578583 0.0
## 12 1 0.007578583 0.0
## 13 1 0.007578583 0.2
## 14 1 0.007578583 0.0
## 15 1 0.007578583 0.4
## 16 1 0.007578583 0.0
## ConditionCommission:Z.Vac
## 1 0.0
## 2 -1.0
## 3 0.0
## 4 -0.8
## 5 0.0
## 6 -0.6
## 7 0.0
## 8 -0.4
## 9 0.0
## 10 -0.2
## 11 0.0
## 12 0.0
## 13 0.0
## 14 0.2
## 15 0.0
## 16 0.4

*# Effect plot of Condition*
**plot**(**Effect**(focal.predictors = **c**("Condition"), mod = model7.Rec.quasi))


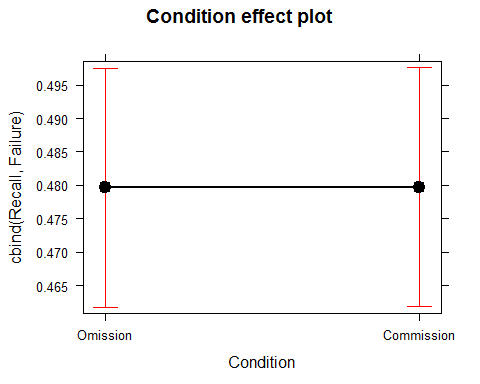


*# Effect plot of the Standarized Average Reaction Times*
**plot**(**Effect**(focal.predictors = **c**("Z.Avg_RTs"), mod = model7.Rec.quasi))


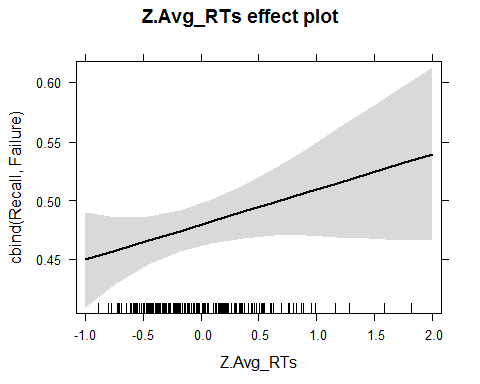


*# Effect plot of Condition * Standarized Attitudes towards Vaccines (Notice that the main effects of Condition are also included in the plot of the interaction effects. See file:///C:/Users/Rosa/Downloads/effect-displays-revised.pdf)*
**plot**(**Effect**(focal.predictors = **c**("Condition","Z.Vac"), mod = model7.Rec.quasi))


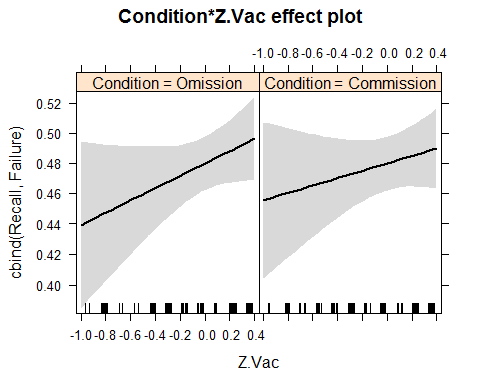


**RELIABILITY OF VACCINATON SCALE**

*# not reported in preregistration*
**library**(psy)

## Warning: package 'psy' was built under R version 3.4.1

##
## Attaching package: 'psy'

## The following object is masked from 'package:psych':
##
## wkappa

Vac<-**data.frame**(d$Vac1, d$Vac2)
**cronbach**(Vac)

## $sample.size
## [1] 261
##
## $number.of.items
## [1] 2
##
## $alpha
## [1] 0.9459489

**TABLES AND FIGURES FOR PAPER**

*# Table with model comparisons to predict severity ratings for the paper*
(summ.table <- **do.call**(rbind, **lapply**(**list**(model0.sev, model1.sev, model4.sev), broom::glance)))

## r.squared adj.r.squared sigma statistic p.value df logLik
## 1 0.000000e+00 0.000000000 0.8134446 NA NA 1 -244.4159
## 2 3.161551e-05 -0.004968226 0.8154628 0.006323302 0.9366992 2 -244.4127
## 3 9.513668e-03 -0.005493701 0.8156759 0.633933119 0.5939532 4 -243.4504
## AIC BIC deviance df.residual
## 1 492.8318 499.4483 133.0001 201
## 2 494.8254 504.7502 132.9959 200
## 3 496.9008 513.4422 131.7348 198

model.names<-**c**("Null","Condition", "Condition*Vaccination Attitudes")
reported.table <- bbmle::**AICtab**(model0.sev, model1.sev, model4.sev, weights = TRUE, sort = FALSE, mnames = model.names)
reported.table[["Resid. Dev"]] <- summ.table[["deviance"]] *# get the deviance from broom'd table*
reported.table

## dAIC df weight Resid. Dev
## Null 0.0 2 0.667 133.0
## Condition 2.0 3 0.246 133.0
## Condition*Vaccination Attitudes 4.1 5 0.087 131.7

reported.table <- bbmle::**AICtab**(model0.sev, model1.sev, model4.sev, sort = FALSE, mnames = model.names)
reported.table[["Resid. Dev"]] <- summ.table[["deviance"]] *# get the deviance from broom'd table*
reported.table

## dAIC df Resid. Dev
## Null 0.0 2 133.0
## Condition 2.0 3 133.0
## Condition*Vaccination Attitudes 4.1 5 131.7

*# Graphical Representations of model predicting severity ratigs for the paper*
**library**(ggplot2)

box_severity<-
 **ggplot**(da, **aes**(Condition, Avg_Severity)) +
 **geom_boxplot**()+
 **theme**(strip.text.x = **element_text**(color="black", size=20,face="bold" ))+
 **ylab** ("Average Severity Ratings")+
 **xlab** ("Condition") +
 **theme** (axis.title.y = **element_text**(color="black", size=20, face="bold"), axis.title.x = **element_text**(color="black", size=20, face="bold"))+**theme_bw**()+ **theme**(panel.grid.major = **element_blank**(), panel.grid.minor = **element_blank**()) + **expand_limits**(y=1)+**scale_y_continuous**(breaks=**seq**(1,7,1))

**library**(ggplot2)

line_severity<-**ggplot**(da, **aes**(x=Vac, y=Avg_Severity, color=Condition))+
 **geom_point**()+
 **geom_smooth**(method="lm", formula=y~x) +
 **theme**(strip.text.x = **element_text**(color="black", size=20,face="bold" ))+
 **ylab** ("Average Severity Ratings")+
 **xlab** ("Vaccination Attitudes") +
 **theme** (axis.title.y = **element_text**(color="black", size=20, face="bold"), axis.title.x = **element_text**(color="black", size=20, face="bold"))+**theme_bw**()+ **theme**(panel.grid.major = **element_blank**(), panel.grid.minor = **element_blank**())+ **expand_limits**(y=1)+**scale_y_continuous**(breaks=**seq**(1,7,1))+**theme**(legend.position = **c**(0.3, 0.15))

**library**(gridExtra)

## Warning: package 'gridExtra' was built under R version 3.4.4

**grid.arrange**(box_severity, line_severity, ncol=2)


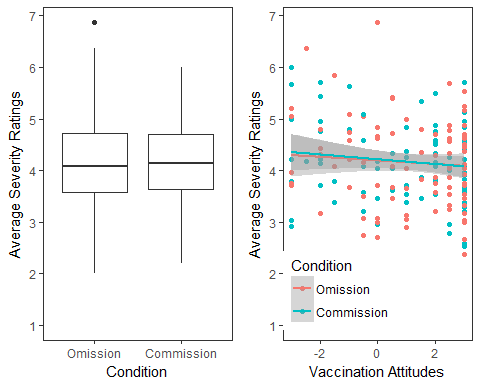


*# Table for the paper (model comparisons to predict recall)*
(summ.table <- **do.call**(rbind, **lapply**(**list**(model0.Rec,model2.Rec, model4.Rec, model5.Rec, model10.Rec, model11.Rec), broom::glance)))

## null.deviance df.null logLik AIC BIC deviance df.residual
## 1 321.615 196 -513.1211 1028.242 1031.525 321.6150 196
## 2 321.615 196 -510.6254 1025.251 1031.817 316.6236 195
## 3 321.615 196 -512.7258 1029.452 1036.018 320.8245 195
## 4 321.615 196 -509.9596 1025.919 1035.769 315.2920 194
## 5 321.615 196 -509.7237 1027.447 1040.580 314.8202 193
## 6 321.615 196 -507.3426 1024.685 1041.101 310.0581 192

model.names<-**c**("Null","Reaction Time", "Condition", "Condition + Reaction Time", "Condition*Vaccination Attitudes", "Condition*Vaccination Attitudes + Reaction Time")
reported.table <- bbmle::**AICtab**(model0.Rec,model2.Rec, model4.Rec, model5.Rec, model10.Rec, model11.Rec, weights = TRUE, sort = FALSE, mnames = model.names)
reported.table[["Resid. Dev"]] <- summ.table[["deviance"]] *# get the deviance from broom'd table*
reported.table

## dAIC df weight Resid. Dev
## Null 3.6 1 0.060 321.6
## Reaction Time 0.6 2 0.269 316.6
## Condition 4.8 2 0.033 320.8
## Condition + Reaction Time 1.2 3 0.192 315.3
## Condition*Vaccination Attitudes 2.8 4 0.090 314.8
## Condition*Vaccination Attitudes + Reaction Time 0.0 5 0.356 310.1

*# Graphical repressentation of the model to predict recall*
**library**(ggplot2)
**ggplot**(da, **aes**(x=Vac, y=Recall/24, color=Condition))+
 **geom_point**()+
 **geom_smooth**(method="lm", formula=y~x)+
 **facet_wrap**(~Condition) +
 **theme**(strip.text.x = **element_text**(color="black", size=14,face="bold" ))+
 **ylab** ("Proportion of Symptoms/Side Effects Correctly Recalled")+
 **xlab** ("Vaccination Attitudes") +
 **theme** (axis.title.y = **element_text**(color="black", size=12, face="bold"), axis.title.x = **element_text**(color="black", size=12, face="bold"))

## Warning: Removed 5 rows containing non-finite values (stat_smooth).

## Warning: Removed 5 rows containing missing values (geom_point).


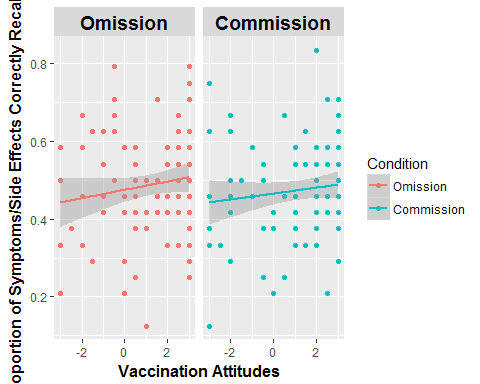


**library**(ggplot2)
box_recall<- **ggplot**(da, **aes**(Condition, Recall/24)) +
 **geom_boxplot**()+
 **theme**(strip.text.x = **element_text**(color="black", size=20,face="bold" ))+
 **ylab** ("Proportion of Correct Recall")+
 **xlab** ("Condition") +
 **theme** (axis.title.y = **element_text**(color="black", size=20, face="bold"), axis.title.x = **element_text**(color="black", size=20, face="bold"))+**theme_bw**()+ **theme**(panel.grid.major = **element_blank**(), panel.grid.minor = **element_blank**())+ **expand_limits**(y=0)+**scale_y_continuous**(breaks=**seq**(0,1,0.1))
*# Graphical repressentation of the model*
**library**(ggplot2)
line_recall<-**ggplot**(da, **aes**(x=Vac, y=Recall/24, color=Condition))+
 **geom_point**()+
 **geom_smooth**(method="lm", formula=y~x)+
 **theme**(strip.text.x = **element_text**(color="black", size=20,face="bold" ))+
 **ylab** ("Proportion of Correct Recall")+
 **xlab** ("Vaccination Attitudes") +
 **theme** (axis.title.y = **element_text**(color="black", size=20, face="bold"), axis.title.x = **element_text**(color="black", size=20, face="bold"))+**theme_bw**()+ **theme**(panel.grid.major = **element_blank**(), panel.grid.minor = **element_blank**())+ **expand_limits**(y=0)+**scale_y_continuous**(breaks=**seq**(0,1,0.1))+**theme**(legend.position = **c**(0.3, 0.15))

**library**(gridExtra)
**grid.arrange**(box_recall, line_recall, ncol=2)

## Warning: Removed 5 rows containing non-finite values (stat_boxplot).

## Warning: Removed 5 rows containing non-finite values (stat_smooth).

## Warning: Removed 5 rows containing missing values (geom_point).


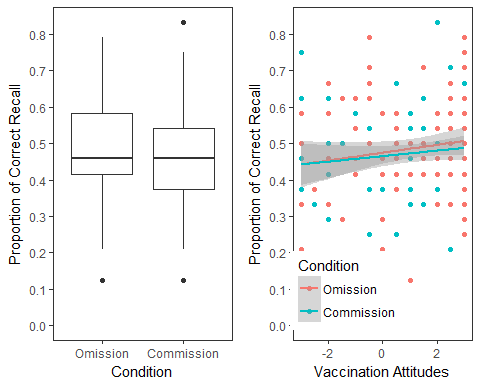


# F: REFERENCES

Asch, D. A., Baron, J., Hershey, J. C., Kunreuther, H., Meszaros, J., Ritov, I., & Spranca, M. (1994). Omission bias and pertussis vaccination. *Medical Decision Making, 14*(2), 118-123.

Boyer, P., & Parren, N. (2015). Threat-Related Information Suggests Competence: A Possible Factor in the Spread of Rumors. *PLoS ONE, 10*(6), e0128421. doi:10.1371/journal.pone.0128421

Brown, K. F., Kroll, J. S., Hudson, M. J., Ramsay, M., Green, J., Vincent, C. A., . . . Sevdalis, N. (2010). Omission bias and vaccine rejection by parents of healthy children: implications for the influenza A/H1N1 vaccination programme. *Vaccine, 28*(25), 4181-4185. doi:10.1016/j.vaccine.2010.04.012

Browne, M., Thomson, P., Rockloff, M. J., & Pennycook, G. (2015). Going against the Herd: Psychological and Cultural Factors Underlying the ‘Vaccination Confidence Gap’. *PLoS ONE, 10*(9), e0132562. doi:10.1371/journal.pone.0132562

Chandler, J. J., & Paolacci, G. (2017). Lie for a Dime. *Social Psychological and Personality Science*, 1948550617698203. doi:10.1177/1948550617698203

Clifford, S., & Wendell, D. G. (2016). How Disgust Influences Health Purity Attitudes. *Political Behavior, 38*(1), 155-178. doi:10.1007/s11109-015-9310-z

Connolly, T., & Reb, J. (2003). Omission bias in vaccination decisions: Where's the "omission"? Where's the "bias"? *Organizational Behavior and Human Decision Processes, 91*(2), 186-202. doi:10.1016/s0749-5978(03)00057-8

Eriksson, K., & Coultas, J. C. (2014). Corpses, Maggots, Poodles and Rats: Emotional Selection Operating in Three Phases of Cultural Transmission of Urban Legends. *Journal of Cognition and Culture, 14*(1-2), 1-26. doi::10.1163/15685373-12342107

Eriksson, K., Coultas, J. C., & de Barra, M. (2016). Cross-Cultural Differences in Emotional Selection on Transmission of Information. *Journal of Cognition and Culture, 16*(1-2), 122-143. doi::10.1163/15685373-12342171

Gelman (2008). Scaling regression inputs by dividing by two standard deviations. *Statist. Med.* 2008; 27:2865-2873. DOI: 10.1002/sim.3107

Gust, D. A., Strine, T. W., Maurice, E., Smith, P., Yusuf, H., Wilkinson, M., . . . Schwartz, B. (2004). Underimmunization Among Children: Effects of Vaccine Safety Concerns on Immunization Status. *Pediatrics, 114*(1), e16-e22. doi:10.1542/peds.114.1.e16

Heath, C., Bell, C., & Sternberg, E. (2001). Emotional selection in memes: the case of urban legends. *Journal of Personality & Social Psychology, 81*(6), 1028-1041.

Kahan, D. M., & Hilgard, J. (2016). The impact of pathogen-disgust sensitivity on vaccine and GM food risk perceptions: Some evidence for skepticism.

Miton, H., & Mercier, H. (2015). Cognitive obstacles to pro-vaccination beliefs. *Trends in Cognitive Sciences, 19*(11), 633-636. doi:10.1016/j.tics.2015.08.007

Peer, E., Samat, S., Brandimarte, L., & Acquisti, A. (2016). Beyond the Turk: an empirical comparison of alternative platforms for crowdsourcing online behavioral research. *SSRN*. doi:10.2139/ssrn.2594183

Ritov, I., & Baron, J. (1990). Reluctance to Vaccinate: Omission Bias and Ambiguity. *Journal of Behavioral Decision Making*(3), 168-186.

Spranca, M., Minsk, E., & Baron, J. (1991). Omission and commission in judgment and choice. *Journal of Experimental Social Psychology, 27*(1), 76-105. doi:10.1016/0022-1031(91)90011-T

Stubbersfield, J. M., Tehrani, J. J., & Flynn, E. G. (2017). Chicken Tumours and a Fishy Revenge: Evidence for Emotional Content Bias in the Cumulative Recall of Urban Legends. *Journal of Cognition and Culture, 17*(1-2), 12-26. doi:10.1163/15685373-12342189
