# Supplementary material for: No evidence that omission and confirmation biases affect the perception and recall of vaccine-related information
Source: PLoS One. 2020 Mar 4;15(3):e0228898. doi: 10.1371/journal.pone.0228898 (PMC7055885; doi:10.1371/journal.pone.0228898)
Supplement: Attachment — Submitted filename: Review.docx [file pone.0228898.s005.docx]

The manuscript investigates the effect of omission bias and confirmation bias on memorization of vaccine related information. The methodology is sound, and the research questions are interesting. The authors found results that do not confirm their hypotheses, but it doesn’t make their contribution less important.

I have some comments, most of them require only minor modifications but maybe a little more work is needed for the conclusion.

**L210:**

On the influence of vaccination attitudes on the memorization of vaccine related information Altay & Mercier 2018 tested the effect of “attitudinality” on memorization, and found that information perceived as being in favor of vaccination were not better memorized by participants in favor of vaccination than information perceived as being against vaccination (Experiment 2).

**L317:**

I would put figure 1 in supplementary materials and just give a description of the scale in one or two lines.

**Section 3.2.**

I do not find the statistical analyses very intuitive but it’s probably because I’m not familiar with model comparisons using AIC in experimental designs. If other reviewers or the editor do not find the analyses easy to understand either, effort should be made by the authors to clarify this section.

**Section 3.3.**

Since participants contributed to multiple data points (if I understood well), participants need to be added as a random factor in the logistic regression to account for the non-independence of the paired data (even though it will probably not change the results).

**L451:**

Minor comment: The critic on the need to do correction for multiple comparison is sound, but I don’t think using Bonferroni is, the test being too conservative (it increases too much the chances of making false negative). Would we find the same results with holm correction technique?

**Conclusion:**

I agree with the following sentence “Instead, the messages transmitted by people with anti-vaccination beliefs might spread due to having particular characteristics (e.g. being simple, concrete, emotional, unexpected, narrative, etc.) that make them especially “sticky” in human minds (Heath & Heath, 2008)”.

But I don’t see how it can explain anti-vaccination attitudes. At some point people need to find these messages convincing to have anti-vaccination attitudes. However, it seems that most people need not be convinced that vaccines are bad but rather that they are good.

This is why I find the following sentence not very convincing (L497-499):

“The lack of convincing evidence for the omission bias suggest that the spread of anti-vaccination messages might not be explained by the existence of a universal cognitive bias acting against the practice of vaccination (e.g. Miton & Mercier, 2015)”

Omission bias is surely a very bad candidate to explain anti-vaccination attitudes (and the authors made a convincing point) but the disgust hypothesis is not completely ruled out, even though it lacks empirical support. But many other properties of the human mind (and of our current environment) could explain resistance to vaccination. The authors only ruled out omission bias, not all the others possible cognitive bias.

The two explanations are compatible and probably true: vaccination can be counter-intuitive and pro-vaccination messages could be also less attractive than anti-vaccination messages.

As a side note, I tested the second hypothesis during my master thesis (in French unfortunately: YESILALTAY, S. (2018). ÉTUDE DES DÉTERMINANTS COGNITIFS DES ATTITUDES VACCINALES.) and found that vaccines descriptions containing disgust-related information (but not threat-related information) were better memorized than valence-neutral information (Experiment 2). Which supports the hypothesis made by the authors in the conclusion. The vignettes looked like that:

Disgust : Poliomyelitis (Polio) is a highly contagious and infectious viral disease that usually spreads from person to person through infected fecal matter entering the mouth.
Control : Poliomyelitis (Polio) is a contagious viral disease that can spread from person to person by oral transmission like eating infected food or drinking infected water.

Threat : As long as a single individual remains infected, children and adults worldwide are at risk of contracting polio.
Control : Nowadays, polio can only be found in remote areas of Afghanistan, Nigeria, and Pakistan.

**Footnote 1:**

Replace

“The evidence linking disgust sensibility with the formation of anti-vaccination attitudes is also rather week.”

by

“The evidence linking disgust sensibility with the formation of anti-vaccination attitudes is also rather *weak*.”

**L502-504:**

The last sentence of the article raises an important point but references to the literature on vaccine communication are missing, e.g.

Mckinnon, M., & Orthia, L. A. (2017). Vaccination communication strategies : What have we learned , and lost , in 200 years ?, *16*(03), 1–16.

Bruine de Bruin, W., Wallin, A., Parker, A. M., Strough, J., & Hanmer, J. (2017). Effects of anti-versus pro-vaccine narratives on responses by recipients varying in numeracy: A cross-sectional survey-based experiment. Medical Decision Making, 37(8), 860-870.

Nyhan, B., Reifler, J., Richey, S., & Freed, G. L. (2014). Effective Messages in Vaccine Promotion: A Randomized Trial. *Pediatrics*, *133*(4), e835–e842. https://doi.org/10.1542/peds.2013-2365

And also probably:

Aarøe, L., & Petersen, M. B. (2018). Cognitive Biases and Communication Strength in Social Networks : The Case of Episodic Frames, 1–21. https://doi.org/10.1017/S0007123418000273
