# Supplementary material for: No evidence that omission and confirmation biases affect the perception and recall of vaccine-related information
Source: PLoS One. 2020 Mar 4;15(3):e0228898. doi: 10.1371/journal.pone.0228898 (PMC7055885; doi:10.1371/journal.pone.0228898)
Supplement: Attachment — Submitted filename: Response to Reviewers.docx [file pone.0228898.s006.docx]

RESPONSE TO REVIEWERS

We would like to thank you for your time and effort dedicated to reviewing our manuscript. Your comments and suggestions are very much appreciated. We have made several modifications based on your comments, which we believe have improved the article.

In the following, you will find our responses for each of your comments.

# # EDITOR

**R1 made some suggestions of papers on vaccine attitudes which you might wish to cite. I’d encourage you to have a look at these, though I’ll leave it up to you which ones you feel would really add to the paper. If R1 is right, however, the suggestion they make regarding a tweak to line 210 would be particularly relevant, as it would link to something directly relevant to your study.**

R1 is right: the study does not provide evidence to rule out the disgust-antivaccination link. Consequently, we have changed the sentence to be more specific to the omission bias: “the lack of convincing evidence for the omission bias suggest that the spread of anti-vaccination messages might not be explained by an omission bias acting against the practice of vaccination” (lines 510-512). We do not comment on the disgust-antivaccination link, as this is not the topic of our article.

We have also added one of the suggested references on vaccine communication. We don’t think we need to specifically address the literature on vaccine communication as the article focuses on cognitive biases, not in specific interventions to make pro-vaccination messages more appealing.

**Analysis:
R1 reported having some difficulty following your results section. I think it might be worth (if possible) adding a citation to similar analyses/guides to such analyses for readers not familiar with the AIC approach. In particular, I think the use of the word ‘logistic’ threw R1 off the trail a bit: it seems they believed you had tested a yes/no of whether each ppt recalled each item, rather than testing proportions recalled correctly (I went to confirm what you’d done by looking at the variable ‘Recall’ and its range in the supplementary material). It might be worth adding a clarifying sentence or two in the main text to walk readers through the analysis process a little more slowly.**

We have added a few sentences to explain how model comparisons work: “For our analyses of average severity ratings, we adopted a model comparison approach (Burhmam and Anderson, 2002). We first ran a null linear model that used a fixed intercept (i.e. the mean of the outcome variable) to predict each participants’ average severity ratings. We compared this null model with two linear regression models directly derived from our hypotheses using Akaike’s Information Criterion (AIC, see Table 2). A lower AIC by a difference of two or larger was considered evidence of better fit to the data” (Lines 365-370).

We have also added a reference to a classical book on the use of AIC for model comparisons and statistical inference:

Burnham, K. P., & Anderson, D. R. (2002). Model Selection and Multi-Model Inference: A Practical Information-Theoretic Approach. Secaucus, US: Springer.

We have also added a few sentences to be clearer about our use of logistic regression for a proportional outcome and added a reference about how to conduct this type of regression model: “For our analyses of recall, we use the same model comparison approach as for the analyses of average severity ratings. As our outcome variable was the proportion of correctly recalled symptoms/side effects, we modelled the relationship between the predictors and the outcome variables using logistic regression models for a proportional outcome (Zuur, Ieno, Saveliev, Smitt & Walker, 2009, pp. 254-257). The fit of a null model was compared with our confirmatory models (see Table 3)” (Lines 395-401).

**R2 noted that pre-registration is a particular strength of the paper. They seem not to have located the scripts and data in supplementary material, but I think these will be much more findable when the paper is nicely formatted and online. I had a look and they seem clear and thorough to me.**

The data and the R scripts were submitted together with the manuscript. We want the data and R scripts to be accessible for everybody together with the article on PlosOne’s website.

**R2 did note that the dates of data collection don’t seem to be given in the paper, and I can’t find them either. Please could you specify the period of data collection in the main text?**

We have added a sentence (Line 263) with the dates of data collection (5^th^ March and 31^st^ March 2018).

**R2 also suggests that there should be a clearer marking of analyses that are and are not part of the pre-registered plan. I note that these are marked in the R scripts, but would ideally like to see a brief note of this in (the results section of) the main text as well.**

We have provided a more extended explanation of the changes conducted in the data analyses with respect to the preregistered analyses at the beginning of the Results section. Specifically, we report that we are not reporting regression models with interaction terms without main effect terms due to their controversial nature in statistics. We explain that instead we report regression models with both main effect and interaction terms. We also note that this change has not qualitatively affected the results and provide both analyses as supplementary materials so that the readers can check this by themselves.

**Power
Finally, R2 noted the age-old problem of inferring a negative from a null result, and suggested that if the ‘true’ effect is small then N=202 might actually miss it. Please could you tweak the sentence that R2 notes in their review and any similar ones accordingly. An additional suggestion from me would be to discuss the range of plausible effect sizes according to the analysis, as well as reporting whether or not a predictor is part of a best-fitting model. Joe Stubbersfield does this in the main text and supplementary material of this paper: Stubbersfield, J. M., Dean, L. G., Sheikh, S., Laland, K. N., & Cross, C. P. (2019). Social transmission favours the ‘morally good’over the ‘merely arousing’. Palgrave Communications, 5(1), 3.**

As suggested, we have qualified the statement “The lack of evidence for the omission bias indicates the nonexistence of such a bias or the elimination of its effects in modern environments” by changing it to “The lack of evidence for the omission bias MIGHT INDICATE the nonexistence of such a bias or the elimination of its effects in modern environments” and adding “Another possibility is that our between-subjects experiment design with a sample size of 202 participants might have been inadequate to detect a small or medium effect of omission bias. A replication of the current experiment with a larger sample size might help to clarify this possibility”(Lines 478-483).

The best fitting model for average severity ratings is the null model, so the best-fitting model does not include any of the predictors of interest. For proportion of correct recall, the best fitting model did include our predictors of interest (Condition and Vaccination attitudes) and their interaction together with Reaction Time. However, the model fit was similar to the control model (with Reaction Time as unique predictor). Consequently, we do not think it is necessary to report this.

**Gender of participants
I found the (quite interesting!) rationale for testing only female participants in the supplementary material, but I think it’s worth mentioning briefly in the main text because many readers will wonder about it.**

We have now included the sentences about our selection of only female participants in the main text (Lines 241-249)

# # REVIEWER 1

**1. Regarding Pre-registration

The preregistration of the analysis is a great thing and the authors could make more of it (since it is a strength of an analysis with several DVs and multiple plausible analysis pathways for the exact analysis to be pre-declared).

Regardless of whether the authors decide to emphasise the pre-registration they should

A Clearly distinguish pre-registered analyses from exploratory analyses in the results**

We have provided a more extended explanation of the changes conducted in the data analyses at the beginning of the Results section. Specifically, we report that we are not reporting regression models with interaction terms without main effect terms due to their controversial nature in statistics and instead we report regression models with both main effect and interaction terms. We also note that this change has not qualitatively affected the results and provide both analyses as supplementary materials so the readers can check this by themselves (Lines 341-349)

**B Clarify the timeline: the ethics for this project was given June 2016, the pre-registration is dated Dec 2017, but timestamped Feb 2018. When was the data collected? This information should be declared**

The ethical approval was given in June 2016. We did a pilot study for the first author’s Master dissertation. Later we pre-registered the experiment in February 2018. We collected the data in 5^th^ March 2018 and 31^st^ March 2018.

We have added a sentence (Line 263) with the days of data collection.

**2. Open Dat

Why not make the data (and final analysis code) from this study available?**

The data and final analysis code will be available as supplementary materials at the moment of publication. These files were uploaded in our initial submission. See omission_bias_experiment.csv for the dataset and Supplementary Materials E for the R Script.

**3. Risk of false negatives

The authors should discuss the possibility that their null results are false negatives. In particular, a statistical power analysis of a between subjects contrast will reveal that n=202 is not actually that many for detecting small to medium effects. The consequence of this must be to weaken the conclusion that this study is evidence of lack of omission bias (i.e. it elevates the plausibility that the study is merely a lack of evidence regarding the presence of an omission bias). So, for example, a sentence like this should be revised or qualified "The lack of evidence for the omission bias indicates the nonexistence of such a bias or the elimination of its effects in modern environments."**

As suggested we have qualified the statement “The lack of evidence for the omission bias indicates the nonexistence of such a bias or the elimination of its effects in modern environments” by changing it to “The lack of evidence for the omission bias MIGHT INDICATE the nonexistence of such a bias or the elimination of its effects in modern environments” and adding “Another possibility is that our between-subjects experiment design with a sample size of 202 participants might have been inadequate to detect a small or medium effect of omission bias. A replication of the current experiment with a larger sample size might help to clarify this possibility.” (Lines 478-483).

# # REVIEWER 2

**L210:**

**On the influence of vaccination attitudes on the memorization of vaccine related information Altay & Mercier 2018 tested the effect of “attitudinality” on memorization, and found that information perceived as being in favor of vaccination were not better memorized by participants in favor of vaccination than information perceived as being against vaccination (Experiment 2).**

As suggested, we have mentioned the lack of evidence for a confirmation bias as applied to vaccination in the findings of Altay and Mercier (2018): “Altay and Mercier (2018) did not find that participants with pro-vaccination attitudes recall better perceived pro-vaccination messages than perceived anti-vaccination information, thus failing to support the confirmation hypothesis as applied to vaccination. Together with that study, our study is one of the first to test whether the confirmation bias plays a role in the transmission of vaccine-related information, and the first to directly compare omission and confirmation biases.”(Lines 198-203)

**L317:**

**I would put figure 1 in supplementary materials and just give a description of the scale in one or two lines.**

We prefer to keep figure 1 in the manuscript, as it provides information to the readers of how each symptom/side effect was displayed to the participants.

**Section 3.2.**

**I do not find the statistical analyses very intuitive but it’s probably because I’m not familiar with model comparisons using AIC in experimental designs. If other reviewers or the editor do not find the analyses easy to understand either, effort should be made by the authors to clarify this section.**

We have provided additional explanations and references to make our model comparison approach using AIC easier to understand (see response to editor’s comments above).

**Section 3.3.**

**Since participants contributed to multiple data points (if I understood well), participants need to be added as a random factor in the logistic regression to account for the non-independence of the paired data (even though it will probably not change the results).**

Participants did not provide multiple data points as we are using average data (e.g. average severity ratings, average reaction times and proportion of correct recall) for our analyses in line with the survival processing experimental paradigm by Nairne, Thompson, & Pandeirada, (2007).

**L451:**

**Minor comment: The critic on the need to do correction for multiple comparison is sound, but I don’t think using Bonferroni is, the test being too conservative (it increases too much the chances of making false negative). Would we find the same results with holm correction technique?**

The Holm-Bonferroni technique yields the same results as the Bonferroni technique (6 significant tests). In the original article, we said 5 significant tests, but this was a typo. It’s 6. We report Holm-Bonferroni in the manuscript instead of Bonferroni (Lines 465-468).

**Conclusion:**

**I agree with the following sentence “Instead, the messages transmitted by people with anti-vaccination beliefs might spread due to having particular characteristics (e.g. being simple, concrete, emotional, unexpected, narrative, etc.) that make them especially “sticky” in human minds (Heath & Heath, 2008)”.**

**But I don’t see how it can explain anti-vaccination attitudes. At some point people need to find these messages convincing to have anti-vaccination attitudes. However, it seems that most people need not be convinced that vaccines are bad but rather that they are good.**

**This is why I find the following sentence not very convincing (L497-499):**

**“The lack of convincing evidence for the omission bias suggest that the spread of anti-vaccination messages might not be explained by the existence of a universal cognitive bias acting against the practice of vaccination (e.g. Miton & Mercier, 2015)”**

**Omission bias is surely a very bad candidate to explain anti-vaccination attitudes (and the authors made a convincing point) but the disgust hypothesis is not completely ruled out, even though it lacks empirical support. But many other properties of the human mind (and of our current environment) could explain resistance to vaccination. The authors only ruled out omission bias, not all the others possible cognitive bias.**

**The two explanations are compatible and probably true: vaccination can be counter-intuitive and pro-vaccination messages could be also less attractive than anti-vaccination messages.**

**As a side note, I tested the second hypothesis during my master thesis (in French unfortunately: YESILALTAY, S. (2018). ÉTUDE DES DÉTERMINANTS COGNITIFS DES ATTITUDES VACCINALES.) and found that vaccines descriptions containing disgust-related information (but not threat-related information) were better memorized than valence-neutral information (Experiment 2). Which supports the hypothesis made by the authors in the conclusion. The vignettes looked like that:**

**Disgust : Poliomyelitis (Polio) is a highly contagious and infectious viral disease that usually spreads from person to person through infected fecal matter entering the mouth.
Control : Poliomyelitis (Polio) is a contagious viral disease that can spread from person to person by oral transmission like eating infected food or drinking infected water.**

**Threat : As long as a single individual remains infected, children and adults worldwide are at risk of contracting polio.
Control : Nowadays, polio can only be found in remote areas of Afghanistan, Nigeria, and Pakistan.**

You are right, the study does not provide any evidence to rule out the disgust-antivaccination link. We have changed the sentence to be more specific to the omission bias: “The lack of convincing evidence for the omission bias suggest that the spread of anti-vaccination messages might not be explained by an omission bias acting against the practice of vaccination” (Lines 510-512).

We have also removed the comment about the lack of convincing evidence for disgust sensibility as this is not the topic of the article.

**Footnote 1:**

**Replace**

**“The evidence linking disgust sensibility with the formation of anti-vaccination attitudes is also rather week.”**

**by**

**“The evidence linking disgust sensibility with the formation of anti-vaccination attitudes is also rather *weak*.”**

We have deleted this footnote as our article is not about disgust sensibility and anti-vaccination beliefs.

**L502-504:**

**The last sentence of the article raises an important point but references to the literature on vaccine communication are missing, e.g.**

**Mckinnon, M., & Orthia, L. A. (2017). Vaccination communication strategies : What have we learned , and lost , in 200 years ?, *16*(03), 1–16.**

**Bruine de Bruin, W., Wallin, A., Parker, A. M., Strough, J., & Hanmer, J. (2017). Effects of anti-versus pro-vaccine narratives on responses by recipients varying in numeracy: A cross-sectional survey-based experiment. Medical Decision Making, 37(8), 860-870.**

**Nyhan, B., Reifler, J., Richey, S., & Freed, G. L. (2014). Effective Messages in Vaccine Promotion: A Randomized Trial. *Pediatrics*, *133*(4), e835–e842. https://doi.org/10.1542/peds.2013-2365**

**And also probably:**

**Aarøe, L., & Petersen, M. B. (2018). Cognitive Biases and Communication Strength in Social Networks : The Case of Episodic Frames, 1–21.** [**https://doi.org/10.1017/S0007123418000273**](https://doi.org/10.1017/S0007123418000273)

We don’t think we need to provide specific references for the literature on vaccine communication as the article focuses on cognitive biases, not in interventions to make pro-vaccination messages spread better. However, we have added the article by Bruine de Bruin et. al. (2017) as it was relevant to the last sentence of the article.
